# Effects of Drying Methods on Antioxidant, Anti-Inflammatory, and Anticancer Potentials of Phenolic Acids in Lovage Elicited by Jasmonic Acid and Yeast Extract

**DOI:** 10.3390/antiox10050662

**Published:** 2021-04-24

**Authors:** Urszula Złotek, Sławomir Lewicki, Anna Markiewicz, Urszula Szymanowska, Anna Jakubczyk

**Affiliations:** 1 Department of Biochemistry and Food Chemistry, University of Life Sciences in Lublin, Skromna 8, 20-704 Lublin, Poland; urszula.szymanowska@up.lublin.pl (U.S.); anna.jakubczyk@up.lublin.pl (A.J.); 2 Department of Microwave Safety, Military Institute of Hygiene and Epidemiology, 01-163 Warsaw, Poland; slawomir.lewicki@wihe.pl (S.L.); anna.kucza7@gmail.com (A.M.); 3 Department of Medicine, Faculty of Medical Sciences and Health Sciences, Kazimierz Pulaski University of Technology and Humanities, 26-600 Radom, Poland

**Keywords:** lovage, elicitation, phenolic acids, antioxidant activity, potential anti-inflammatory potential, anticancer properties

## Abstract

The study presents the effect of drying methods (traditional, convection, microwave, and freeze-drying) on the content and bioactivity (determined as antioxidative, anti-inflammatory, and antiproliferative potential) of potentially bioavailable fractions of phenolic acids contained in lovage elicited with jasmonic acid (JA) and yeast extract (YE) and in untreated control leaves. The highest amount of syringic acid was recorded in the convectionally dried lovage samples, while ethanolic extracts from lyophilized lovage had the highest content of protocatechuic and caffeic acids. The drying method significantly influenced the tested properties only in some cases. The traditional drying resulted in lower antioxidant potential, while convectional drying caused a reduction of the lipoxygenase inhibition ability of the samples after simulated digestion. Samples containing the control and elicited lovage leaves dried with convectional and traditional methods exhibited the highest cytotoxicity against a prostate cancer epithelial cell line.

## 1. Introduction

The food industry has recently become increasingly interested in herbs due to their organoleptic, pro-health, and preservative properties. The *Apiaceae* family is one of the groups of herbal plants comprising many herbs with culinary and medical importance. Herbs from this family are rich in bioactive compounds that are characterized by many health-promoting properties, i.e., antioxidant, antibacterial, hepatoprotective, vasorelaxant, anti-inflammatory, and antitumor activities [1]. Lovage, i.e., one of the important plants from the *Apiaceae* family, is especially popular in cooking, mainly for its aromatic properties [2]. Additionally, lovage has many bioactive properties, which predispose it to be used in other applications, e.g., in medicine, as indicated in some publications. This herb has been used in folk medicine mainly due to its carminative, spasmolytic, and diuretic effects, but there is ample information regarding other pro-health properties of lovage, e.g., antioxidant, antibacterial, hepatoprotective, and anticancer activities [2,3]. When considering this fact and the growing consumers’ interest in natural spices related to the growing awareness of the impact of food on human health, enhancement of the health potential of food of plant origin is still an important issue.

Previous studies have indicated that elicitation can be an effective and safe method for the improvement of the health-promoting properties of selected herbs, including lovage [3,4,5]. Elicitation with jasmonic acid and yeast extract can improve some biological activities via an enhancement of the production of phenolic acids in fresh lovage leaves, as shown in our previous study [3,5].

In turn, there is no information regarding the effect of the drying methods on preservation of these properties in elicited herbs. Drying, which is commonly used in food technology, is a very valuable method for the enhancement of the storability of herbs after harvest. However, it can cause changes in the organoleptic quality, which has been analyzed in many studies [6,7]. Drying may also affect the health properties of herbs, but there is very limited information on this subject (especially for elicited herbs).

The aim of the present study was to evaluate the influence of drying methods on the content and bioactivity (determined as antioxidative, anti-inflammatory, and antiproliferative potential) of potentially bioavailable fractions of phenolic acids that are contained in lovage leaves elicited with jasmonic acid and yeast extracts.

## 2. Materials and Methods

### 2.1. Growth Conditions

**Figure 1:**
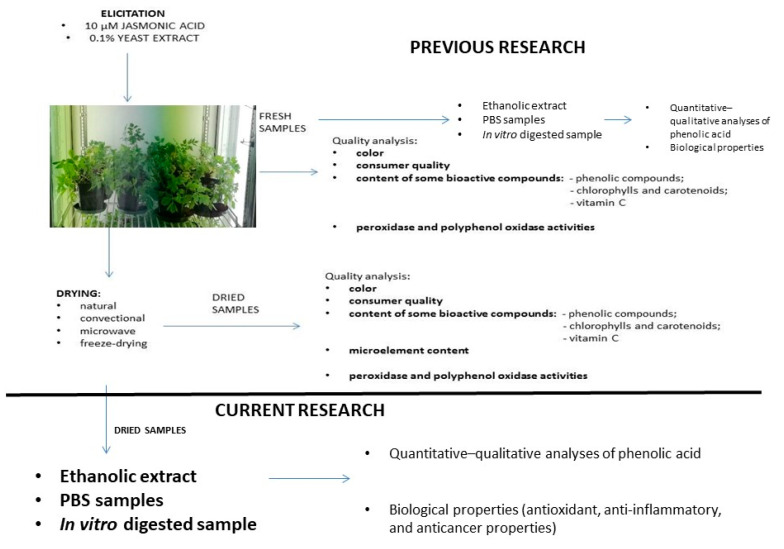
Scheme of the experimental procedure.

Lovage (*Levisticum officinale* Koch. cv. Elsbetha) leaves growing as a control and elicited with 10 µM of jasmonic acid (JA) and 0.1% yeast extract (YE) were the plant materials that were used in this study. The growth conditions and elicitation method were described in our previous manuscript [5]. The concentrations of the elicitors were selected based on our previous study [5], which indicated that 0.1% yeast extract (YE) and 10 µM of jasmonic acid (JA) proved to be the most effective concentrations of the elicitors for lovage plants. The plants were collected twenty-five days after the elicitation. Next, the plant materials were assigned into four groups, which were subjected to the different drying conditions, as in Figure 1.

### 2.2. Drying Techniques

Fresh herbs were dried using four techniques: natural (traditional) drying (in a darkened room, a temperature of 20 °C to 22 °C, for approximately seven days), convection drying (in a drying oven at 40 °C for approximately 5 h), microwave drying (in a laboratory microwave dryer, microwave power 360 W at 20 °C, for ca. 5 min), and freeze-drying (in a lyophilizer at a temperature of −49 °C and pressure of 0.045 mbar) [8,9].

### 2.3. Preparation of Extracts

#### 2.3.1. Ethanolic Extracts

The ethanolic extracts were prepared for further analysis (0.3 g dw dissolved in 15 mL of 70% (*v/v*) acidified ethanol (0.1% HCl) were subjected to sonication at 30 °C for 1 h and centrifugation at 9000× *g* for 30 min).

#### 2.3.2. PBS Extracts

For the preparation of buffer extracts, dried leaf tissue (0.5 g) was homogenized, extracted for 30 min with 20 mL of PBS buffer (phosphate buffered saline, pH 7.4), and then centrifuged at 9000× *g* for 20 min Next, the residues were extracted again with 20 mL of PBS buffer. The supernatants were combined and adjusted to a final volume of 50 mL of with PBS buffer.

#### 2.3.3. In Vitro Digestion

In vitro digestion was performed, as described previously by Gawlik-Dziki et al. [10]. The dried leaf tissue (0.5 g) was homogenized in a Stomacher Laboratory Blender for 1 min to simulate mastication in the presence of 5 mL of a simulated saliva solution containing 7 mM NaHCO_3_, 0.35 mM NaCl (pH 6.75), and α-amylase (E.C. 3.2.1.1., 200 U per mL of enzyme activity). Subsequently, the mixture was stirred for 10 min at 37 °C in darkness. For gastric digestion, the solution was adjusted to pH 2.5 with 1 M HCl and 15 mL of 300 U/mL of pepsin (from porcine stomach mucosa, pepsin A, EC 3.4.23.1) in 0.03 mol/L NaCl, pH −1.2, were added. The reaction was carried out for 60 min at 37 °C. Next, the solution was adjusted to pH 7 with 1 M NaOH and 15 mL of a mixture of a 0.7% solution of pancreatin and a 2.5% solution of bile extract were added. The incubation was carried out for 120 min at 37 °C in darkness. Thereafter, the samples were centrifuged and the supernatants (gastrointestinally digested extracts; GD extracts) were used for further analysis.

### 2.4. Analysis of Phenolic Acids

Quantitative–qualitative analyses of phenolic acid extracts were performed while using a VarianProStar HPLC system separation module (Varian, Palo Alto, CA, USA) equipped with a Varian ChromSpher C18 reverse-phase column (250 mm × 4.6 mm) and a ProStar diode array detector. The column thermostat was set at 40 °C. All of the analyses were carried out with 4.5% acetic acid (solvent A) and 50% acetonitrile (solvent B) mobile phases at a flow rate of 0.8 mL min^−1^. At the end of the gradient, the column was washed with 50% acetonitrile and then equilibrated to the initial condition for 10 min. Quantitative determinations were conducted by calculation of an external standard, using calibration curves of the standards. Detection was performed at 270 nm and 370 nm. Phenolic acids that were present in the sample were identified by comparing the retention times and UV-visible absorption spectra with those of the standard compounds. Phenolics were expressed as micrograms per gram dry weight (DW) [11].

### 2.5. Antioxidant Activities

#### 2.5.1. Free Radical Scavenging Assay

The free radical scavenging activity was determined using 2,2′-azino-bis[3 -ethylbenzothiazoline-6-sulphonic acid (ABTS^●+^) as a source of free radicals as in Re et al. [12]. The antioxidant activity was expressed as μmol of Trolox per gram of dry weight (DW) (TEAC, Trolox equivalent antioxidant activity).

#### 2.5.2. Ferric Reducing Antioxidant Power

Ferric reducing antioxidant power (RP) was determined according to the methods that were described by Oyaizu [13]. The reducing power was expressed as a Trolox equivalent (TE) in µmol of Trolox per gram of dry weight (DW).

#### 2.5.3. Chelating Power

The chelating power (CHP) was determined using the method that was developed by Guo et al. [14]. The percentage of inhibition of the ferrozine-Fe^2+^ complex formation was calculated using the formula:

% inhibition = [1 − A_A_/A_C_] × 100

where:

A_C_—the absorbance of the control (the solvent instead of the extract), A_A_—absorbance of the sample

The chelating power was expressed as an EDTA (Ethylenediaminetetraacetic acid) equivalent in µg EDTA per g of dry weight (DW).

### 2.6. Determination of Potential Anti-Inflammatory Properties

#### 2.6.1. Lipoxygenase Inhibitory Activity

The method described by Szymanowska et al. [15] and adapted to the BioTek Microplate Reader was used to analyze the effect of the different concentrations of the control and the elicited lovage extracts (ethanolic, PBS and obtained after the simulated digestion) on lipoxygenase activity. Based on the relationship between the concentration of the analyzed extract and the degree of inhibition of LOX activity, a curve was drawn, from which the EC_50_ value, i.e., an extract concentration (mg DW/mL) providing 50% inhibition, was determined.

#### 2.6.2. Cyclooxygenase-2 Inhibitory Activity

A COX Activity Assay kit from Cayman Chemical Company was used to determine the inhibition of cyclooxygenase-2 activity by the analyzed extracts. The determination was carried out according to the procedure that was specified by the manufacturer of the kit. Based on the degree of inhibition of COX-2 activity by various concentrations of the analyzed extracts, the EC_50_ value, i.e., a concentration (mg DW/mL) providing 50% enzyme inhibition, was determined.

### 2.7. Anticancer Properties

The studies were performed on two cancer cell lines: gastric epithelial cell line NCI-N87 (ATCC^®^ CRL5822 ™) and prostate cancer line VCaP (ATCC^®^ CRL-2876 ™). Healthy prostate epithelial cells HPrEC (ATCC^®^ PCS-440-010™) were used as a control. All of the cell lines were purchased from the American Type Culture Collection (ATCC), University Boulevard, Manassas, VA, USA.

#### 2.7.1. Cell Culture

The studied cells were cultured in the same conditions, as described in our previous publication [3].

#### 2.7.2. MTT and NR Tests

The MTT and NR assays were performed, as described previously [3,16].

#### 2.7.3. Cell Viability and Type of Cell Death

The study was conducted according to the procedure that was specified by Leśniak et al. [17] and described in detail by [3].

#### 2.7.4. Cell Cycle

After trypsinization (0.05% trypsin, 3–7 min, 37 °C), the cells were seeded onto a 48-cell culture plate. After 24 h, the cells were washed with the PBS solution and appropriate culture medium with the studied dried lovage leaf extracts at the concentrations of 0.1 and 20 mg/mL was added. After 24 h, the cells were trypsinized (0.05% trypsin, 3 min, 37 °C), centrifuged (300× *g*, 5 min), washed twice with the PBS solution, and then centrifuged again (300× *g*, 5 min). The cell pellet was then resuspended in 1 mL of a cold 70% ethanol/PBS solution (4 °C) and left in a freezer (−20 °C) for permeabilization of the cell membrane until the time of analysis (min. one day, max. 30 days). On the day of analysis, the cells were centrifuged (300× *g*, 5 min) and suspended in 300 μL of stain buffer (PI/RNase Staining Buffer, BD Warsaw, Poland). Subsequently, iodide propidium (PI, Bioscene Warsaw, Poland) was added for 15 min at room temperature. Cell cycle analysis was performed using a flow cytometer (FACS Calibur, BD, San Jose, CA, USA) (25,000 cells stopped the acquisition) and calculations were conducted using FCS Express program (De Novo Software, Pasadena, CA, USA). The results are presented as mean ±SD of three independent experiments (n = 6).

### 2.8. Statistical Analysis

All of the the determinations were performed in triplicate. Statistical analysis was performed using STATISTICA 7.0 software for a comparison of means in ANOVA with post-hoc Tukey’s HSD (honestly significant difference) test at the significance level *p* < 0.05.

The data obtained from the experiment on cancer cells were checked for the normality of distribution (Shapiro–Wilk test). The level of statistical significance was calculated: in the case of normal distribution, one-way ANOVA with Bonferroni correction and Student’s *t* test were used; otherwise, non-parametric one-way ANOVA with correction Kruskal–Wallis and Mann–Whitney tests were employed. The data were analyzed using the GraphPad Prism program (version 5, GraphPad Software, Inc., La Jolla, CA, USA) at a significance level of *p* < 0.05. The data were also evaluated using Pearson’s correlation coefficients to identify the relationships between phenolic acid content and selected biological activities of the studied samples.

## 3. Results

### 3.1. Phenolic Acid Analysis

The drying method influenced the content of some phenolic acids, e.g., the highest amount of syringic acid was recorded in the convectionally dried lovage samples, while the ethanolic extracts from lyophilized lovage had the highest content of protocatechuic and caffeic acids. Ferulic acid was only identified in the ethanolic extracts from dried lovage, with its highest amount being detected in the microwave-dried lovage samples (Table 1).

The chemical (ethanolic) extracts were characterized by the highest content of caffeic and sinapic acids, while an opposite trend was observed in the case of *p*-hydroxybenzoic acid—the highest concentration was detected in the in vitro digested samples, as in Table 1.

The elicitation with JA caused a statistically significant increase in the content of most of the analyzed phenolic acids. In all samples of lovage dried with the three methods (freeze-drying, microwave drying, and traditional drying), elicitation with jasmonic acid resulted in a statistically significant increase in the content of protocatechuic, caffeic, syringic, ferulic, and salicylic acids. In addition, lovage dried with all of these methods was characterized by increased content of sinapic acid, as seen in Table 1.

In some cases, elicitation with the yeast extract also resulted in an increase in the content of some identified compounds. This can be noticed in particular in the ethanolic extracts from the freeze-dried and microwave-dried lovage. In the ethanolic extracts from the freeze-dried lovage, the elicitation with YE caused a 0.65-fold, 0.75-fold, 0.30-fold, over two-fold, and over two-fold increase in the content of protocatechuic acid, caffeic acid, ferulic acid, sinapic acid, and salicylic acid, respectively. In the case of the microwave-dried lovage leaves, the content of caffeic, syringic, vanillic, ferulic, sinapic, and salicylic acids in the ethanolic extracts of herbs treated with the yeast extract increased by 16.4%, 30.8%, 15.3%, 24.6%, 19.9%, and 19.4%, respectively, as in Table 1. Additionally, in the case of the convectionally dried herbs, the elicitation with YE resulted in an increase in the content of protocatechuic acid, caffeic acid, syringic acid, vanillic acid, sinapic, acid, and salicylic acid in the PBS extract, as in Table 1.

### 3.2. Antioxidant Activities

The lovage drying method did not significantly affect the antioxidant properties of the tested extracts from the control and elicited herbs, with the exception of the reducing power (in this case, the traditional drying resulted in substantially lower antioxidant potential of the samples after simulated digestion), as summarized in Table 2. It should be noted that a significant enhancement of all antioxidant properties of the GD extracts from the control and elicited lovage was detected, regardless of the drying method used (compared to the ethanolic and PBS-extracts), as seen in Table 2. Additionally, in some cases, the elicitation with JA and YE improved the antioxidant properties of the potentially bioavailable fraction and the PBS extracts of phenolic acids from dried lovage leaves. The highest antiradical properties of the potentially bioavailable fraction of phenolic compounds estimated against ABTS were detected in the samples of convectionally dried lovage treated with JA (the activities increased by 58.52% when compared to the control), as in Table 2. Additionally, the reducing power of samples after in vitro digestion was statistically significantly increased by the elicitation in some cases. The GD extract from JA-elicited freeze-dried lovage was characterized by 17.34% higher RP compared to the control, while the YE elicitation increased this ability by 23.46% in the GD samples of convectionally dried lovage (Table 2).

The PBS-extractable phenolic compounds from the JA-elicited lovage showed a statistically significantly higher ability to neutralize free radicals ABTS (in the case of traditionally and microwave-dried herbs). The same result was obtained in the case of the, microwave-dried, traditionally, and convectionally dried YE-elicited lovage samples (Table 2).

However, the elicitation with JA and YE did not have a positive effect on the ability to chelate iron ions by the tested dried lovage extracts, as in Table 2.

The statistical analysis indicated the content of *p*-hydroxybenzoic acid was correlated significantly and positively with the antioxidant activities—(R^2^ = 0.63; R^2^ = 0.72; R^2^ = 0.76 for ABTS, RP and CHP, respectively)—Table 3.

### 3.3. Lipoxygenase and Cyclooxygenase 2 Inhibition

The extracts from dried lovage leaves exhibited potential anti-inflammatory activity reflected in the inhibition of pro-inflammatory enzymes, such as lipoxygenase and cyclooxygenase 2, as shown in Table 2. Convectional drying, as compared to the other drying methods, resulted in a reduction of the LOX inhibition ability by the PBS and GD samples of the control and elicited lovage. The COX2 inhibition by the ethanolic extracts was significantly lower in the case of the convectionally and microwave dried samples, as shown in Table 2. It should be noted that the potentially bioavailable fractions of phenolic acids (GD extracts) were characterized by a significantly higher ability to inhibit COX2 in comparison to the PBS extract, while such a relationship for the ability to inhibit LOX activity was only observed in the convectionally dried lovage samples (Table 2). However, the elicitation with JA and YE did not have a positive effect on these properties of the potentially bioavailable phenolic acids from dried lovage leaves—only the samples from convectionally dried plant material subjected to in vitro digestion and JA and YE elicitation exhibited higher COX2 inhibitory ability (ca. 40% and 53%, respectively) than the control, as seen in Table 2.

It should be also noticed that, in the case of the ethanolic extracts, the elicitation with JA and YE caused an increase in the ability to inhibit COX2 (except for the ethanolic extracts from the convection-dried lovage leaves, where an opposite trend was observed). The greatest increase in this activity (over five-fold) was observed in the case of extracts from the JA-treated freeze-dried lovage (Table 2).

In the case of the ethanolic extracts from the microwave-dried and traditionally dried lovage leaves, the elicitation with JA and YE also resulted in an increase in the ability to inhibit LOX, as shown in Table 2.

The analysis of correlations only indicated a significant positive relationship between LOX and COX2 inhibition and phenolic content for the p-hydroxybenzoic acid content (Table 3).

### 3.4. Influence of Analyzed Extracts on Healthy and Cancer Cell Lines

The PBS extracts of the control and elicited lovage leaves dried with all of the tested methods in the tested concentrations (0.1–20 mg dw/mL) had no effect on the number of cells (in MTT and NR assays), viability (no impact on apoptosis or necrosis), or percentages of cell cycle phases (G1, S, or G2) in the gastric cancer NCI-N87 (ATCC^®^ CRL5822 ™), prostate cancer VCaP (ATCC^®^ CRL-2876 ™), and healthy prostate HPrEC (ATCC^®^ PCS-440-010 ™) cell lines, as compared to cells that were not exposed to the extracts (Table 4). In contrast, the ethanolic extracts and in vitro digested samples exerted a significant influence on these properties (Table 4 and Supplement S1–S4).

The ethanolic extracts of the studied samples (control and elicited) caused a significant reduction of the number of healthy prostate epithelial HPrEC cells, but only at the concentrations of 10 and 20 mg/mL (Figures S1–S3, S37–S39 and S55–S57). It should be noted that this effect was significantly lower in the case of samples that were obtained from the microwave-dried lovage (CM-E, JAM-E, YEM-E samples), as in Table 4; Figures S19–S21. At the highest concentration, all of the ethanolic extracts caused apoptosis. With the exception of the microwave-dried lovage samples, this was associated with changes in the cell cycle (an increased percentage of cells in the S phase with a reduction of cells in the G1 phase). It should also be noted that there were no significant changes in the influence on the healthy prostate epithelial HPrEC cells between the control sample and samples elicited with jasmonic acid (JAL-E) or with the 0.1% yeast extract (Figures S1–S3, S37–S39 and S55–S57). A similar effect on the healthy prostate epithelial HPrEC cells was also observed in the case of the in vitro digested samples; however, the effect depended on the method of drying (Table 4; Figures S4–S6, S22–S24, S40–S42 and S58–S60). In the case of the in vitro digested samples from the lyophilized and convectionally dried lovage, a significantly lower effect on the cytotoxicity parameters (proliferation and viability) was observed when compared to the ethanolic extracts, but the effect of the changes in the cell cycle was preserved, as seen in Table 4. The strongest negative effect on the healthy cells (comparable to that of the ethanolic extracts) was noted in the case of the digested samples from the traditionally dried lovage (CT-GD, JAT-GD, YET-GD), but these samples caused opposite changes in the cell cycle (a reduction of the percentage of S-phase cells), as in Figures S40–S42. In turn, the GD samples from the microwave-dried lovage showed no cytotoxic effect on the HPrEC cells in the proliferation assays—there was only a slight, but significant, increase in the percentage of apoptotic cells after the JAM-GD treatment (21.5% vs. 16.6% when compared to the non-treated cells, *p* = 0.0112). However, at the highest concentration (20 mg/mL), a significantly higher percentage of G2 phase cells was noted (Figures S22–S24).

The ethanolic extracts of the studied samples (control and elicited) at the concentrations of 10 and 20 mg/mL caused a decrease in the number of cancer gastric epithelial NCI-N87 cells measured by the MTT and NR assays (Figures S7–S9, S25–S27, S43–S45 and S61–S63). The strongest effect was observed for ethanolic extracts from the traditionally dried lovage (the largest decrease in the number of cells as well as the cytotoxic effect when samples at the lower concentration of 5 mg/mL were used). The highest concentration of ethanolic extracts (20 mg/mL) affected the viability of the cells by increasing apoptosis (over 90% cells—extracts from the lyophilized lovage (CL-E, JAL-E, YEL-E), 95% cells—extracts from the microwave- and traditionally-dried samples (CM-E, JAM-E, YEM-E, CT-E, JAT-E, YET-E), and 98% cells—extracts from the convectionally dried samples- CC-E, JAC-E, YEC-E). Additionally, a reduction of the number of necrotic cells and, in some cases, also changes in the cell cycle were observed (Table 4). The increased percentage of G1 cells that were associated with the reduction of the percentage of G2 cells observed after the addition of the CL-E and JAL-E samples may be a result of the apoptosis process. It is noteworthy that, after the addition of ethanolic extracts from the convectionally dried lovage, there were no changes in the cell cycle in the cancer gastric epithelial NCI-N87 cells (Table 4). Generally, there were no significant changes in the influence on NCI-N87 cells between the control and elicited samples, with some exceptions. After the application of the highest concentrations of the JAL-E and JAM-E extracts, a significant reduction in the percentage of apoptotic cells was observed when compared to extracts from the control lovage (Figures S7–S8 and S25–S26).

The effect of the samples after in vitro digestion on the cancer gastric epithelial NCI-N87 cells was generally significantly lower than that of the ethanolic extracts, except for the GD samples from the traditionally dried lovage—CT-GD, JAT-GD, and YET-GD. In this case, the cytotoxicity of the GD-samples against the NCI-N87 cells was as high as that of the ethanolic extracts, as seen in Table 4, Figures S10–S12, S28–S30, S46–S48 and S64–S66. It should also be emphasized that, in the case of the GD samples from the microwave-dried lovage (CM-GD, JAM-GD, YEM-GD), there was no significant cytotoxic effect on the NCI-N87 cells, as in Table 4, Figures S28–S30. Additionally, the elicitation significantly changed these properties in some cases. Lower levels of apoptosis were detected after the addition of the JAL-GD and YEL-GD samples (40.7 and 15.1% of cells, respectively) in comparison to the CL-GD sample (46.8% of cells), as shown in Figures S10–S12. Additionally, the GD sample from the convectionally dried lovage elicited with the 0.1% yeast extract had no cytotoxic effect on the cancer gastric epithelial NCI-N87 cells (Table 4, Figure S66).

The prostate cancer epithelial VCaP cells turned out to be the least sensitive to the ethanolic and gastrointestinally digested extracts from both the control and elicited freeze-dried lovage leaves, as seen in Figures S13–S18. No cytotoxic effect was shown by the MTT assay in the case of the ethanolic extracts from the freeze-dried lovage, whereas the NR assay revealed a cytotoxic effect even for the 1 and 5 mg/mL concentrations of the samples. Additionally, in the case of the ethanolic extracts from the microwave-dried lovage, the cytotoxic effect in the NR test was observed, even for the low concentrations of the extracts used (0.1 and 1 mg/mL). It has to be underlined that these results indicate that the traditionally and convectionally dried samples from the control and elicited lovage plants exhibited the highest cytotoxicity against the VCaP cell line, in comparison to the samples from plants that were dried with the other methods (Table 4). This was especially visible in the influence on apoptosis: all extracts—from the control and elicited lovage dried with these methods caused a large increase in the level of apoptosis—over 80% of apoptotic cells, as seen in Figures S49–S51 and S67–S69.

The cytotoxic effect of the GD samples from the convectionally dried lovage and the control lyophilized lovage sample on the prostate cancer epithelial VCaP cells was relatively lower than in the case of the ethanolic extracts. It should also be added that the GD-samples from the microwave-dried lovage (control and elicited) did not exert a cytotoxic effect on the VCaP cells (Table 4, Figures S16–S18, S34–S36, S52–S54 and S70–S72). Interestingly, the GD samples from the elicited freeze-dried lovage (JAL-GD and YEL-GD) showed much higher cytotoxicity towards the VCaP cells than the GD samples from the control lovage, as shown in Figures S16–S18. A higher apoptosis level was found after treatment with the YEL-GD extract (72.8%) than JAL-GD (65.8%). In both of the elicited samples, a lower necrosis percentage (more than 65% reduction) and a lower mean level of apoptosis (40.7 and 15.1% of cells, respectively) were observed. There were also significant changes in the cell cycle phases. Generally, the percentage of cells in the G1 phase was increased and the number of S and G2 phase cells was reduced. Interestingly, the results for both GD samples from the elicited lyophilized plants (JAL-GD and YEL-GD) also produced changes in the cell cycle phases at the lowest concentration studied (0.1 mg of extracts/mL). Elicitation with jasmonic acid also increased the cytotoxicity against the VCaP cells exhibited by the in vitro digested convection-dried lovage samples (Figures S70–S71). The percentage of apoptotic cells amounted to 50.1% after the JAC-GD exposure, but it was only 12.3% in the case of application of the control lovage samples (CC-GD). In turn, the YEC-GD samples showed no cytotoxicity against the prostate cancer epithelial VCaP cells (Figure S72).

The ability to inhibit the proliferation and viability of the studied cell lines was positively correlated with the content of almost all identified phenolic acids, but a statistically significant positive correlation was only observed in the case of ferulic acid, sinapic acid, and salicylic acid (Table 3).

## 4. Discussion

Our recent research has confirmed that JA and YE elicitation exerts positive effects on phenolic acid biosynthesis, and it has revealed some pro-health properties of the potentially bioavailable fraction of phenolic acids from fresh lovage leaves [3]. However, the food industry very often needs dried lovage leaves. Therefore, the influence of drying methods on these properties of elicited lovage leaves is a very important issue, but no such research has been conducted so far. Drying can significantly extend the shelf life of herbs. Unfortunately, it may also cause some negative changes in the organoleptic or pro-health quality of herbal plants. [7,18]. Previous research indicated that drying caused significant losses of some bioactive compounds, e.g., vitamin C and plant pigments (carotenoids and chlorophylls). Additionally, it should be noted that phenolic compounds that were present in elicited and control lovage leaves turned out to be the least sensitive to degradation during the drying process [9]. Therefore, this group of bioactive compounds will determine the health-enhancing properties of dried lovage. In contrast, in the research conducted by Lim and Murtijaya [8], drying with three methods (oven drying, sun drying, and microwave drying) resulted in a significant decline in total phenolics in *Phyllanthus amarus*. A significant decrease in the content of polyphenols in *Cistus creticus* leaves after convection and microwave drying was reported earlier [7]. Noteworthy, these studies were focused on “chemical extracts”. In the present study, an attempt was made to investigate the potential bioavailability and health-promoting activity of phenolic acids contained in lovage leaves that were dried using various methods and subjected to the elicitation process with jasmonic acid and yeast extract. During digestion in the human body, phenolic compounds can be released from the food matrix or can be complexed with digestive enzymes, as indicated in scientific literature. This may result in the formation of some metabolites whose pro-health properties may differ from those of the initial material [19,20]. The bioavailability of phenolic compounds can be determined by several types of factors that are associated with the chemical structure of phenolics, interaction with the food matrix, and digestion conditions [19,21]. Seven phenolic acids (protocatechuic, *p*-hydroxybenzoic, syringic, vanillic, sinapic, salicylic, and caffeic acids) were detected in the PBS and GD samples from dried lovage leaves, as shown in Table 1. Additionally, ferulic acid was identified in the ethanolic extracts, as in Table 1. These results correspond to earlier studies on fresh leaves of JA- and YE-elicited lovage [3,5].

Our previous study indicated that elicitation with jasmonic acid increased the content of phenolic acids in PBS extracts from fresh lovage leaves; however, in the case of potentially available fractions of phenolic acids (GD samples), the treatment only increased the content of protocatechuic acid and *p*-hydroxybenzoic acid [3]. The highest levels of protocatechuic, caffeic, syringic, and salicylic acids were recorded in the GD samples of microwave-dried lovage (both elicited and control), when compared to the other drying methods, as shown by the analysis of the influence of various drying methods (traditional, convection, microwave, and freeze-drying) on the content of the potentially bioavailable fraction of phenolic acids (Table 1). In turn, the freeze-drying process yielded the highest levels of potentially bioavailable *p*-hydroxybenzoic acid and sinapic acid. Similarly, in studies on other herbs where three drying methods (conventional, microwave, and freeze-drying) were used, the best extraction results were observed in the case of microwave drying and freeze-drying of melissa and thyme, respectively [22]. Importantly, these results only apply to “chemical extracts” [22]. In the case of dried lovage leaves, the jasmonic acid elicitation caused an increase in all phenolic acids identified in the potentially available fractions of phenolic compounds (Table 1). Surprisingly, only in the case of lyophilization, the YE-elicited dried lovage leaves were characterized by a higher level of hydroxybenzoic and sinapic acids in the potentially bioavailable fraction of phenolic acids, in comparison to the control, as seen in Table 1. Therefore, it can be assumed that, especially in the case of the JA-elicited lovage leaves, drying had a positive effect on the release of phenolic compounds during the simulated digestion process. There are no similar data on the effect of drying on elicited herbs.

Drying that is associated with the use of high temperatures can degrade phenolic compounds. However, other studies also suggest that high temperature can induce some modifications that can positively influence the bioavailability of phenolics, e.g., degradation or modification of food matrix factors that may contribute to increasing the extractability of phenolic compounds during digestion [23]. In a study that was conducted by Kamiloglu et al. [24], oven-drying had a positive effect on the bioaccessibility of phenolic acids in tomato. Similarly, sun-drying enhanced the bioavailability of caffeic acid derivatives (50–60% increase) in figs, but negatively influenced the bioavailability of flavonoids [25]. Therefore, drying has a positive effect on bioavailability, especially in the case of phenolic acids, as confirmed in this paper.

Hydroxybenzoic acid is quite common in plants, but low amounts thereof are identified in food of plant origin, mainly because it is usually associated with cell wall structures, i.e., tannins or lignins [26]. The simulated digestion caused a significant increase in the content of this compound in the GD extracts (Table 1). This was probably related to the release of this acid from plant cell wall structures during the in vitro digestion process.

Many studies have indicated that phenolic compounds that are present in herbs largely determine their biological properties, i.e., the antioxidant and anti-inflammatory potential [27,28]; thus, it can be assumed that both the drying method and in vitro digestion of lovage leaves may have influenced these properties. The drying method had an impact only on the reducing power of the extract from lovage leaves dried with the traditional method, as summarized in Table 2. Similarly, vacuum drying did not have a significant effect on the antiradical activity of basil and peppermint in a study that was conducted by Dogan and Tornuk [29]. However, the drying methods significantly influenced the antioxidant activity measured as ferric reducing antioxidant power and oxygen radical absorbance capacity of dried herbs, such as rosemary, oregano, marjoram, sage, basil, and thyme, as demonstrated by Hossain et al. [30]. Among the three drying methods used (air-, freeze-, and vacuum oven-drying), the air-dried herbs had the best antioxidant properties. Similarly, drying at low temperature, e.g., sun-drying and 40 °C oven-drying, increased the total polyphenol contents and antioxidant capacity of peppermint, rosemary, and motherwort in a study that was conducted by Yi and Wetzstein [31]. Through the release of bioactive compounds (e.g., phenolics) from the food matrix, digestion can positively influence many biological properties, e.g., antioxidant, anti-inflammatory, antimicrobial, antimutagenic, hypoglycemic, and cardioprotective activities, as indicated in some research [32,33]. In the present study, the simulated digestion caused a significant enhancement of all the tested antioxidant activities and potential anti-inflammatory (expressed as COX2 inhibition) properties of the extracts from the control and elicited lovage, as in Table 2. However, an increase in LOX inhibition after the in vitro digestion was only detected in samples from convectionally dried lovage leaves (Table 2). These results partially correspond with the previous study on the biological properties of fresh lovage leaves after in vitro digestion. It was shown that the simulated digestion significantly improved some biological activities, in particular, the ability to inhibit the LOX and COX 2 [3]. This may suggest that drying had a negative effect on the bioavailability of compounds that are responsible for LOX inhibition.

Antioxidative and anti-inflammatory properties are also largely responsible for the anti-cancer properties of bioactive compounds [34]. A study that was conducted by Spréa et al. [2] showed cytotoxic potential of extract from lovage leaves against liver cancer cells. Similarly, in a study conducted by Bogucka-Kocka et al. [35], hydroalcoholic extract from lovage exhibited the cytotoxic potential against seven leukemia human cell lines and two normal cell lines. Noteworthy, these studies were conducted with the use of only decoction (water) and hydroethanolic extracts, and did not evaluate the potentially bioavailable fraction of lovage bioactive compounds.

In the present studies, the ethanolic extracts and GD samples from the lovage leaves (dried using all of the tested methods) had a cytotoxic effect against the gastric epithelial NCI-N87 cell line and the prostate VCaP cancer line, while samples before in vitro digestion (PBS) did not show such an effect (Supplementary Materials). Additionally, the ethanolic extracts showed generally greater cytotoxicity against the cancer cells and the healthy cell line, in comparison to the potentially available samples (GD samples), as in Table 4. This result was not confirmed by previous investigations, in which in vitro digested basil samples showed greater cytotoxicity against cancer cells than ethanolic extracts [33].

The drying method only significantly influenced the tested properties in some cases—the traditional and convectional dried lovage leaves showed significantly higher cytotoxicity against the prostate cancer epithelial cell line, in comparison to the other drying methods (Table 4).

Additionally, in some cases, elicitation had an effect on these properties of the analyzed herb. For example, samples from freeze-dried plants elicited with jasmonic acid (JAL-GD) and 0.1% yeast extract (YEL-GD) exhibited higher cytotoxicity against prostate cancer epithelial cells than the control plants, as in Supplementary Materials, Figures S13–S18. Additionally, in the case of the in vitro digested samples from convectionally dried plants, the highest cytotoxicity against the prostate cancer cell line was exhibited by samples from jasmonic acid-elicited lovage, whereas an opposite effect was observed in the yeast extract-elicited lovage samples. The information about the influence of elicitation on the anticancer properties of herbal extracts is very limited in the available literature. In the studies that were conducted by Złotek et al. [33], the elicitation of basil with arachidonic acid did not cause significant changes in the cytotoxic properties of the potentially bioavailable fraction of bioactive compounds against a cancer cell line.

In the literature, some phenolic acids, e.g., ferulic acid, *p*-hydroxybenzoic acid, vanillic acid, caffeic acid, and coumaric acid, are indicated as compounds with anti-cancer activity [34,36]. The correlation analysis that was carried out in this study showed that the ability of the tested extracts to inhibit the proliferation and viability of the tested cell lines was mainly related to the presence of ferulic, sinapic, and salicylic acids in the tested extracts (Table 3). A study conducted by Elansary et al. [36] partially confirmed our observation, which suggested that ferulic acid, i.e., the major phenolic compound found in *Catalpa speciosa* bark extract, is highly associated with anticancer activity.

## 5. Conclusions

In conclusion, dried lovage (control and elicited with jasmonic acid and yeast extract) has significant advantages in terms of health-promoting properties, which is shown in the study of the potential bioavailability of phenolic acids and the pro-health properties of the bioavailable fraction of phenolic acids. The elicitation (especially with JA) resulted in a statistically significant increase in the content of most of the analyzed phenolic acids, which, in some cases, resulted in an increase in bioactive properties (especially antioxidant and antiproliferative activity). The content of *p*-hydroxybenzoic acid was correlated with the antioxidant activity and with the ability of the studied extracts to inhibit LOX and COX2, while the antiproliferative activity seems to be mainly related to ferulic, sinapic, and salicylic acids. The present research proves that the drying of elicited herbs does not deteriorate their health-promoting properties and the use of elicitation to modify the biological activity of herbal plants does not exclude the use of such plants in a dried form, which is a valuable indication for the food industry.

## Figures and Tables

**Table 1:** Qualitative and quantitative analysis of phenolic acids in the ethanolic, buffer (PBS (phosphate buffered saline)), and gastrointestinally digested (GD) extracts from control and elicited dried lovage leaves.

Sample	Compounds [mg/g DW]							
	Protocatechuic Acid	*p*-hydroxybenzoic Acid	Caffeic Acid	Syringic Acid	Vanillic Acid	Ferulic Acid	Sinapic Acid	Salicylic Acid
**Freeze-Dried Samples**								
Ethanolic extracts								
CL-E	159.59 ± 0.42 dC	nd	177.32 ± 0.98 eE	15.34 ± 0.02 aA	7.71 ± 0.02 bAB	10.40 ± 0.06 aA	102.14 ± 0.52 aC	492.75 ± 1.37 aC
JAL-E	413.56 ± 0.78 iE	nd	440.86 ± 1.43 jG	38.55 ± 0.23 eC	nd	15.84 ± 0.01 cC	260.91 ± 0.93 hF	1149.10 ± 6.23 hF
YEL-E	264.84 ± 0.41 hD	nd	312.03 ± 0.62 iF	22.02 ± 0.12 cB	6.19 ± 0.03 aA	13.52 ± 0.05 bB	232.78 ± 0.49 fgE	1036.70 ± 8.10 gE
PBS extracts								
CL-P	nd	15.65 ± 0.06 cdC	10.22 ± 0.01 aA	nd	12.58 ± 0.49 cD	nd	84.73 ± 4.20 cB	nd
JAL-P	9.56 ± 0.08 aA	13.64 ± 1.92 cB	12.45 ± 0.16 abB	nd	10.42 ± 0.13 bC	nd	110.77 ± 7.57 dC	33.65 ± 0.44 aA
YEL-P	nd	9.45 ± 0.15 aA	nd	nd	8.42 ± 1.58 aB	nd	61.44 ± 3.21 bA	nd
GD extracts								
CL-GD	31.27 ± 9.91 abcB	150.22 ± 12.05 bD	18.73 ± 4.47 abcC	14.13 ± 3.67 abcA	16.88 ± 0.76 bcE	nd	99.14 ± 7.89 abBC	185.15 ± 34.42 abB
JAL-GD	126.67 ± 29.42 eC	273.40 ± 22.48 deE	138.38 ± 2.19 eD	54.39 ± 7.79 eD	26.63 ± 3.12 deF	nd	303.54 ± 40.57 fG	693.13 ± 74.54 eD
YEL-GD	nd	290.60 ± 0.21 eF	nd	nd	18.40 ± 0.02 cE	nd	188.43 ± 1.05 deD	nd
**Microwave-Dried Samples**								
Ethanolic extracts								
CM-E	111.43 ± 0.34 bD	nd	148.59 ± 3.46 dD	34.12 ± 0.20 dC	10.68 ± 0.07 cA	20.82 ± 0.06 dA	186.76 ± 2.82 bcD	654.54 ± 16.19 cF
JAM-E	235.26 ± 6.39 gE	nd	257.94 ± 7.48 hF	52.38 ± 0.69 hE	15.91 ± 0.57 fC	33.94 ± 2.17 hC	210.74 ± 8.11 dE	968.39 ± 74.54 fH
YEM-E	130.39 ± 6.43 cD	nd	173.08 ± 5.51 eE	44.66 ± 1.46 fD	12.32 ± 0.12 dB	25.96 ± 3.38 gB	223.97 ± 13.16 efE	781.96 ± 55.85 eG
PBS extracts								
CM-P	46.45 ± 0.21 dB	nd	58.92 ± 0.33 dC	15.17 ± 0.11 cB	15.93 ± 0.14 dC	nd	108.55 ± 0.56 dB	146.09 ± 1.19 eB
JAM-P	240.68 ± 0.65 hE	nd	290.26 ± 0.61 iG	71.93 ± 0.19 hF	30.65 ± 0.08 fD	nd	190.61 ± 0.12 fD	557.83 ± 1.30 hE
YEM-P	22.88 ± 0.16 cA	37.42 ± 0.25 gA	18.80 ± 0.06 cA	10.01 ± 0.04 bA	12.04 ± 0.25 cB	nd	86.61 ± 0.22 cA	117.31 ± 0.23 cA
GD extracts								
CM-GD	74.52 ± 16.44 dC	210.29 ± 19.73 cC	36.61 ± 6.50 dB	29.62 ± 5.29 dC	16.72 ± 1.60 bcC	nd	159.91 ± 24.89 cdC	409.11 ± 91.18 cD
JAM-GD	253.35 ± 27.51 fE	272.01 ± 2.78 deD	153.74 ± 14.10 fD	102.83 ± 10.04 fG	30.36 ± 1.09 eD	nd	278.95 ± 1.77 fF	1019.41 ± 23.70 fH
YEM-GD	50.05 ± 16.66 abcdC	109.75 ± 26.71 aB	27.97 ± 7.26 bcdAB	20.18 ± 5.99 abcdBC	14.05 ± 1.92 bC	nd	93.20 ± 26.45 abAB	197.93 ± 84.82 abC
**Traditionally Dried Samples**								
Ethanolic extracts								
CT-E	67.93 ± 0.25 aB	61.63 ± 0.06 dB	73.88 ± 0.33 bE	20.54 ± 0.30 bC	16.35 ± 0.10 fC	16.67 ± 0.05 cA	183.75 ± 0.29 bD	544.54 ± 0.92 bE
JAT-E	109.24 ± 0.61 bC	97.05 ± 0.19 fC	119.42 ± 0.82 cG	46.42 ± 0.04 gE	18.73 ± 0.08 iE	21.49 ± 0.02 deB	261.88 ± 0.32 hF	770.86 ± 0.55 eF
YET-E	62.99 ± 0.45 aB	63.64 ± 0.14 eB	65.42 ± 0.14 aD	19.50 ± 0.06 bC	16.03 ± 0.12 fC	16.48 ± 0.01 cA	194.22 ± 0.07 bcD	536.23 ± 0.98 abE
PBS extracts								
CT-P	20.10 ± 0.22 cA	25.47 ± 0.12 eA	20.55 ± 0.13 cB	9.82 ± 0.07 bB	9.41 ± 0.13 abB	nd	48.32 ± 0.03 aA	74.72 ± 0.60 bA
JAT-P	106.57 ± 0.33 fC	99.79 ± 0.06 hC	96.23 ± 0.15 gF	48.25 ± 0.10 gF	29.18 ± 0.21 eG	nd	228.75 ± 0.03 gE	438.91 ± 1.48 gD
YET-P	15.59 ± 0.46 bA	27.46 ± 0.09 fA	15.24 ± 0.11 bA	6.73 ± 0.04 aA	8.61 ± 0.04 aA	nd	63.59 ± 0.20 bB	74.10 ± 0.08 bA
GD extracts								
CT-GD	21.49 ± 3.90 abA	273.23 ± 16.57 deD	16.14 ± 0.40 abA	10.21 ± 0.02 abB	18.75 ± 1.50 c	nd	171.10 ± 33.59 dD	298.74 ± 49.77 bcC
JAT-GD	62.93 ± 0.46 cdB	296.99 ± 1.38 eE	36.94 ± 0.27 dC	25.10 ± 0.19 cdD	26.18 ± 0.14 dF	nd	226.70 ± 0.89 eE	546.00 ± 4.45 dE
YET-GD	26.81 ± 0.47 abA	174.08 ± 0.18 bD	17.81 ± 0.42 bAB	11.74 ± 0.10 abB	17.41 ± 0.02 bcD	nd	115.97 ± 0.22 bcC	189.18 ± 0.10 abB
**Convectionally Dried Samples**								
Ethanolic extracts								
CC-E	186.76 ± 0.52 fE	27.05 ± 0.16 aC	202.31 ± 0.66 gG	63.05 ± 0.07 jH	17.20 ± 0.17 gE	24.19 ± 0.10 fgC	197.18 ± 0.63 cG	812.49 ± 1.04 eH
JAC-E	171.51 ± 0.29 eE	40.76 ± 0.16 bD	188.60 ± 0.38 fF	58.11 ± 0.26 iG	14.79 ± 0.09 eC	23.29 ± 0.03 efB	215.38 ± 0.19 deH	775.73 ± 0.59 eG
YEC-E	129.45 ± 0.14 cD	42.06 ± 0.02 cD	153.35 ± 0.29 dE	51.98 ± 0.11 hF	17.71 ± 0.05 hE	22.52 ± 0.01 defA	236.07 ± 0.20 gI	712.59 ± 0.26 dF
PBS extracts								
CC-P	65.00 ± 0.59 eC	11.36 ± 0.03 bA	68.90 ± 0.50 fD	26.33 ± 0.13 eD	11.90 ± 0.04 cB	nd	57.19 ± 0.04 bB	127.35 ± 0.72 dB
JAC-P	63.31 ± 1.37 eBC	14.55 ± 0.07 cdB	65.20 ± 1.16 eD	23.59 ± 0.23 dC	9.60 ± 0.06 abA	nd	64.71 ± 1.26 bC	160.75 ± 1.45 fC
YEC-P	126.44 ± 2.94 gD	nd	157.84 ± 3.83 hE	42.82 ± 0.66 fE	16.89 ± 0.01 dD	nd	174.35 ± 5.69 eE	440.20 ± 8.90 gE
GD extracts								
CC-GD	52.58 ± 11.53 bcdB	162.02 ± 21.63 bF	32.07 ± 3.97 cdC	24.36 ± 3.61 cdCD	17.16 ± 2.83 bcE	nd	121.51 ± 11.87 bcD	279.64 ± 55.88 bD
JAC-GD	50.07 ± 0.42 abcdB	240.28 ± 0.27 cdG	23.97 ± 0.06 abcdB	20.58 ± 0.13 bcdB	24.69 ± 0.05 dF	nd	187.68 ± 0.13 deF	287.81 ± 1.49 bcD
YEC-GD	17.09 ± 0.05 aA	79.95 ± 0.08 aE	12.92 ± 0.09 aA	8.65 ± 0.03 aA	10.17 ± 0.02 aA	nd	55.19 ± 0.14 aA	86.44 ± 0.19 aA

Abbreviations: C, control; JA, plants elicited with 10 µM of jasmonic acid; YE, plants elicited with 0.1% yeast extract; CL-E, JAL-E, YEL-E —ethanolic extracts from freeze-dried samples; CL-P, JAL-P, YEL-P—PBS extracts from freeze-dried samples; CL-GD, JAL-GD, YEL-GD gastrointestinally digested extracts from freeze-dried samples; CM-E, JAM-E, YEM-E— ethanolic extracts from microwave-dried samples; CM-P, JAM-P, YEM-P -PBS extracts from microwave-dried samples; CM-GD, JAM-GD, YEM-GD—gastrointestinally digested extracts from microwave-dried samples; CT-E, JAT-E, YET-E— ethanolic extracts from traditionally dried samples; CT-P, JAT-P, YET-P –PBS extracts from traditionally dried samples; CT-GD, JAT-GD, YET-GD—gastrointestinally digested extracts from traditionally dried samples; CC-E, JAC-E, YEC-E— ethanolic extracts from convectionally dried samples; CC-P, JAC-P, YEC-P –PBS extracts from convectionally dried samples; CC-GD, JAC-GD, YEC-GD—gastrointestinally digested extracts from convectionally dried samples. The means (±SD) in the columns followed by different lowercase letters are significantly different for the same type of extract but different drying methods. Means (±SD) in the columns followed by different capital letters are significantly different for the same of drying methods but different extracts (*p* ≤ 0.05).

**Table 2:** The effect of elicitation and drying methods on the antioxidant and potentially anti-inflammatory properties of the ethanolic, buffer (PBS (phosphate buffered saline)), and gastrointestinally digested (GD) extracts from dried lovage leaves.

Extract Type	Sample	Antioxidant Activities			Potential Anti-Inflammatory Activities	
		ABTS [µMTrolox/g DW]	RP [mg Trolox/g DW]	CHP [mg EDTA/g DW]	LOX Inhibition EC_50_ [mg DW/mL]	COX2 Inhibition EC_50_ [mg DW/mL]
**Freeze-Dried Samples**						
Ethanolic extracts	CL-E	1.87 ± 0.21 bdAB	19.97 ± 5.98 bcB	3.18 ± 0.35 gB	0.115 ± 0.004 dD	0.113 ± 0.0004 deDE
	JAL-E	1.99 ± 0.10 dB	18.35 ± 5.73 bcB	3.19 ± 0.40 gB	0.145 ± 0.004 eE	0.021 ± 0.000 aA
	YEL-E	1.53 ± 0.17 bcA	7.90 ± 2.94 aA	2.28 ± 0.32 cefA	0.208 ± 0.015 fDE	0.048 ± 0.0001 bB
PBS extracts	CL-P	3.98 ± 0.79 cdeC	22.03 ± 4.52 dB	6.50 ± 0.34 abC	0.044 ± 0.001 aA	0.075 ± 0.004 cC
	JAL-P	4.25 ± 0.62 eC	19.64 ± 0.72 bcdB	5.95 ± 0.63 aC	0.068 ± 0.003 cC	0.122 ± 0.006 eE
	YEL-P	4.17 ± 0.98 deC	15.73 ± 1.32 abcdAB	6.68 ± 0.50 abC	0.063 ± 0.0005 cC	0.103 ± 0.001 dD
GD extracts	CL-GD	6.82 ± 0.36 eE	98.81 ± 4.83 bcdC	14.25 ± 0.49 bD	0.059 ± 0.004 abcABC	0.019 ± 0.0001 aA
	JAL-GD	6.40 ± 0.41 deDE	115.93 ± 6.04 cdeC	14.48 ± 0.35 bD	0.061 ± 0.002 bcBC	0.038 ± 0.003 bB
	YEL-GD	5.71 ± 0.32 dD	108.29 ± 4.36 cdC	14.77 ± 0.14 bD	0.047 ± 0.004 abAB	0.013 ± 0.0005 aA
**Microwave-Dried Samples**						
Ethanolic extracts	CM-E	0.92 ± 0.16 aA	14.17 ± 0.93 abA	2.41 ± 0.17 efA	0.296 ± 0.032 eE	0.209 ± 0.011 fF
	JAM-E	1.46 ± 0.16 bB	17.88 ± 1.25 bcA	0.98 ± 0.12 aA	0.164 ± 0.008 cC	0.147 ± 0.012 dD
	YEM-E	0.95 ± 0.12 aAB	9.42 ± 0.46 aA	2.25 ± 0.16 cefA	0.224 ± 0.013 dD	0.171 ± 0.0003 eE
PBS extracts	CM-P	3.05 ± 0.95 bcdC	20.90 ± 1.13 cdA	6.92 ± 0.71 abB	0.072 ± 0.011 abAB	0.044 ± 0.0004 bB
	JAM-P	4.59 ± 0.57 eD	20.90 ± 8.03 cdA	7.30 ± 1.15 bB	0.097 ± 0.002 bB	0.058 ± 0.002 bcBC
	YEM-P	4.66 ± 0.24 eD	20.31 ± 6.81 bcdA	7.03 ± 0.33 abB	0.072 ± 0.0001 abAB	0.061 ± 0.003 cC
GD extracts	CM-GD	4.18 ± 0.22 bcD	115.46 ± 4.47 cdeC	13.97 ± 1.29 bD	0.068 ± 0.004 abAB	0.012 ± 0.0001 aA
	JAM-GD	4.06 ± 0.37 bcD	131.72 ± 6.39 eD	12.11 ± 1.86 aC	0.059 ± 0.002 aA	0.013 ± 0.0006 aA
	YEM-GD	4.12 ± 0.15 bcD	101.66 ± 9.20 bcdB	14.03 ± 0.53 bD	0.069 ± 0.001 abAB	0.013 ± 0.0002 aA
**Traditionally Dried Samples**						
Ethanolic extracts	CT-E	1.40 ± 0.17 bA	25.98 ± 2.06 cC	1.62 ± 0.40 abcA	0.221 ± 0.028 bB	0.051 ± 0.001 dD
	JAT-E	1.82 ± 0.16 cdAB	20.70 ± 4.23 bcBC	2.32 ± 0.14 cefAB	0.198 ± 0.0005 bB	0.024 ± 0.001 bB
	YET-E	2.05 ± 0.11 cBC	15.20 ± 2.01 abAB	2.75 ± 0.49 fgB	0.205 ± 0.002 bB	0.031 ± 0.001 cC
PBS extracts	CT-P	1.89 ± 0.14 aB	8.83 ± 3.93 aA	7.29 ± 0.35 bC	0.051 ± 0.008 aA	0.055 ± 0.004 eD
	JAT-P	4.53 ± 0.34 eF	11.88 ± 3.56 abcdA	7.08 ± 0.12 abC	0.049 ± 0.004 aA	0.057 ± 0.002 eD
	YET-P	3.00 ± 0.20 abcD	10.15 ± 2.06 abA	6.72 ± 0.19 abC	0.060 ± 0.0001 aA	0.055 ± 0.001 deD
GD extracts	CT-GD	4.64 ± 0.61 cF	37.69 ± 3.43 aD	14.10 ± 0.91 bD	0.049 ± 0.002 aA	0.013 ± 0.0007 aA
	JAT-GD	4.03 ± 0.25 bcF	42.60 ± 2.51 aD	13.69 ± 0.84 abD	0.043 ± 0.003 aA	0.019 ± 0.002 abAB
	YET-GD	3.70 ± 0.24 bE	41.34 ± 3.84 aD	13.44 ± 0.69 abD	0.051 ± 0.002 aA	0.030 ± 0.001 cC
**Convectionally Dried Samples**						
Ethanolic extracts	CC-E	1.99 ± 0.20 dAB	25.98 ± 2.06 cA	2.50 ± 0.30 efgA	0.175 ± 0.011 cC	0.083 ± 0.0003 cC
	JAC-E	2.00 ± 0.15 dAB	20.70 ± 4.23 bcA	1.86 ± 0.38 bceA	0.179 ± 0.001 cC	0.167 ± 0.005 eE
	YEC-E	1.46 ± 0.09 bA	15.20 ± 2.01 abA	1.36 ± 0.84 abA	0.212 ± 0.003 dD	0.176 ± 0.004 eE
PBS extracts	CC-P	2.43 ± 0.50 abBC	11.02 ± 3.60 abcA	6.49 ± 1.14 abB	0.146 ± 0.001 bB	0.099 ± 0.010 dD
	JAC-P	2.40 ± 0.44 abBC	13.14 ± 4.19 abcdA	6.20 ± 0.73 abB	0.211 ± 0.012 dD	0.091 ± 0.001 cdD
	YEC-P	3.97 ± 0.50 cdeD	13.60 ± 4.43 abcdA	7.13 ± 0.27 abB	0.22 ± 0.001 dD	0.093 ± 0.0003 cdD
GD extracts	CC-GD	3.93 ± 0.65 bcD	98.54 ± 24.95 bcB	13.81 ± 0.32 bC	0.131 ± 0.010 abAB	0.03 ± 0.002 bB
	JAC-GD	6.23 ± 0.35 deE	80.82 ± 0.34 bB	13.51 ± 0.43 abC	0.122 ± 0.006 aA	0.018 ± 0.001 abAB
	YEC-GD	2.84 ± 0.50 aC	121.30 ± 11.08 deC	14.28 ± 0.56 bC	0.117 ± 0.005 aA	0.016 ± 0.001 aA

Abbreviations: C, control; JA, plants elicited with 10 µM of jasmonic acid; YE, plants elicited with 0.1% yeast extract; CL-E, JAL-E, YEL-E —ethanolic extracts from freeze-dried samples; CL-P, JAL-P, YEL-P—PBS extracts from freeze-dried samples; CL-GD, JAL-GD, YEL-GD gastrointestinally digested extracts from freeze-dried samples; CM-E, JAM-E, YEM-E— ethanolic extracts from microwave-dried samples; CM-P, JAM-P, YEM-P -PBS extracts from microwave-dried samples; CM-GD, JAM-GD, YEM-GD—gastrointestinally digested extracts from microwave-dried samples; CT-E, JAT-E, YET-E— ethanolic extracts from traditionally dried samples; CT-P, JAT-P, YET-P –PBS extracts from traditionally dried samples; CT-GD, JAT-GD, YET-GD—gastrointestinally digested extracts from traditionally dried samples; CC-E, JAC-E, YEC-E—ethanolic extracts from convectionally dried samples; CC-P, JAC-P, YEC-P –PBS extracts from convectionally dried samples; CC-GD, JAC-GD, YEC-GD—gastrointestinally digested extracts from convectionally dried samples. Means (±SD) in the columns followed by different lowercase letters are significantly different for the same type of extract but different drying methods. The means (±SD) in the columns followed by different capital letters are significantly different for the same of drying methods, but different extracts (*p* ≤ 0.05).

**Table 3:** Pearson’s correlation coefficients for phenolic acid content and in vitro bioactivities.

	Protocatechuic Acid	p-hydroxybenzoic Acid	Caffeic Acid	Syringic Acid	Vanillic Acid	Ferulic Acid	Sinapic Acid	Salicylic Acid
ABTS	−0.32	0.63	−040	−0.11	0.50	−0.69	−0.04	−0.40
RP	−0.14	0.72	−0.29	0.08	0.36	−0.45	0.08	−0.10
CHP	−0.43	0.76	−0.54	−0.22	0.38	−0.78	−0.19	−0.47
LOX inhibition *	0.37	−0.48	0.47	0.29	−0.26	0.66	0.28	0.50
COX2 inhibition *	0.14	−0.60	0.27	0.18	−0.27	0.57	0.01	0.19
inhibition of proliferation of NCI-N87 cell line	0.13	0.27	0.14	0.06	0.11	0.45	0.43	0.40
inhibition of viability of NCI-N87 cell line	0.34	0.12	0.38	0.16	−0.01	0.62	0.54	0.59
inhibition of proliferation of VCaP cell line	0.07	0.37	0.09	0.08	0.18	0.36	0.47	0.33
inhibition of viability of VCaP cell line	0.18	0.27	0.21	0.14	0.13	0.52	0.52	0.45
inhibition of proliferation of HPrEC cell line	0.36	0.11	0.38	0.02	−0.14	0.39	0.41	0.49
inhibition of viability of HPrEC cell line	0.37	0.14	0.40	0.21	−0.02	0.63	0.56	0.62

* a negative correlation between the phenolic acid content and the EC50 values indicates a positive relationship between phenolic content and LOX/COX2 inhibition (low EC50 values indicate high ability to inhibit LOX/COX2).

**Table 4:** In vitro effects of the highest concentration of the analyzed extracts after 72-h treatment on the cancer gastric epithelial NCI-N87 (ATCC^®^ CRL5822 ™), prostate cancer VCaP (ATCC^®^ CRL-2876 ™), and healthy prostate epithelial HPrEC cell lines (ATCC^®^ PCS-440-010™).

Sample	NCI-N87 (ATCC^®^ CRL5822 ™)			VCaP—(ATCC^®^ CRL-2876 ™)			HPrEC (ATCC^®^ PCS-440-010 ™)		
	Proliferation	Viability	Cell Cycle	Proliferation	Viability	Cell Cycle	Proliferation	Viability	Cell Cycle
**Freeze-Dried Samples**									
Ethanolic extracts									
CL-E	↓↓	↓↓↓	↑G1↓G2	↓	↓↓	n.c.	↓↓↓↓	↓↓↓	↑S↓G1
JAL-E	↓↓	↓↓↓	↑G1↓G2	↓	↓↓	↑G2	↓↓↓↓	↓↓↓	↑S↓G1
YEL-E	↓	↓↓↓	↑G1↓S	↓	↓	↑G2	↓↓↓↓	↓↓↓	↑S↓G1
PBS extracts									
CL-P	n.c.	n.c.	n.c.	n.c.	n.c.	n.c.	n.c.	n.c.	n.c.
JAL-P	n.c.	n.c.	n.c.	n.c.	n.c.	n.c.	n.c.	n.c.	n.c.
YEL-P	n.c.	n.c.	n.c.	n.c.	n.c.	n.c.	n.c.	n.c.	n.c.
GD extracts									
CL-GD	↓	↓↓	n.c.	↓	↓	↑G1↓S	n.c.	↓	↑S↓G1,G2
JAL-GD	↓	↓↓	n.c.	↓↓	↓↓	↑G1↓S,G2	↓	↓↓	↑S↓G2
YEL-GD	↓	↓	n.c.	↓↓	↓↓	↑G1↓S,G2	↓	↓↓	↑S
**Microwave-Dried Samples**									
Ethanolic extracts									
CM-E	↓↓	↓↓↓	↑S↓G2	↓↓↓	↓↓	↑G1↓S	↓	↓↓↓	n.c.
JAM-E	↓↓	↓↓↓	↑G1	↓	↓↓	↑G1↓S	↓	↓↓↓	n.c.
YEM-E	↓↓	↓↓↓	n.c.	n.c.	↓	↑G1↓S	↓	↓↓↓	↑G2
PBS extracts									
CM-P	n.c.	n.c.	n.c.	n.c.	n.c.	n.c.	n.c.	n.c.	n.c.
JAM-P	n.c.	n.c.	n.c.	n.c.	n.c.	n.c.	n.c.	n.c.	n.c.
YEM-P	n.c.	n.c.	n.c.	n.c.	n.c.	n.c.	n.c.	n.c.	n.c.
GD extracts									
CM-GD	n.c.	n.c.	↑S↓G1	n.c.	n.c.	↑G1	n.c.	n.c.	↑G2
JAM-GD	n.c.	n.c.	↑S↓G1	↑	n.c.	n.c.	n.c.	↓	↑G2
YEM-GD	↑	n.c.	n.c.	n.c.	n.c.	↑G1	n.c.	n.c.	↑G2
**Traditionally Dried Samples**									
Ethanolic extracts									
CT-E	↓↓↓	↓↓↓	↓G2	↓↓	↓↓↓	↓G2	↓↓↓	↓↓↓	↑G2↓G1
JAT-E	↓↓↓	↓↓↓	↓G2	↓↓	↓↓↓	n.c.	↓↓↓	↓↓↓	↑G2↓G1
YET-E	↓↓↓	↓↓↓	↑G2	↓↓	↓↓↓	↑G1↓G2	↓↓↓	↓↓↓	↑G2↓G1
PBS extracts									
CT-P	n.c.	n.c.	n.c.	n.c.	n.c.	n.c.	n.c.	n.c.	n.c.
JAT-P	n.c.	n.c.	n.c.	n.c.	n.c.	n.c.	n.c.	n.c.	n.c.
YET-P	n.c.	n.c.	n.c.	n.c.	n.c.	n.c.	n.c.	n.c.	n.c.
GD extracts									
CT-GD	↓↓↓	↓↓↓	n.c.	↓↓	↓↓↓	↑S	↓↓↓	↓↓↓	↓S
JAT-GD	↓↓↓	↓↓↓	↓G1	↓↓	↓↓↓	↑S	↓↓↓	↓↓↓	↓S
YET-GD	↓↓↓	↓↓↓	↓G1	↓↓	↓↓↓	↓G2	↓↓↓	↓↓↓	↓S
**Convectionally Dried Samples**									
Ethanolic extracts									
CC-E	↓↓	↓↓↓	n.c.	↓↓	↓↓↓	↑G1↓S,G2	↓↓	↓↓↓	↑S↓G2
JAC-E	↓↓	↓↓↓	n.c.	↓↓	↓↓↓	↑G1↓S,G2	↓↓↓	↓↓↓	↑S↓G2
YEC-E	↓↓	↓↓↓	n.c.	↓↓	↓↓↓	↑G1↓S	↓↓	↓↓↓	↑S↓G2
PBS extracts									
CC-P	n.c.	n.c.	n.c.	n.c.	n.c.	n.c.	n.c.	n.c.	n.c.
JAC-P	n.c.	n.c.	n.c.	n.c.	n.c.	n.c.	n.c.	n.c.	n.c.
YEC-P	n.c.	n.c.	n.c.	n.c.	n.c.	n.c.	n.c.	n.c.	n.c.
GD extracts									
CC-GD	↓↓↓	↓↓	↑S	↓↓	↓	n.c.	↓↓↓	↓↓↓	↑G2↓S
JAC-GD	↓↓↓	↓↓↓	↑S	↓↓	↓↓	n.c.	↓↓	↓	↑G2
YEC-GD	n.c.	n.c.	↑S	↑↑	n.c.	↑G1	↓	↓	↑G1↓S

Abbreviations: C, control; JA, plants elicited with 10 µM of jasmonic acid; YE, plants elicited with 0.1% yeast extract; CL-E, JAL-E, YEL-E —ethanolic extracts from freeze-dried samples; CL-P, JAL-P, YEL-P—PBS extracts from freeze-dried samples; CL-GD, JAL-GD, YEL-GD gastrointestinally digested extracts from freeze-dried samples; CM-E, JAM-E, YEM-E— ethanolic extracts from microwave-dried samples; CM-P, JAM-P, YEM-P -PBS extracts from microwave-dried samples; CM-GD, JAM-GD, YEM-GD—gastrointestinally digested extracts from microwave-dried samples; CT-E, JAT-E, YET-E— ethanolic extracts from traditionally dried samples; CT-P, JAT-P, YET-P –PBS extracts from traditionally dried samples; CT-GD, JAT-GD, YET-GD—gastrointestinally digested extracts from traditionally dried samples; CC-E, JAC-E, YEC-E— ethanolic extracts from convectionally dried samples; CC-P, JAC-P, YEC-P –PBS extracts from convectionally dried samples; CC-GD, JAC-GD, YEC-GD—gastrointestinally digested extracts from convectionally dried samples. n.c.—no change; ↓—decrease; ↑—increase; G1, S, G2—cell cycle phases; number of ↓ or ↑—level of changes: ↓↓↓↓—the highest, ↓—the lowest.

## Data Availability

Not applicable.

## Supplementary Materials

The following are available online at https://www.mdpi.com/article/10.3390/antiox10050662/s1.
