# Supplementary material for: Effects of Drying Methods on Antioxidant, Anti-Inflammatory, and Anticancer Potentials of Phenolic Acids in Lovage Elicited by Jasmonic Acid and Yeast Extract
Source: Antioxidants (Basel). 2021 Apr 24;10(5):662. doi: 10.3390/antiox10050662 (PMC8146002; doi:10.3390/antiox10050662)
Supplement: Supplementary file 1 [file antioxidants-10-00662-s001.zip › antioxidants-1179733-supplementary.pdf]

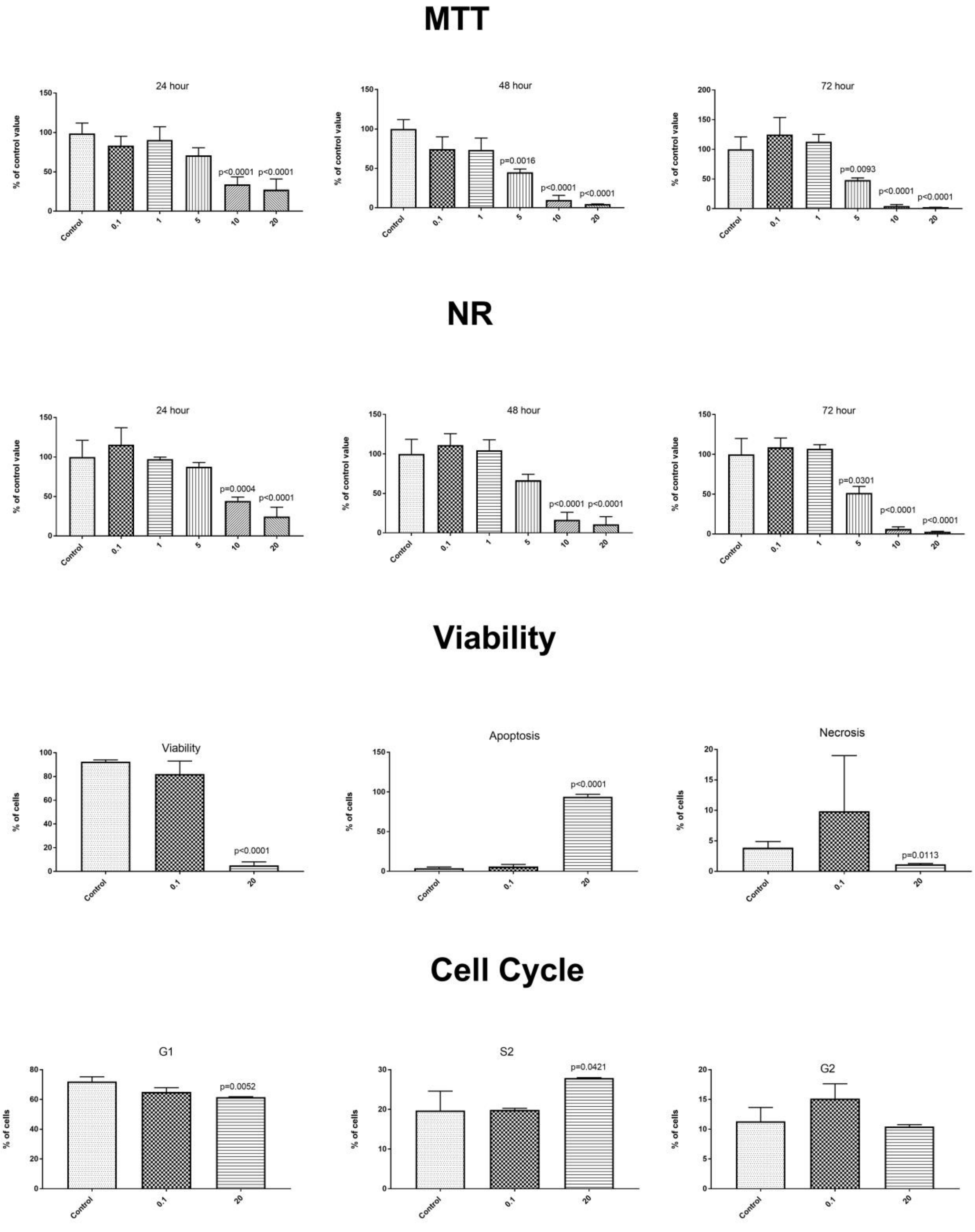

Figure S1. Impact of CL-E (ethanolic extract of control freeze-dried sample) on cytotoxicity (MTT and NR method), type of cell death (Annex V and PI test) and individual phases of the cell cycle of the healthy prostate epithelial cells (HPrEC ATCC® PCS-440-010 ™ cell line).

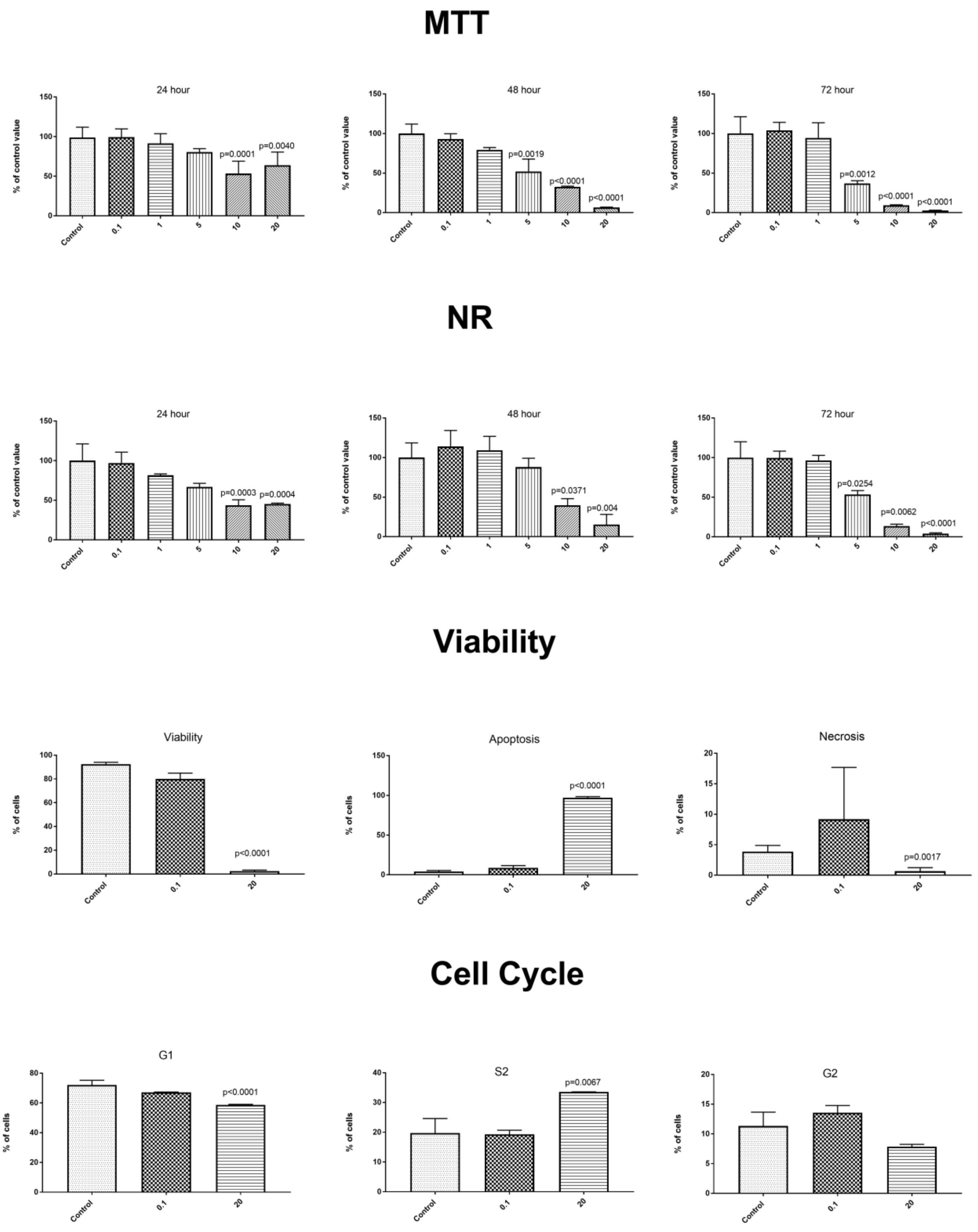

Figure S2. Impact of JAL-E (ethanolic extract of freeze-dried sample from plants elicited with 10 µM of jasmonic acid) on cytotoxicity (MTT and NR method), type of cell death (Annex V and PI test) and individual phases of the cell cycle of the healthy prostate epithelial cells (HPrEC ATCC® PCS-440-010 ™ cell line).

MTT

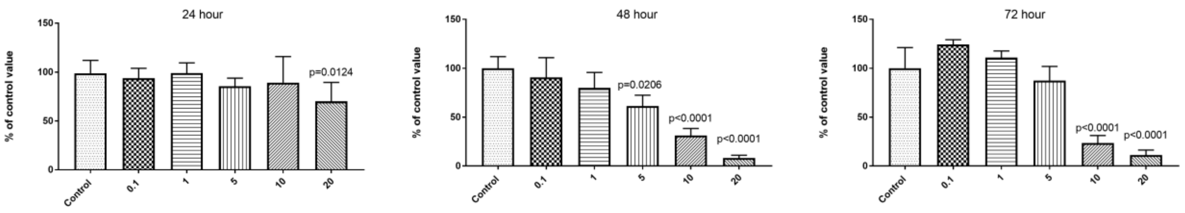

NR

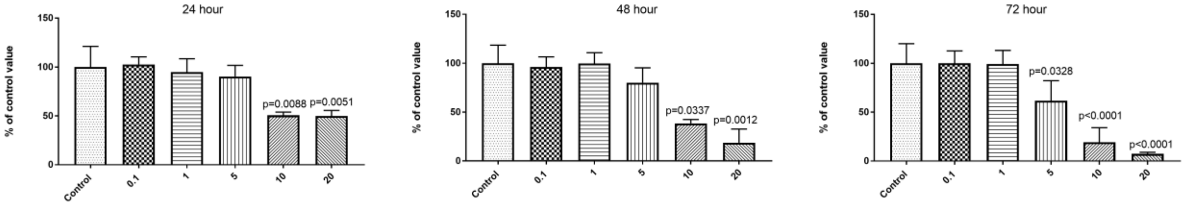

Viability

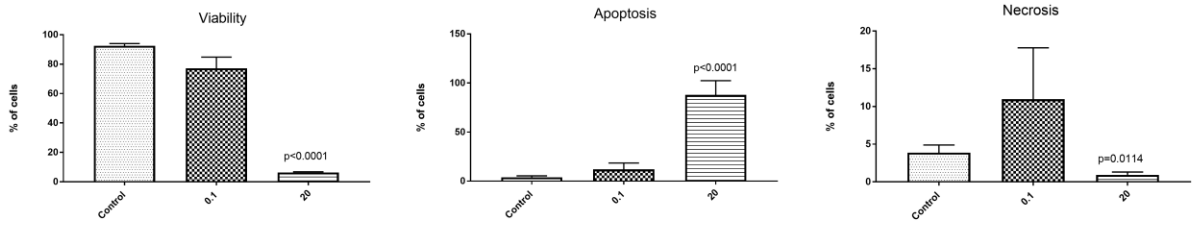

Cell Cycle

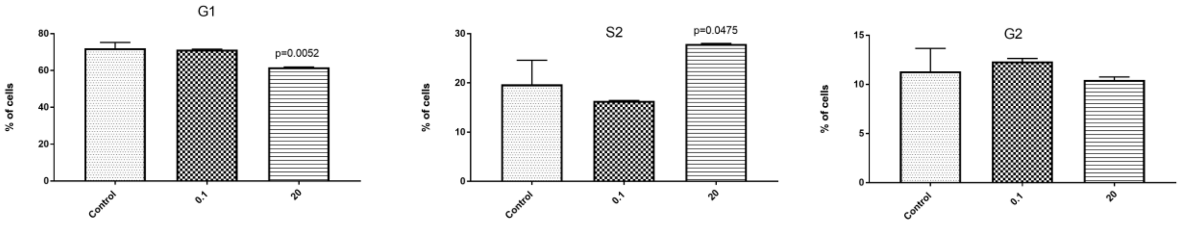

Figure S3. Impact of YEL-E (ethanolic extract of freeze-dried sample from plants elicited with 0.1% yeast extract) on cytotoxicity (MTT and NR method), type of cell death (Annex V and PI test) and individual phases of the cell cycle of the healthy prostate epithelial cells (HPrEC ATCC® PCS-440-010 ™ cell line).

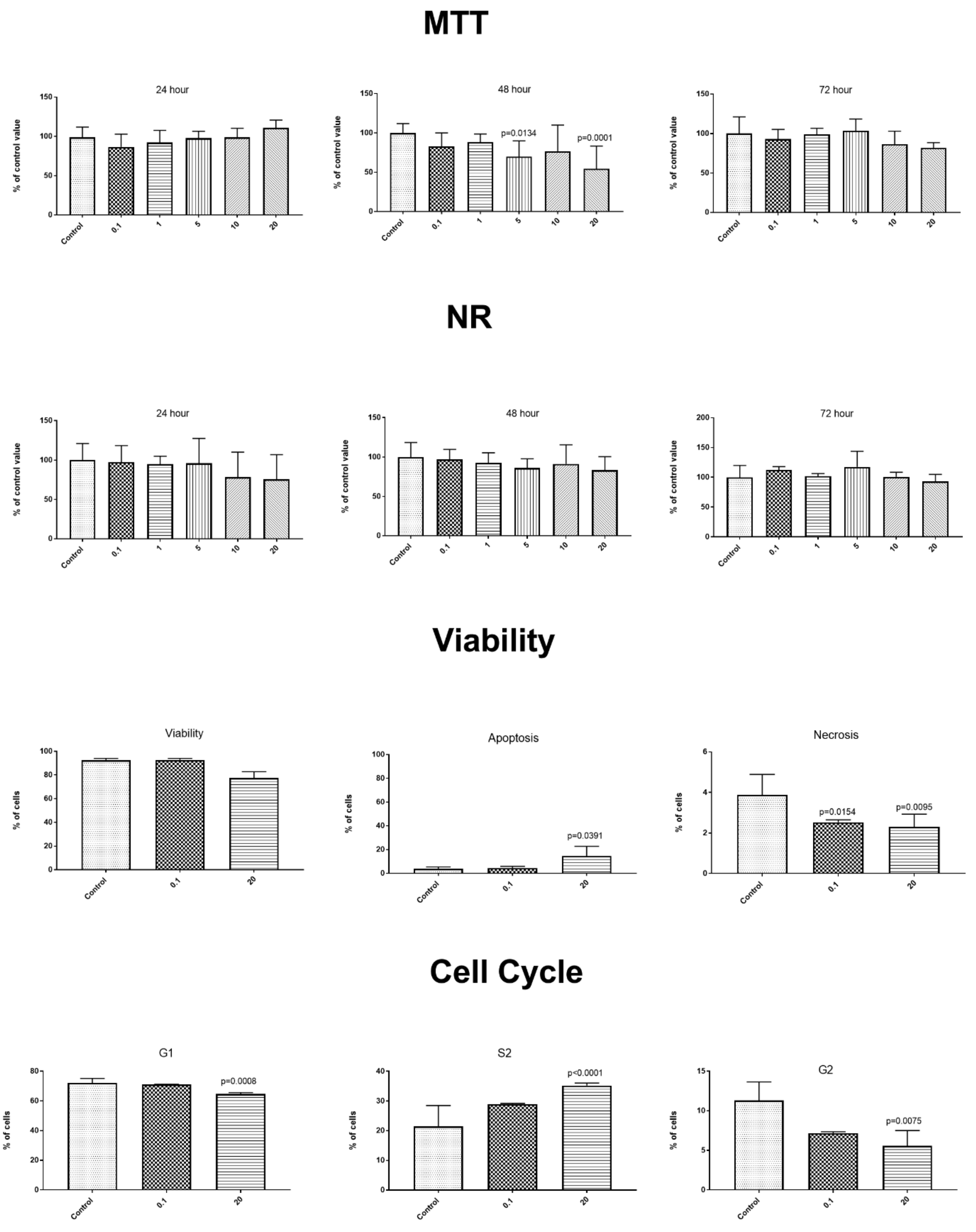

Figure S4. Impact of CL-GD extract (sample after the *in vitro* digestion of control freeze-dried sample) on cytotoxicity (MTT and NR method), type of cell death (Annex V and PI test) and individual phases of the cell cycle of the healthy prostate epithelial cells (HPrEC ATCC® PCS-440-010 ™ cell line).

MTT

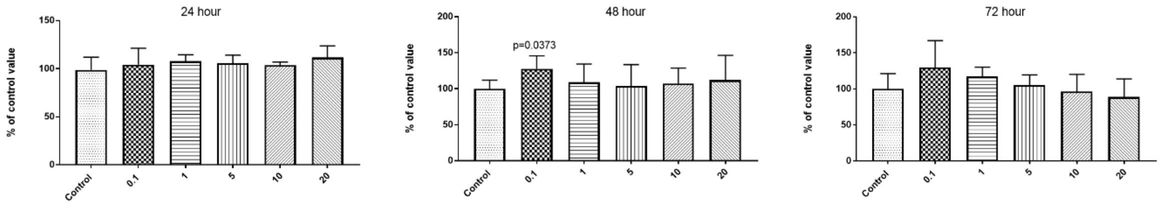

NR

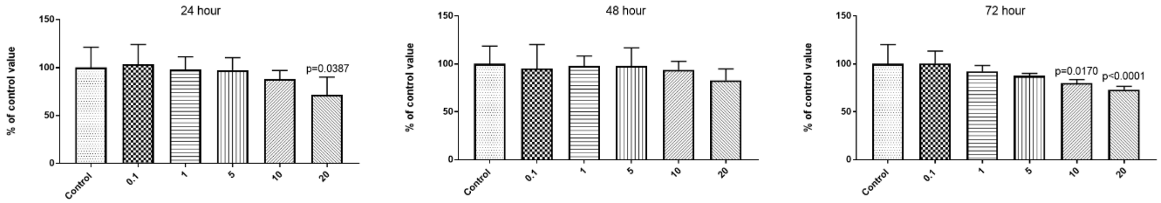

Viability

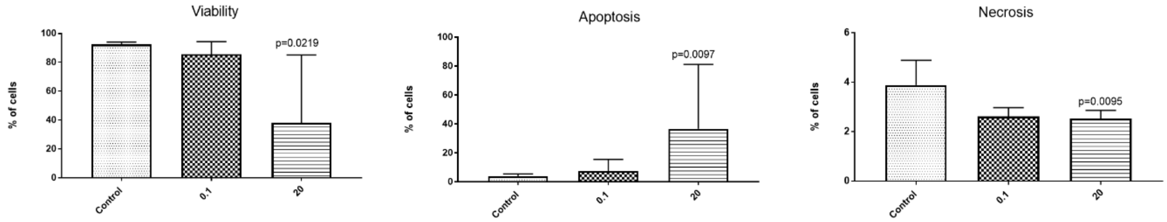

Cell Cycle

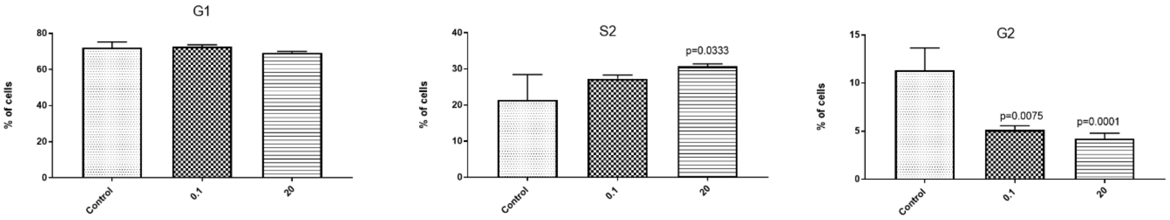

Figure S5. Impact of JAL-GD (sample after the *in vitro* digestion of freeze-dried sample from plants elicited with 10 µM of jasmonic acid) on cytotoxicity (MTT and NR method), type of cell death (Annex V and PI test) and individual phases of the cell cycle of the healthy prostate epithelial cells (HPrEC ATCC® PCS-440-010 ™ cell line).

MTT

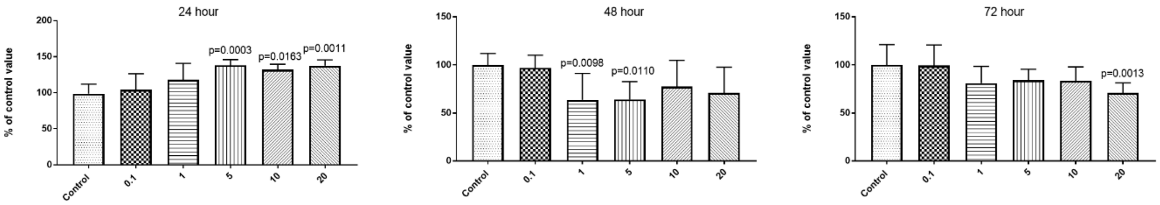

NR

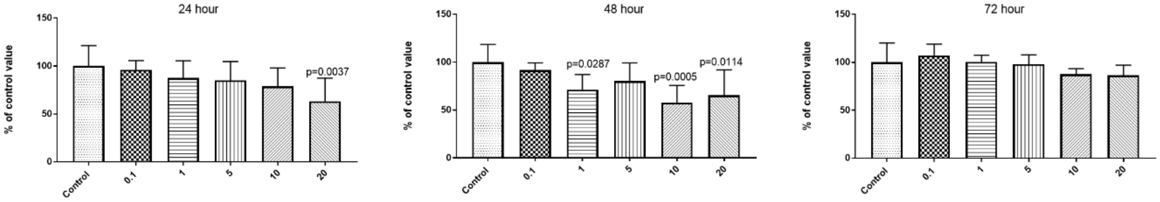

Viability

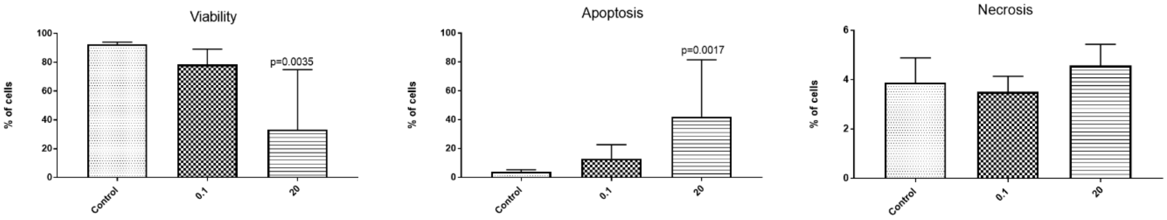

Cell Cycle

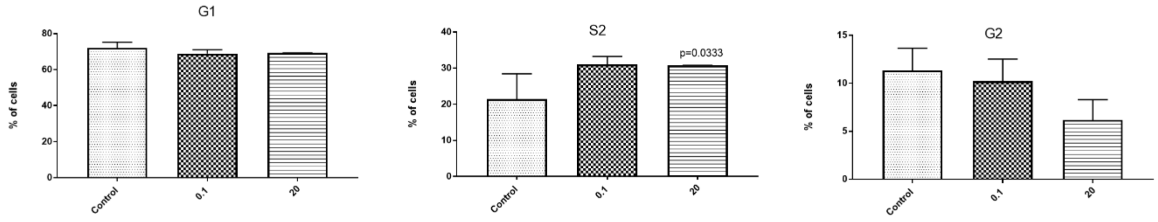

Figure S6. Impact of YEL -GD extract (sample after the *in vitro* digestion of freeze-dried sample from plants elicited with 0.1% yeast extract) on cytotoxicity (MTT and NR method), type of cell death (Annex V and PI test) and individual phases of the cell cycle of the healthy prostate epithelial cells (HPrEC ATCC® PCS-440-010 ™ cell line).

MTT

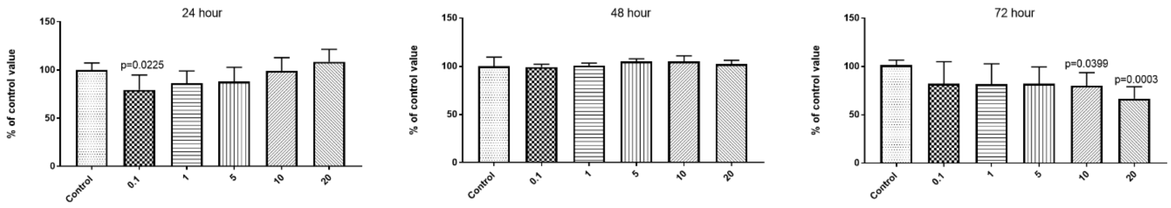

NR

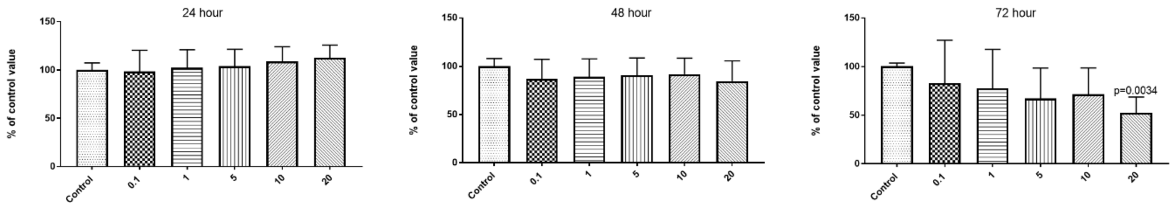

Viability

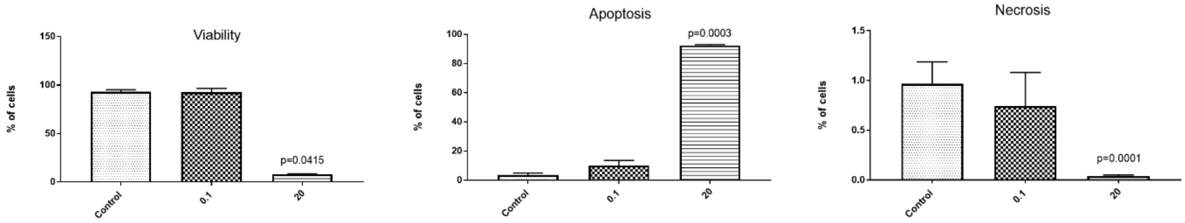

Cell Cycle

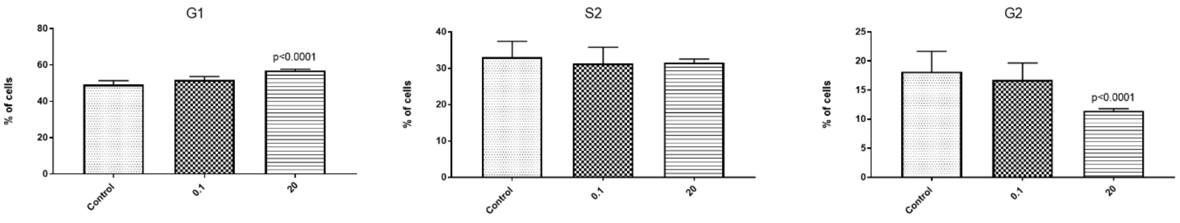

Figure S7. Impact of CL-E (ethanolic extract of control freeze-dried sample) on cytotoxicity (MTT and NR method), type of cell death (Annex V and PI test) and individual phases of the cancer gastric epithelial NCI-N87 (ATCC® CRL5822 ™) cell line.

MTT

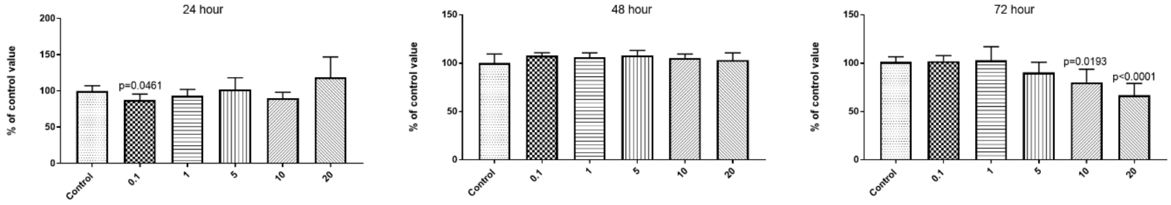

NR

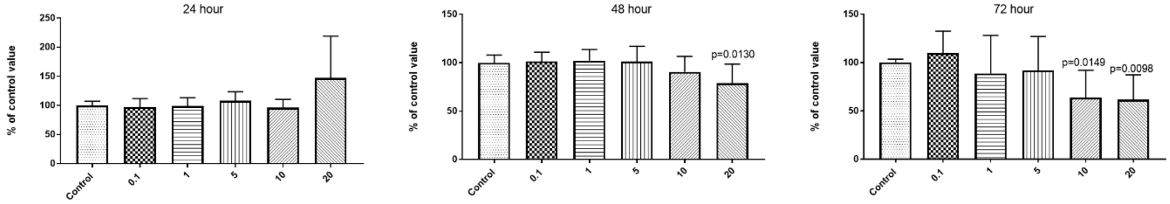

Viability

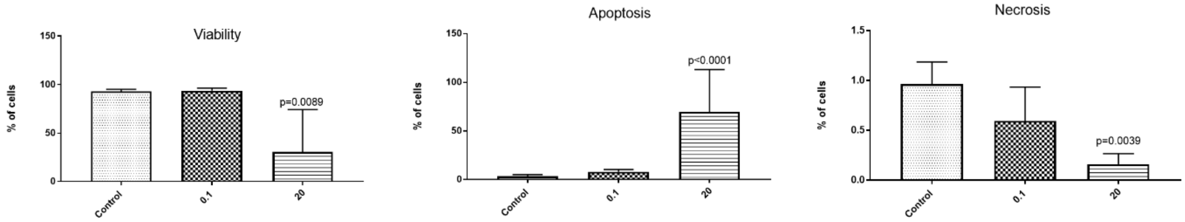

Cell Cycle

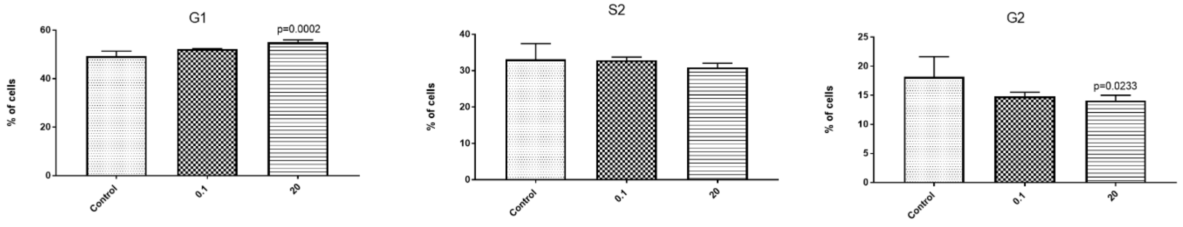

Figure S8. Impact of JAL-E (ethanolic extract of freeze-dried sample from plants elicited with 10 µM of jasmonic acid) on cytotoxicity (MTT and NR method), type of cell death (Annex V and PI test) and individual phases of the cancer gastric epithelial NCI-N87 (ATCC® CRL5822 ™) cell line.

MTT

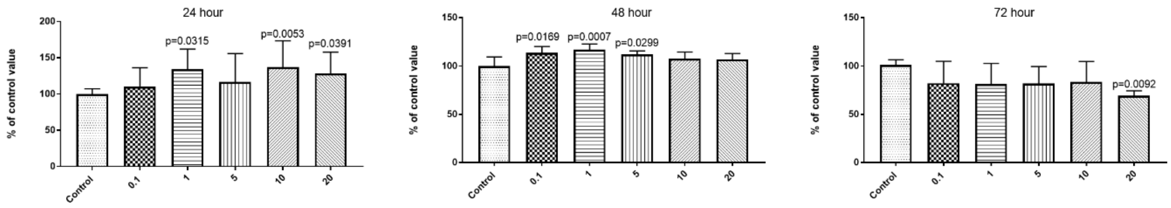

NR

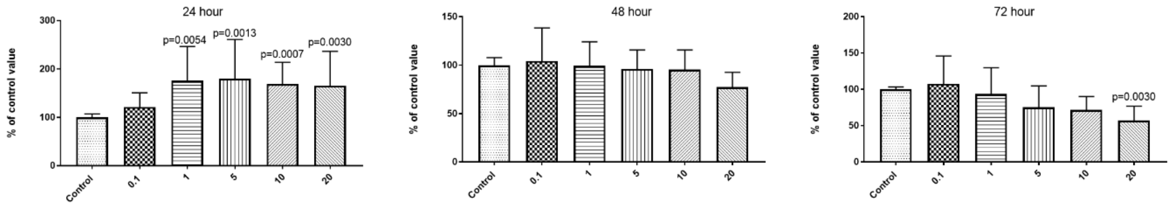

Viability

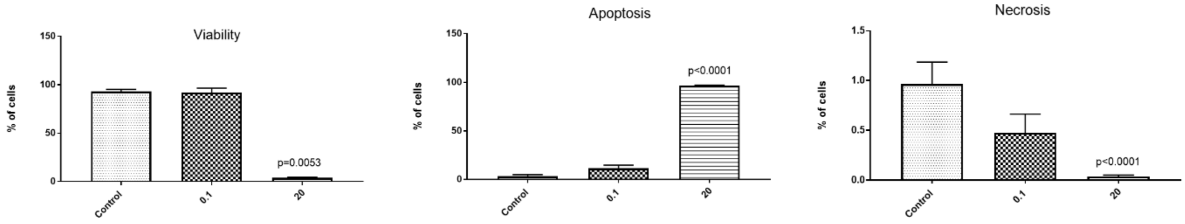

Cell Cycle

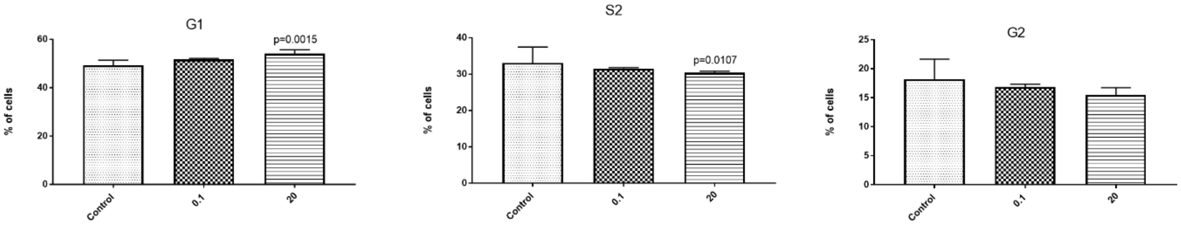

Figure S9. Impact of YEL-E (ethanolic extract of freeze-dried sample from plants elicited with 0.1% yeast extract) on cytotoxicity (MTT and NR method), type of cell death (Annex V and PI test) and individual phases of the cancer gastric epithelial NCI-N87 (ATCC® CRL5822 ™) cell line.

MTT

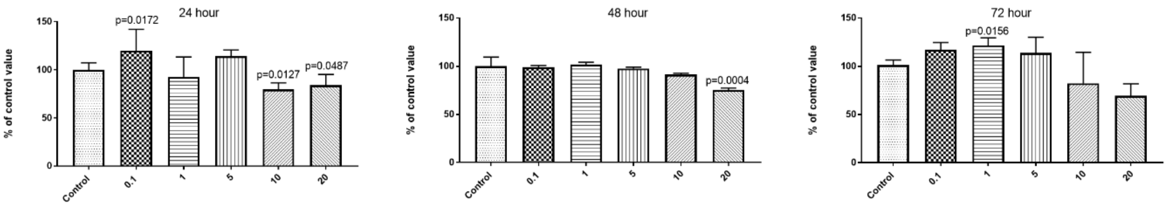

NR

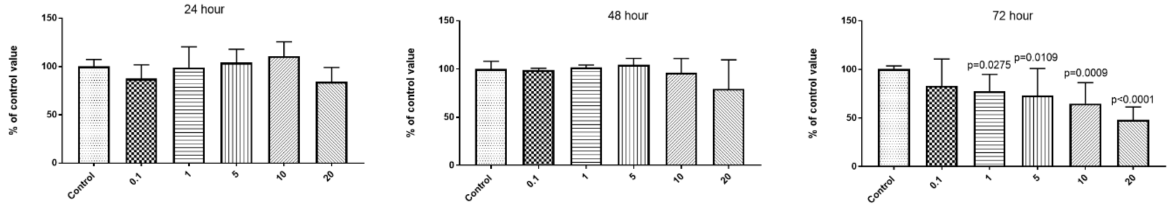

Viability

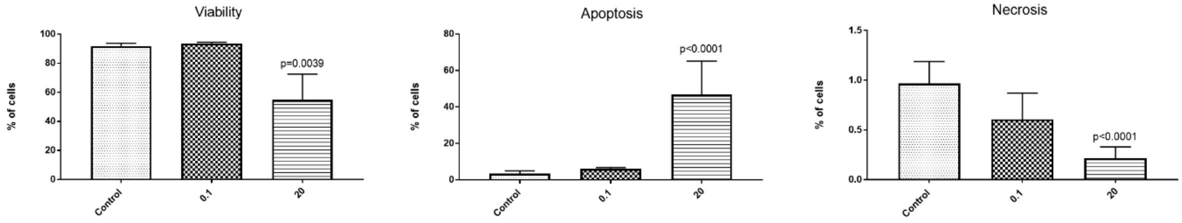

Cell Cycle

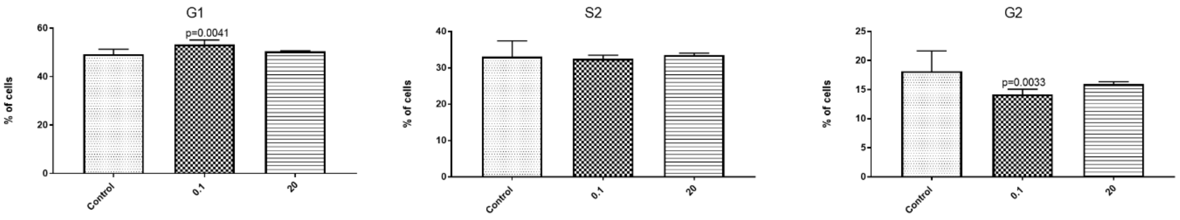

Figure S10. Impact of CL -GD extract (sample after the *in vitro* digestion of control freeze-dried sample) on cytotoxicity (MTT and NR method), type of cell death (Annex V and PI test) and individual phases of the cancer gastric epithelial NCI-N87 (ATCC® CRL5822 ™) cell line.

MTT

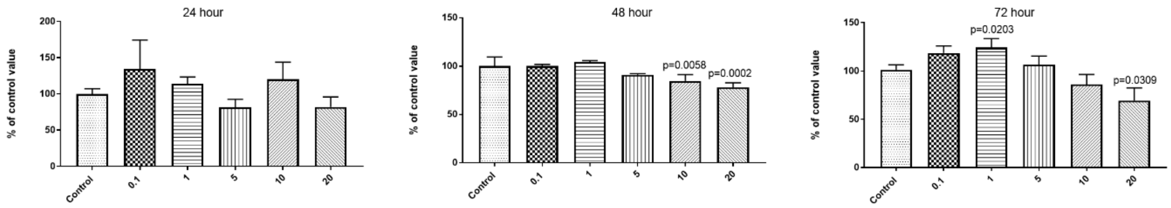

NR

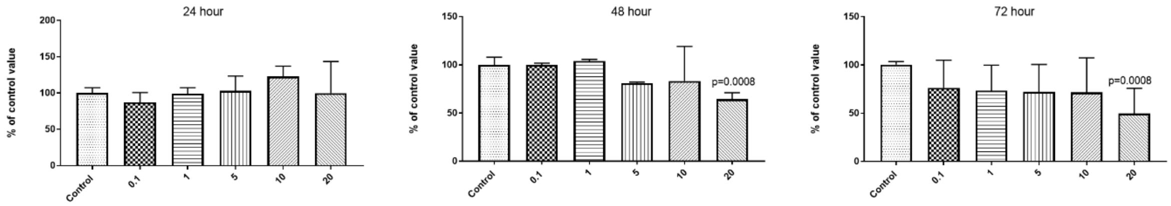

Viability

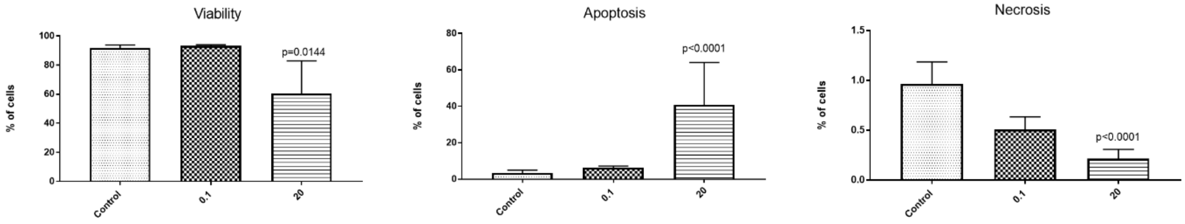

Cell Cycle

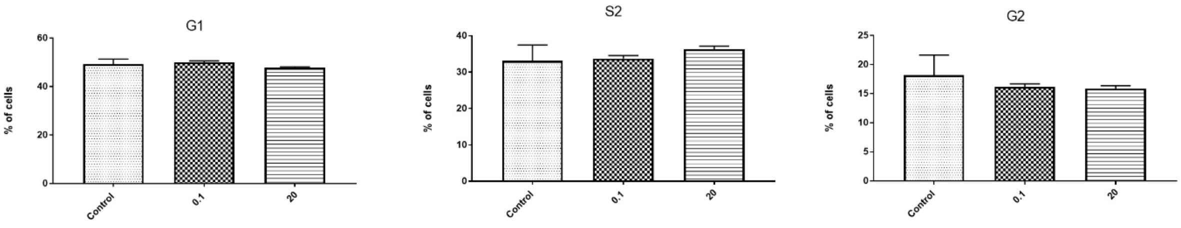

Figure S11. Impact of JAL -GD extract (sample after the *in vitro* digestion of freeze-dried sample from plants elicited with 10 µM of jasmonic acid) on cytotoxicity (MTT and NR method), type of cell death (Annex V and PI test) and individual phases of the cancer gastric epithelial NCI-N87 (ATCC® CRL5822 ™) cell line.

MTT

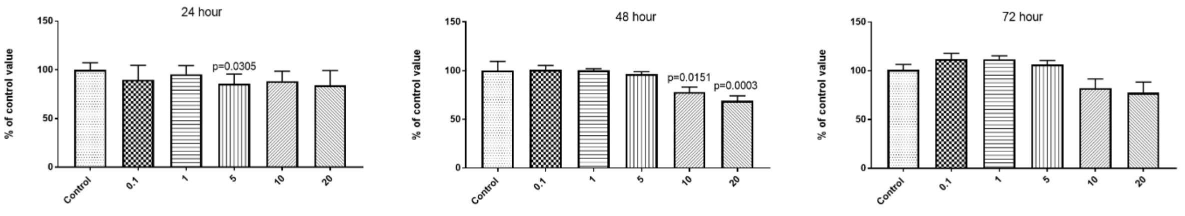

NR

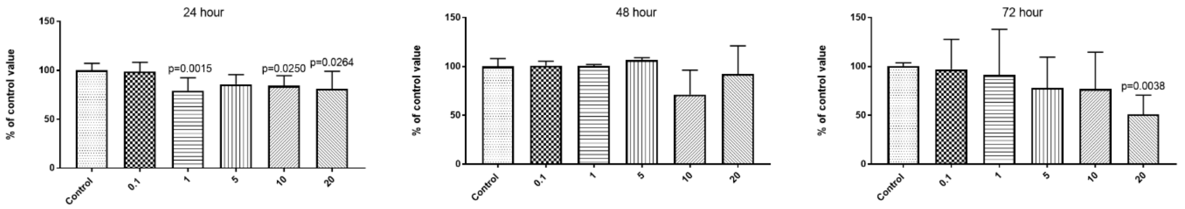

Viability

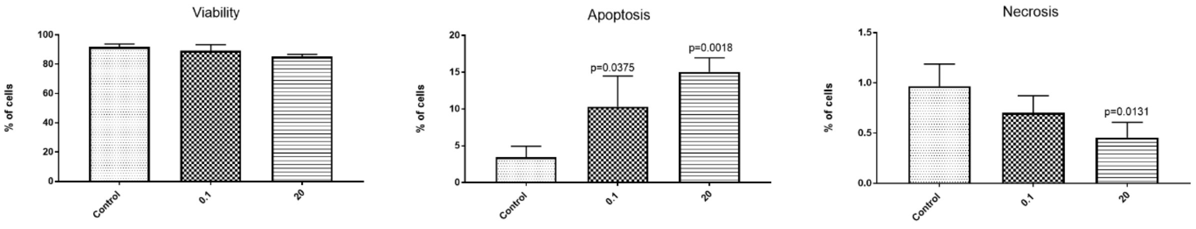

Cell Cycle

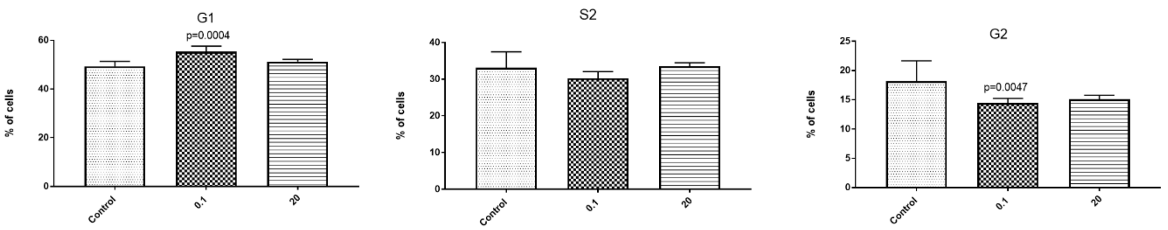

Figure S12. Impact of YEL -GD extract (sample after the *in vitro* digestion of freeze-dried sample from plants elicited with 0.1% yeast extract) on cytotoxicity (MTT and NR method), type of cell death (Annex V and PI test) and individual phases of the cancer gastric epithelial NCI-N87 (ATCC® CRL5822 ™) cell line.

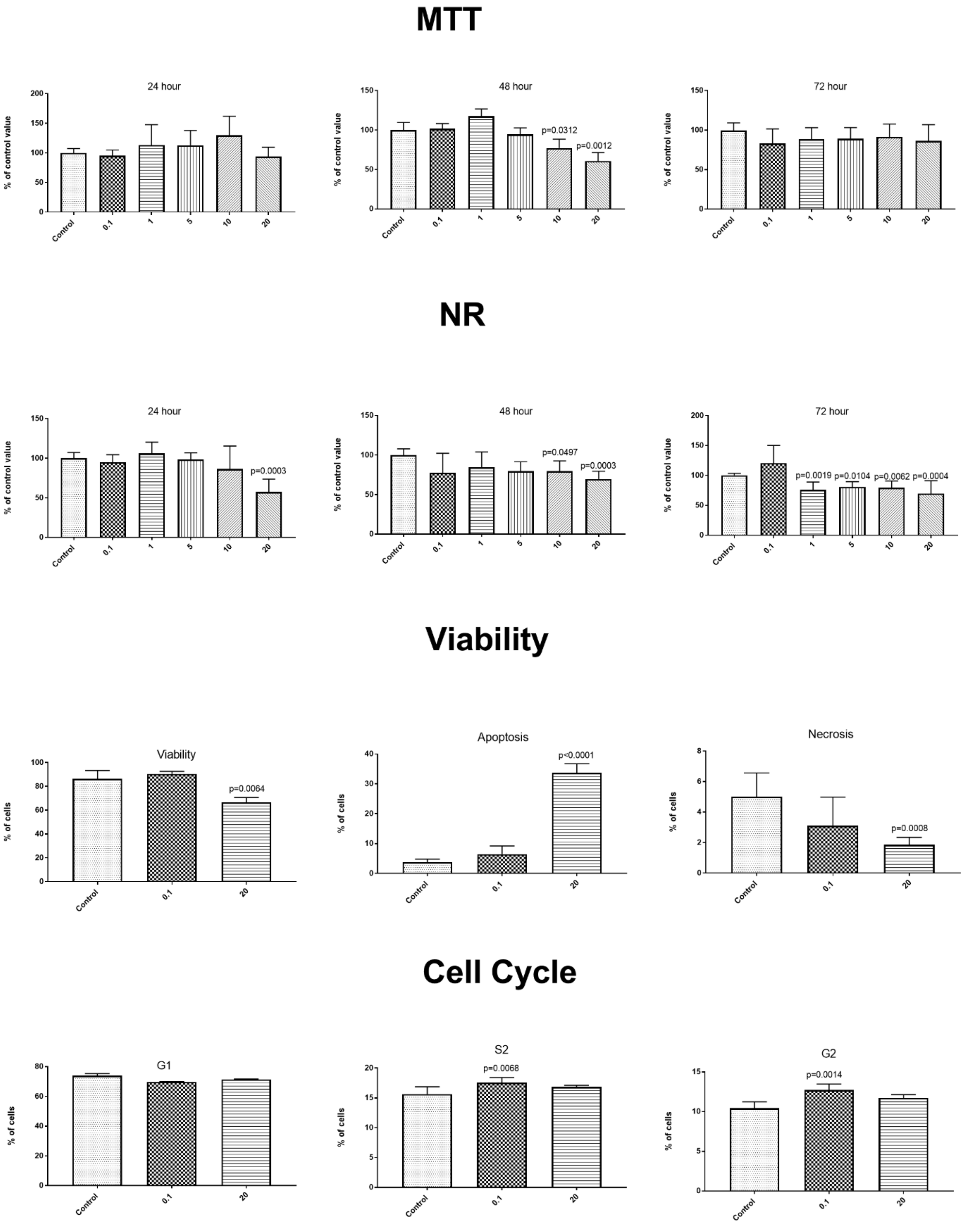

Figure S13. Impact of CL-E (ethanolic extract of control freeze-dried sample) on cytotoxicity (MTT and NR method), type of cell death (Annex V and PI test) and individual phases of the prostate cancer line VCaP – (ATCC® CRL-2876 ™).

MTT

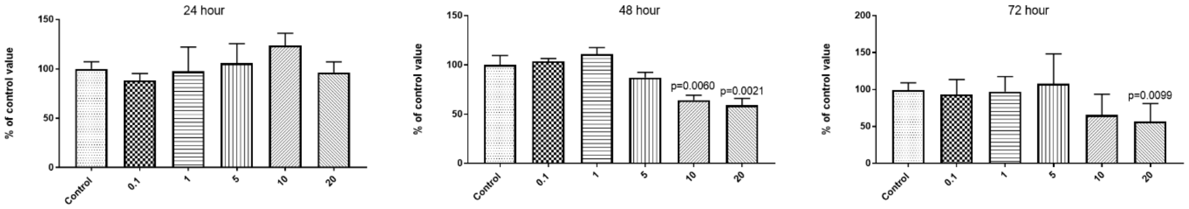

NR

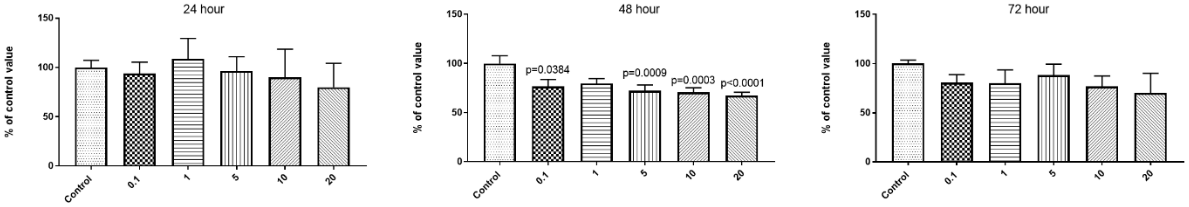

Viability

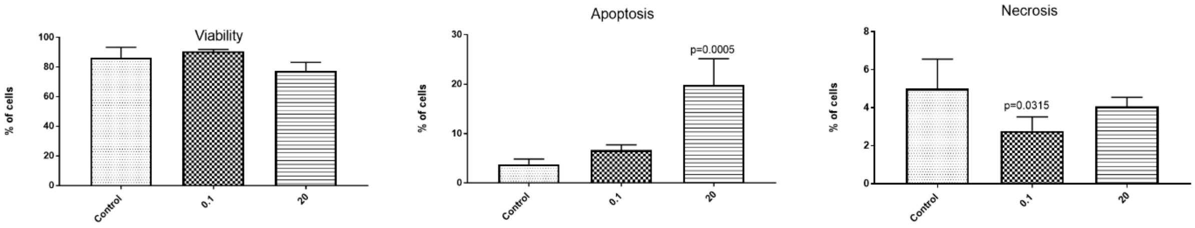

Cell Cycle

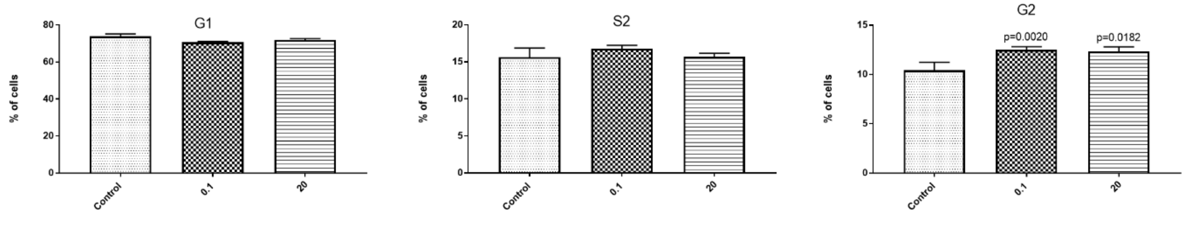

Figure S14. Impact of JAL-E (ethanolic extract of freeze-dried sample from plants elicited with 10 µM of jasmonic acid) on cytotoxicity (MTT and NR method), type of cell death (Annex V and PI test) and individual phases of the prostate cancer line VCaP – (ATCC® CRL-2876 ™).

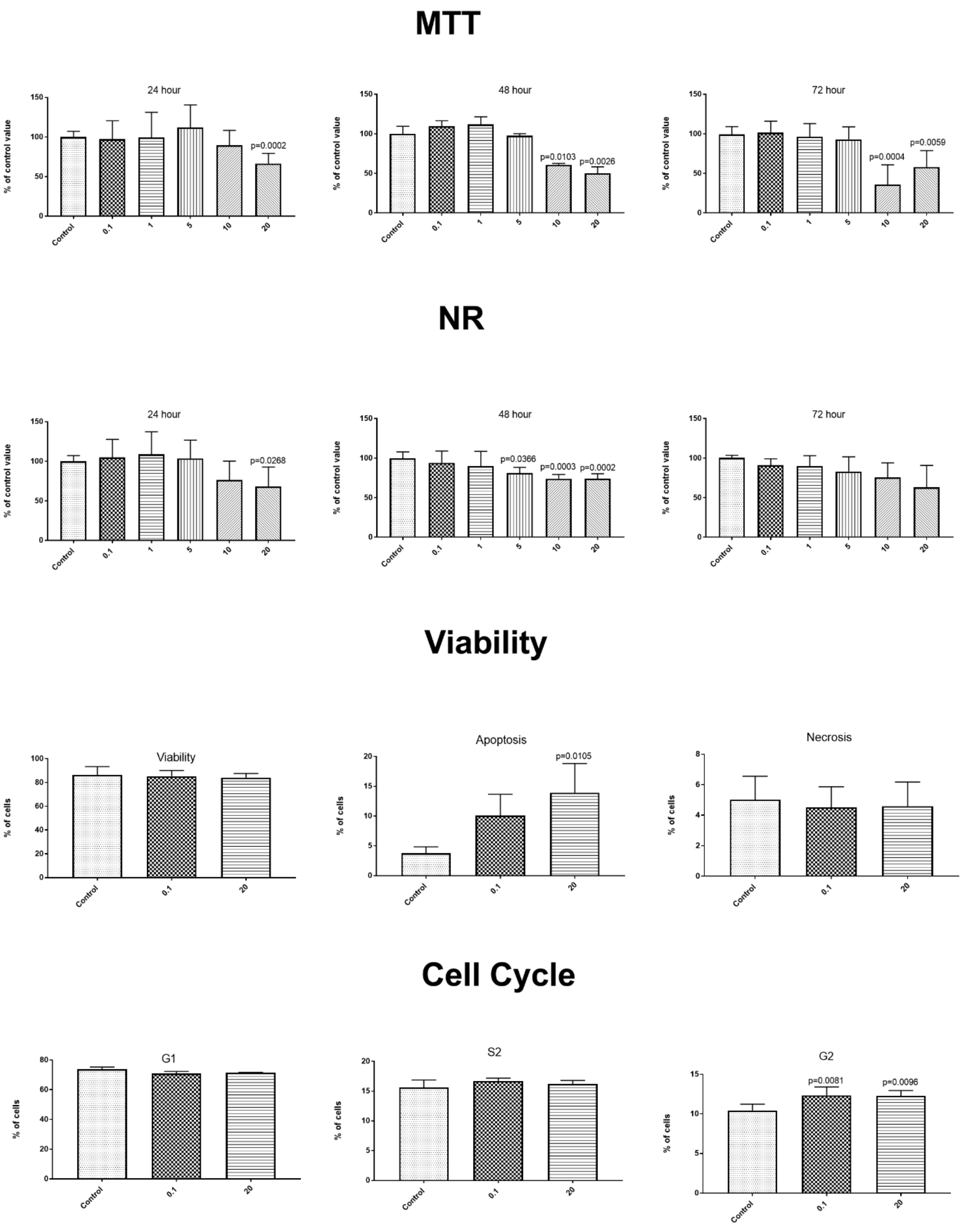

Figure S15. Impact of YEL-E (ethanolic extract of freeze-dried sample from plants elicited with 0.1% yeast extract) on cytotoxicity (MTT and NR method), type of cell death (Annex V and PI test) and individual phases of the prostate cancer line VCaP – (ATCC® CRL-2876 ™).

MTT

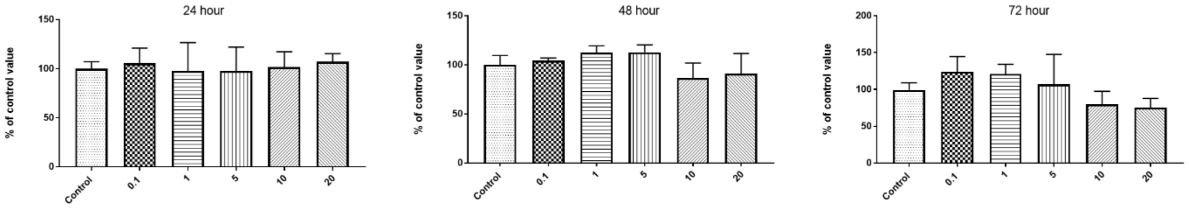

NR

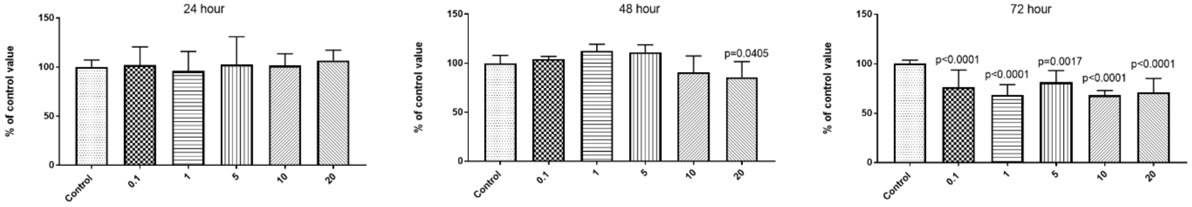

Viability

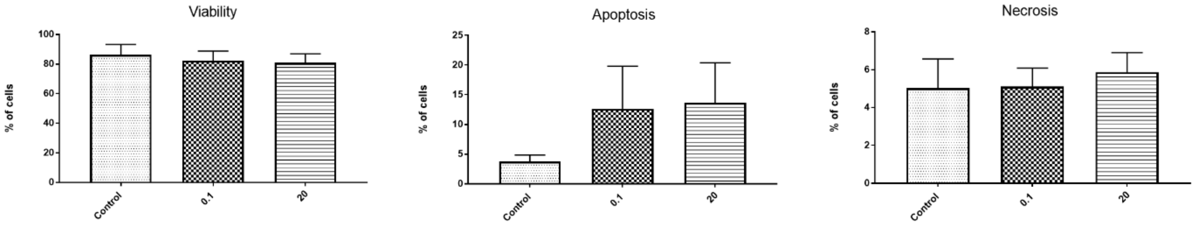

Cell Cycle

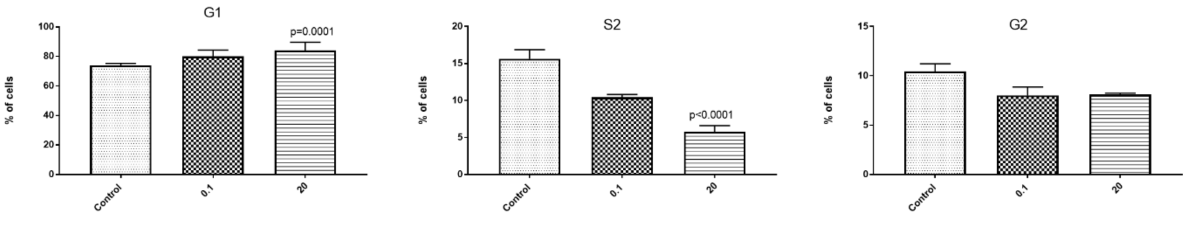

Figure S16. Impact of CL-GD extract (sample after the *in vitro* digestion of control freeze-dried sample) on cytotoxicity (MTT and NR method), type of cell death (Annex V and PI test) and individual phases of the prostate cancer line VCaP – (ATCC® CRL-2876 ™).

MTT

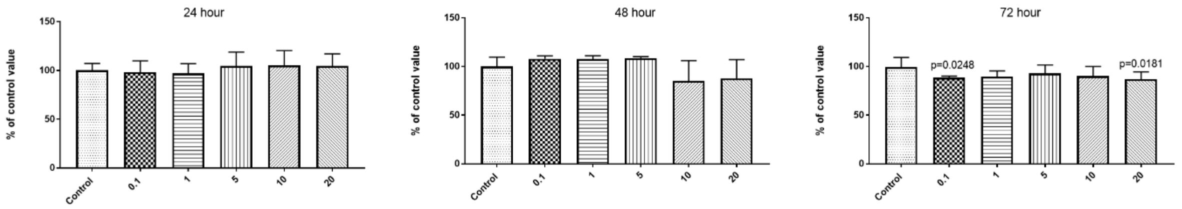

NR

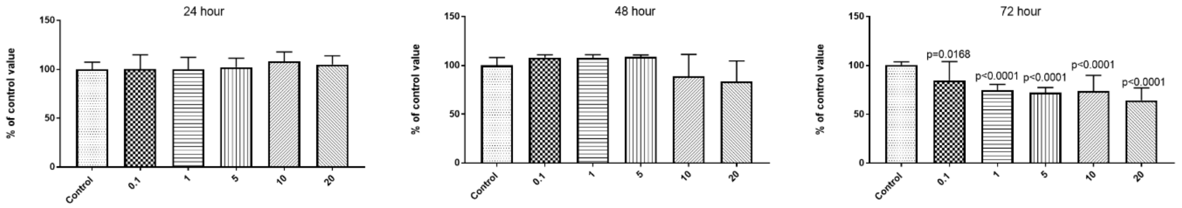

Viability

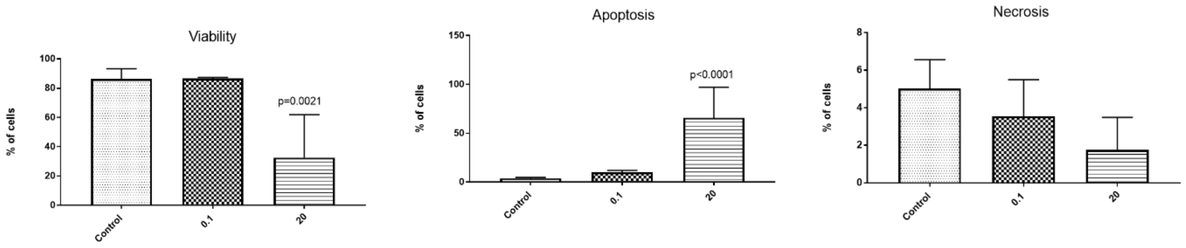

Cell Cycle

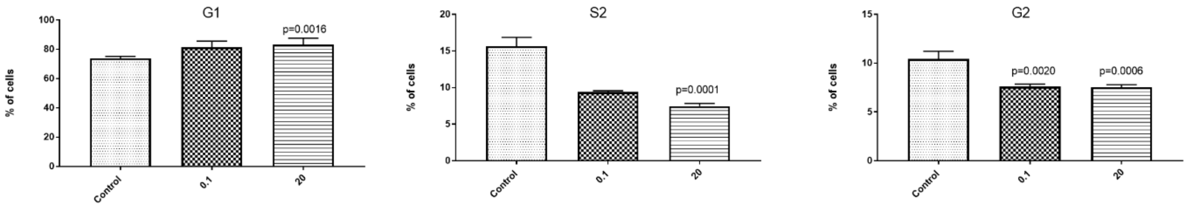

Figure S17. Impact of JAL -GD extract (sample after the *in vitro* digestion of freeze-dried sample from plants elicited with 10 µM of jasmonic acid) on cytotoxicity (MTT and NR method), type of cell death (Annex V and PI test) and individual phases of the prostate cancer line VCaP – (ATCC® CRL-2876 ™).

MTT

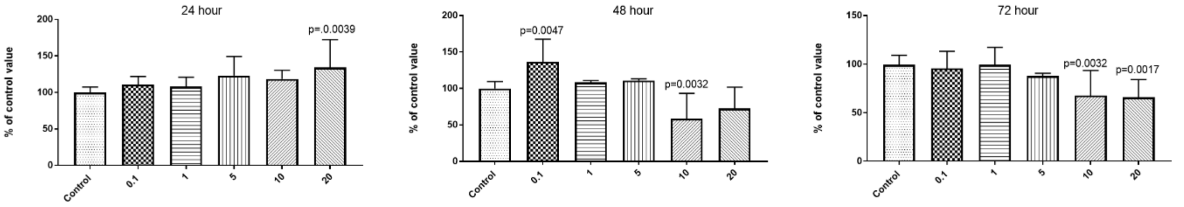

NR

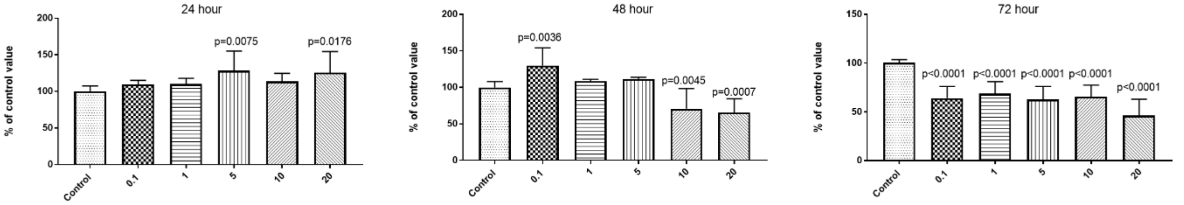

Viability

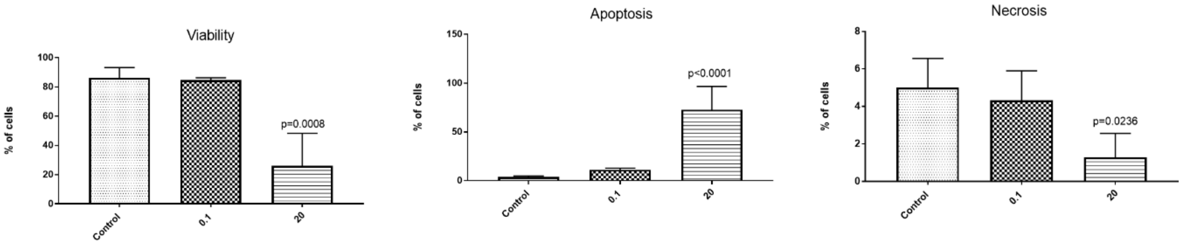

Cell Cycle

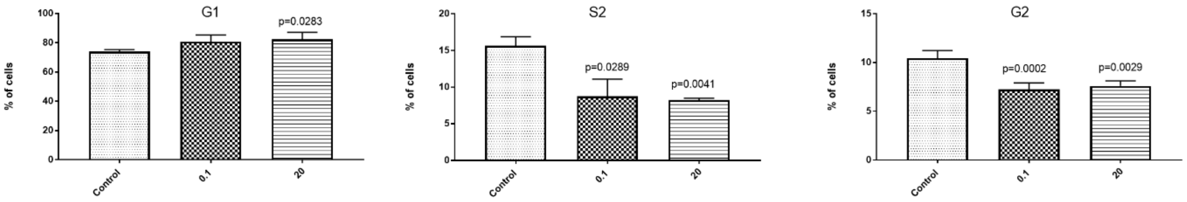

Figure S18. Impact of YEL -GD extract (sample after the *in vitro* digestion of freeze-dried sample from plants elicited with 0.1% yeast extract) on cytotoxicity (MTT and NR method), type of cell death (Annex V and PI test) and individual phases of the prostate cancer line VCaP – (ATCC® CRL-2876 ™).

MTT

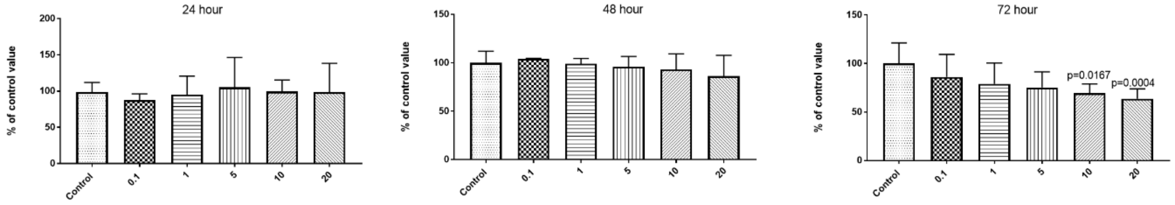

NR

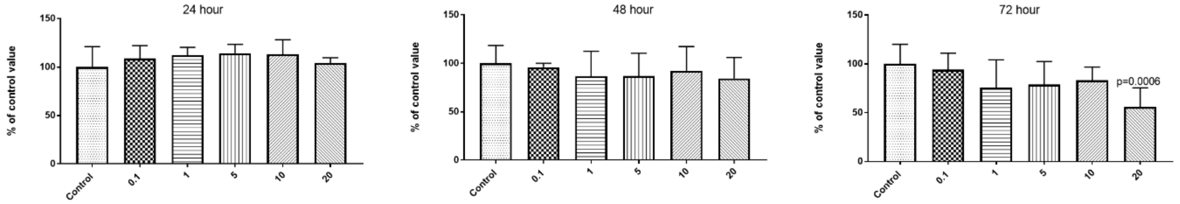

Viability

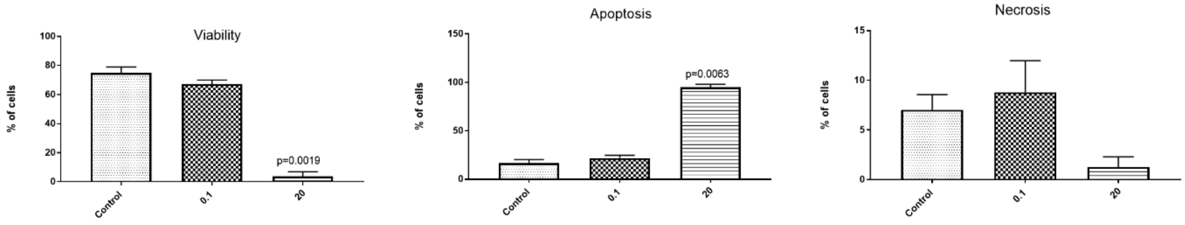

Cell Cycle

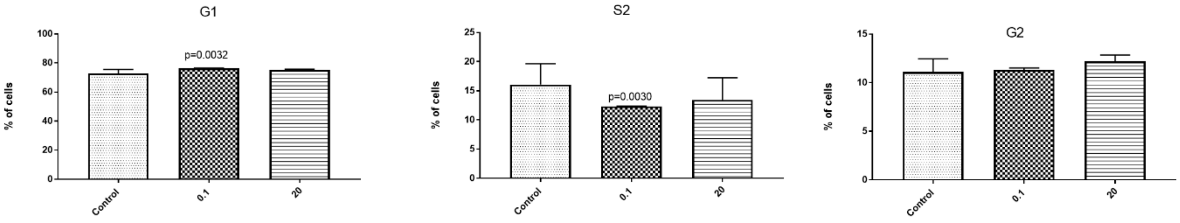

Figure S19. Impact of CM-E (ethanolic extract of control microwave-dried sample) on cytotoxicity (MTT and NR method), type of cell death (Annex V and PI test) and individual phases of the cell cycle of the healthy prostate epithelial cells (HPrEC ATCC® PCS-440-010 ™ cell line).

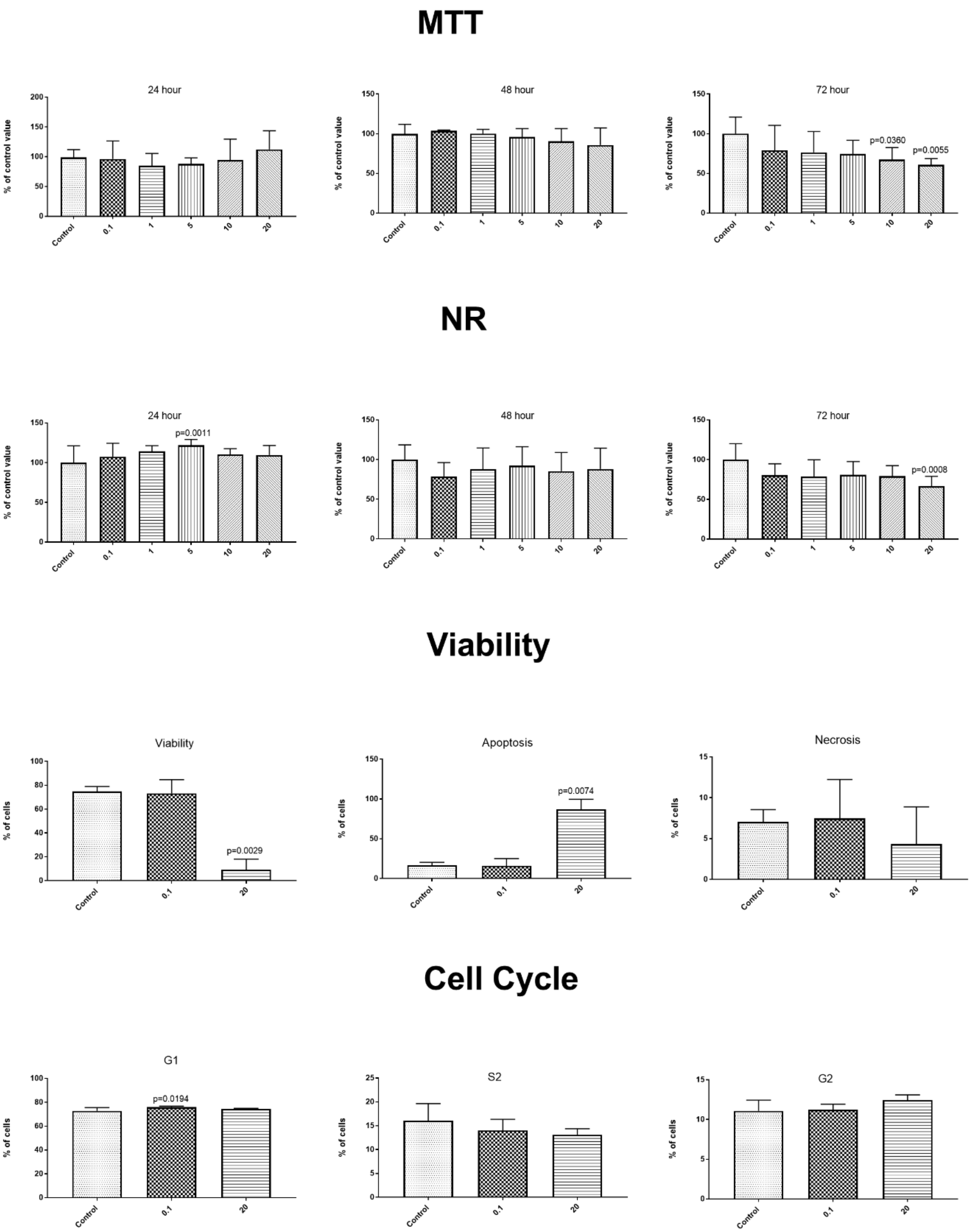

Figure S20. Impact of JAM-E (ethanolic extract of microwave-dried sample from plants elicited with 10 µM of jasmonic acid) on cytotoxicity (MTT and NR method), type of cell death (Annex V and PI test) and individual phases of the cell cycle of the healthy prostate epithelial cells (HPrEC ATCC® PCS-440-010 ™ cell line).

MTT

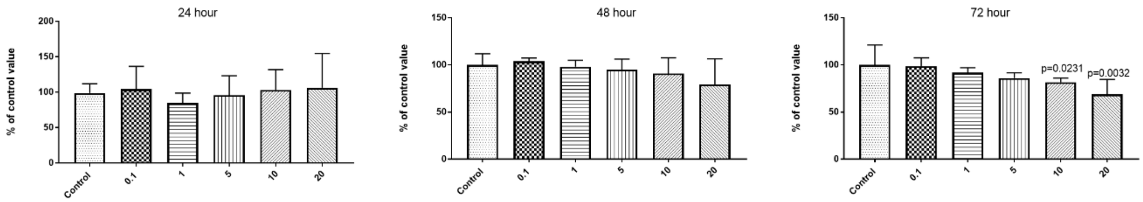

NR

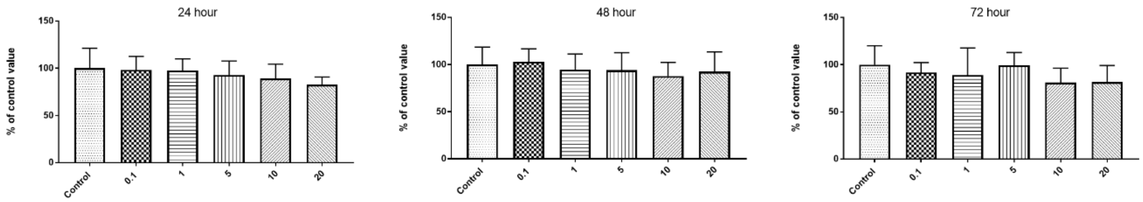

Viability

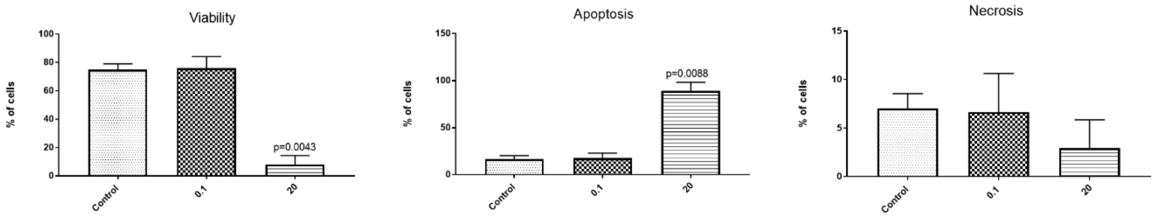

Cell Cycle

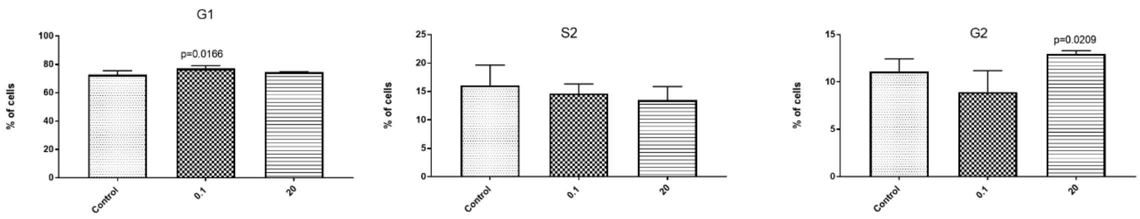

Figure S21. Impact of YEM-E (ethanolic extract of microwave-dried sample from plants elicited with 0.1% yeast extract) on cytotoxicity (MTT and NR method), type of cell death (Annex V and PI test) and individual phases of the cell cycle of the healthy prostate epithelial cells (HPrEC ATCC® PCS-440-010 ™ cell line).

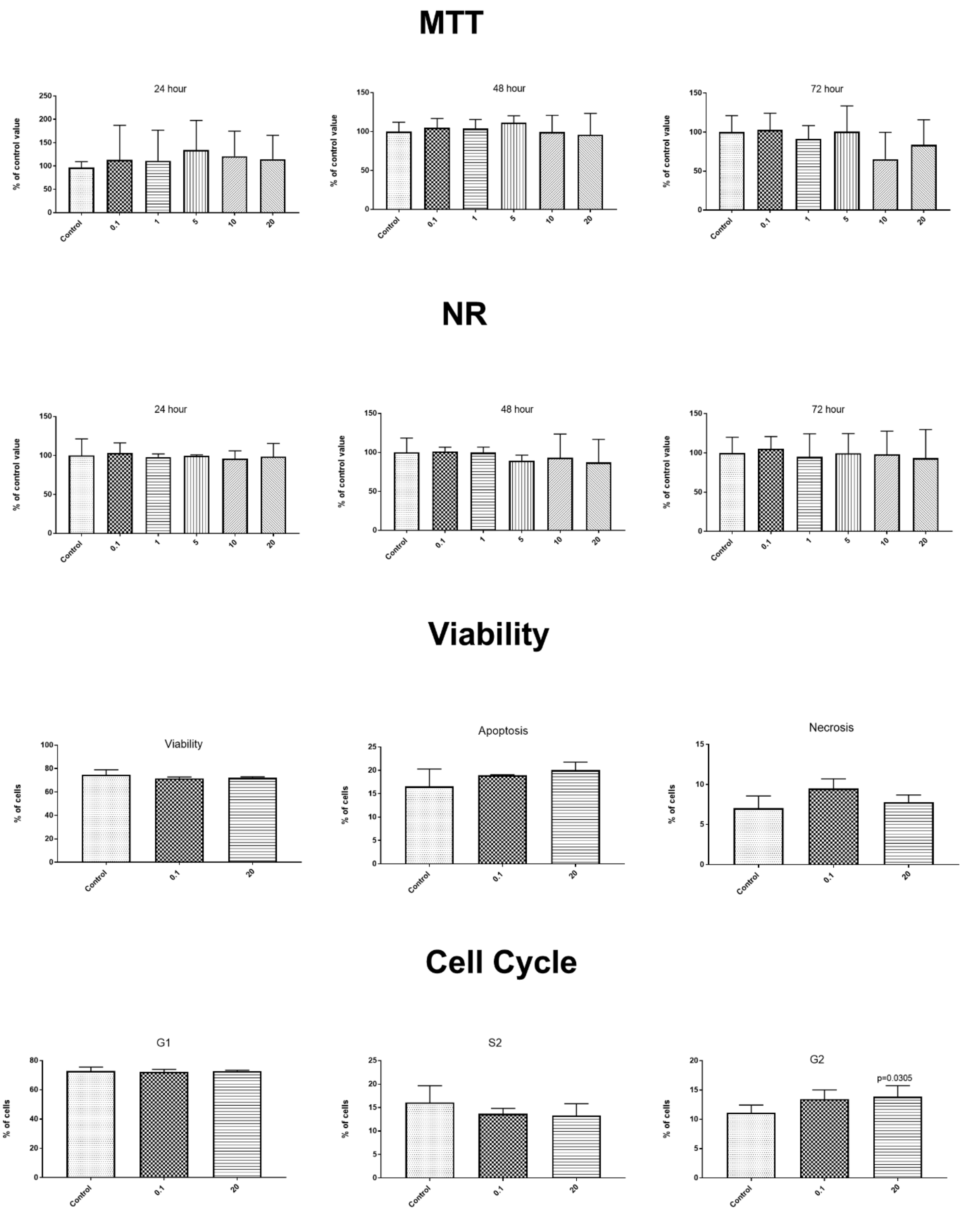

Figure S22. Impact of CM-GD extract (sample after the *in vitro* digestion of control microwave-dried sample) on cytotoxicity (MTT and NR method), type of cell death (Annex V and PI test) and individual phases of the cell cycle of the healthy prostate epithelial cells (HPrEC ATCC® PCS-440-010 ™ cell line).

MTT

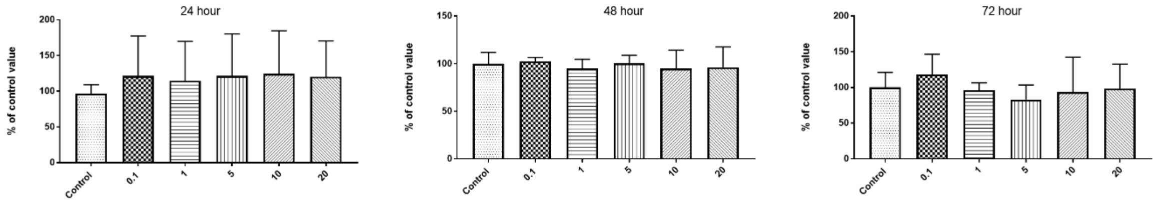

NR

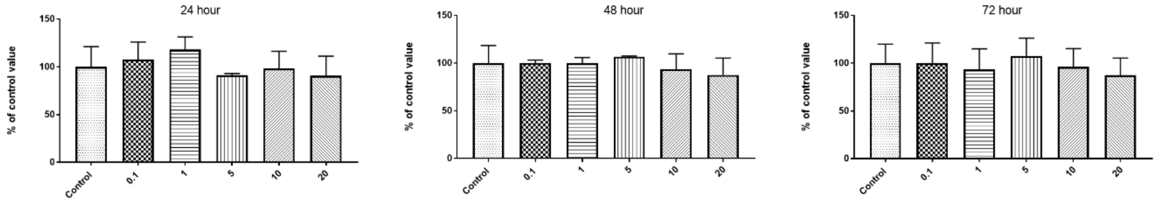

Viability

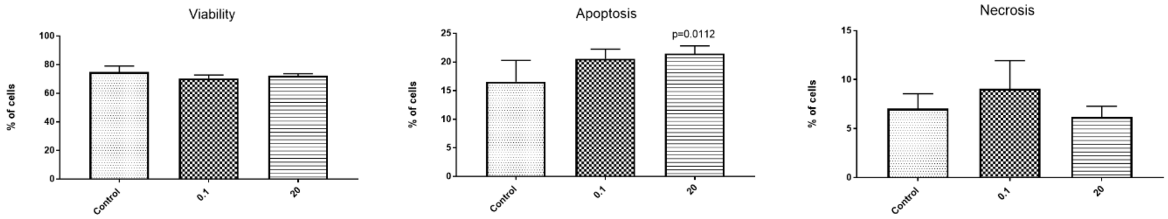

Cell Cycle

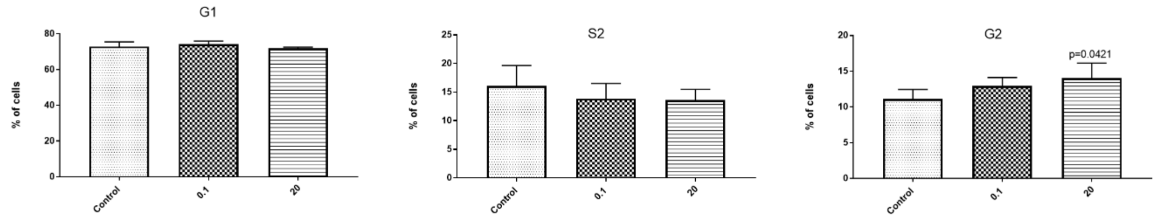

Figure S23. Impact of JAM-GD (sample after the *in vitro* digestion of microwave-dried sample from plants elicited with 10 µM of jasmonic acid) on cytotoxicity (MTT and NR method), type of cell death (Annex V and PI test) and individual phases of the cell cycle of the healthy prostate epithelial cells (HPrEC ATCC® PCS-440-010 ™ cell line).

MTT

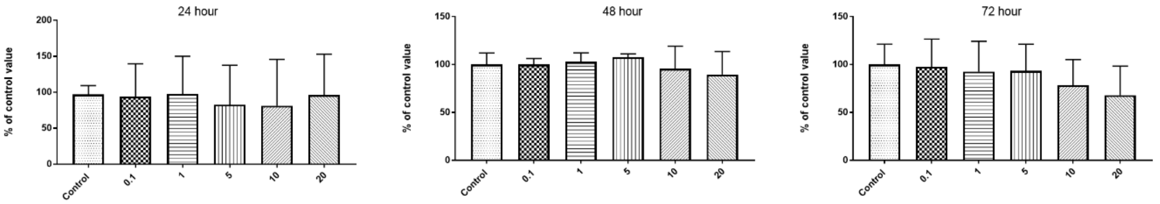

NR

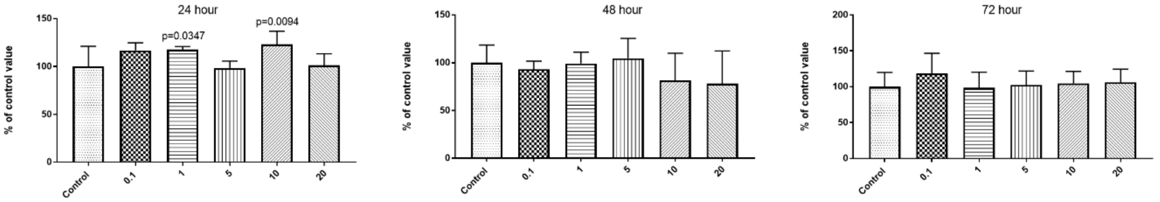

Viability

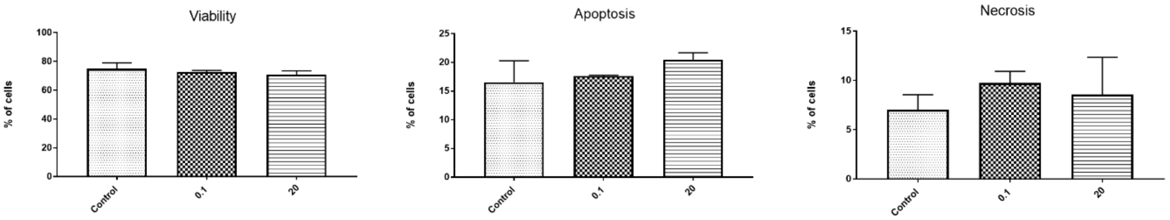

Cell Cycle

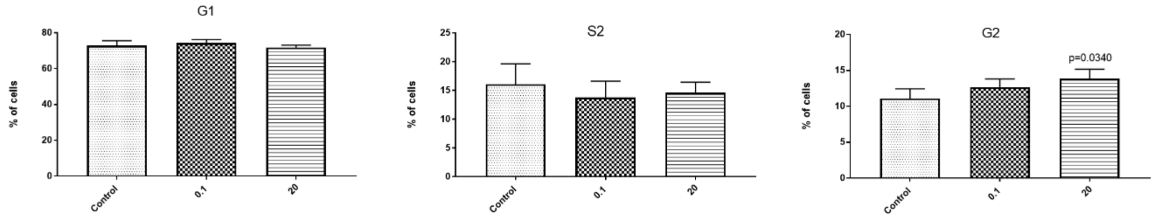

Figure S24. Impact of YEM -GD extract (sample after the *in vitro* digestion of microwave-dried sample from plants elicited with 0.1% yeast extract) on cytotoxicity (MTT and NR method), type of cell death (Annex V and PI test) and individual phases of the cell cycle of the healthy prostate epithelial cells (HPrEC ATCC® PCS-440-010 ™ cell line).

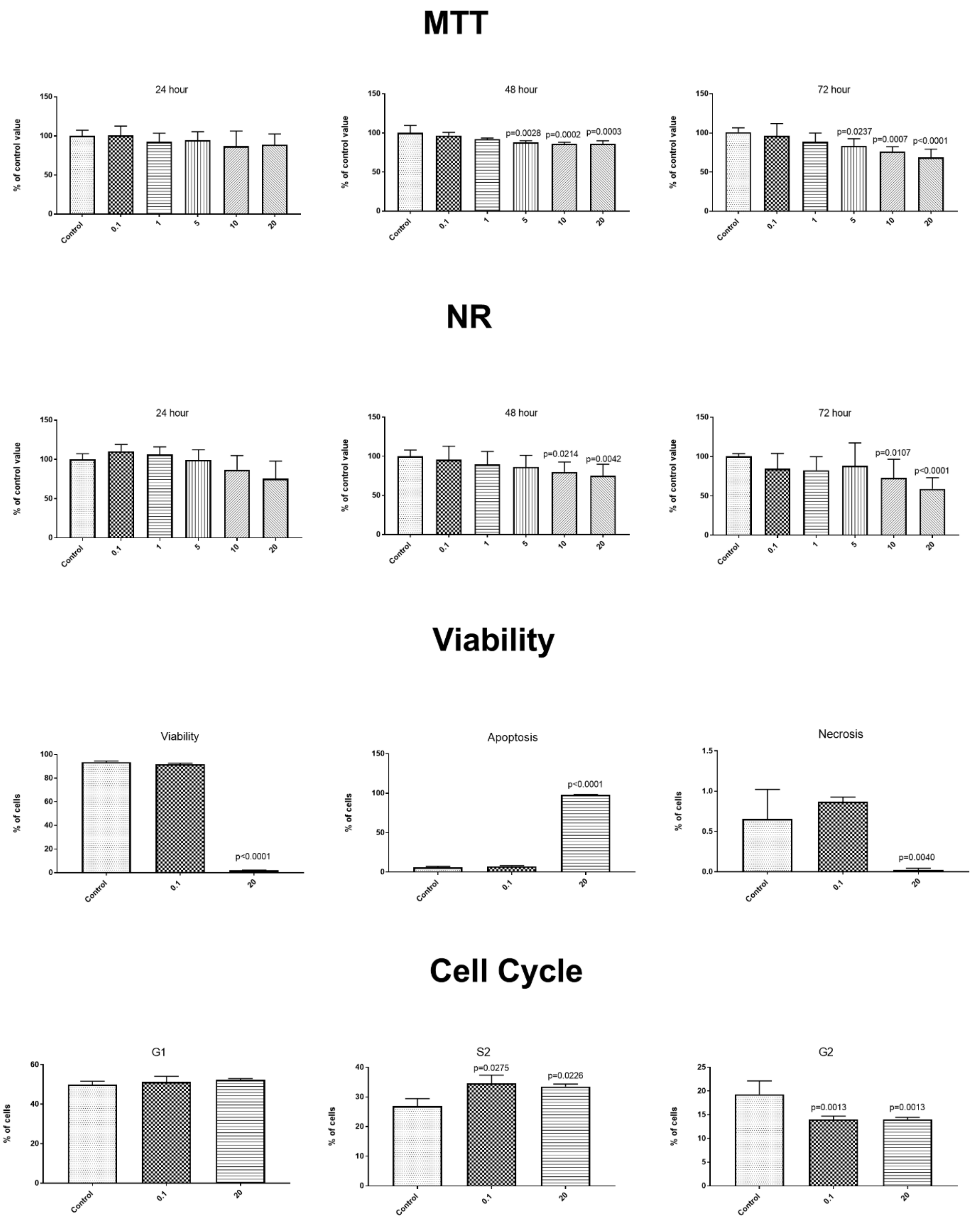

Figure S25. Impact of CM-E (ethanolic extract of control microwave-dried sample) on cytotoxicity (MTT and NR method), type of cell death (Annex V and PI test) and individual phases of the cancer gastric epithelial NCI-N87 (ATCC® CRL5822 ™) cell line.

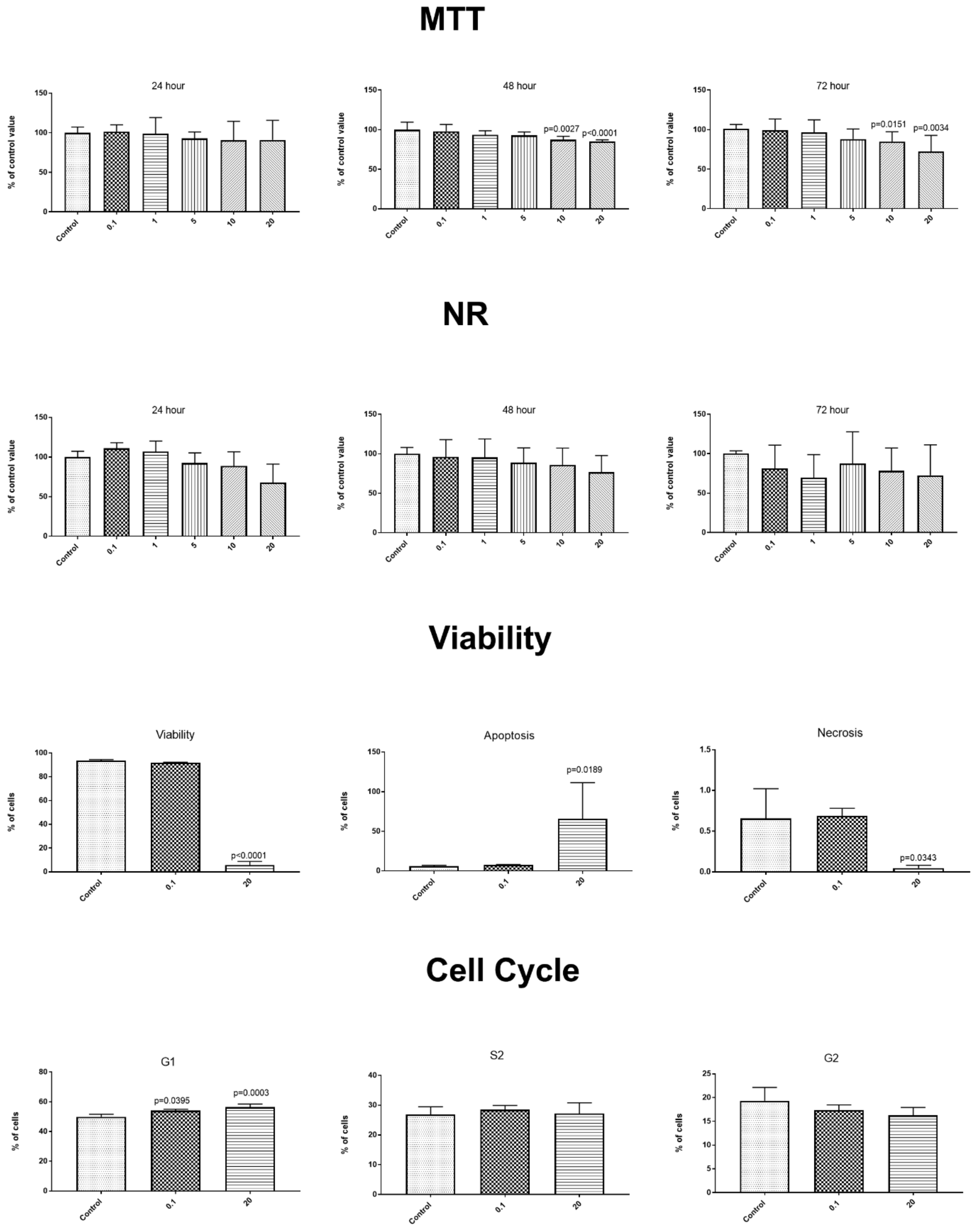

Figure S26. Impact of JAM-E (ethanolic extract of microwave-dried sample from plants elicited with 10 µM of jasmonic acid) on cytotoxicity (MTT and NR method), type of cell death (Annex V and PI test) and individual phases of the cancer gastric epithelial NCI-N87 (ATCC® CRL5822 ™) cell line.

MTT

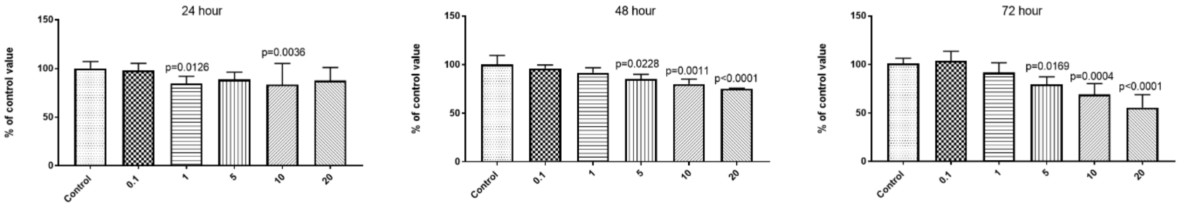

NR

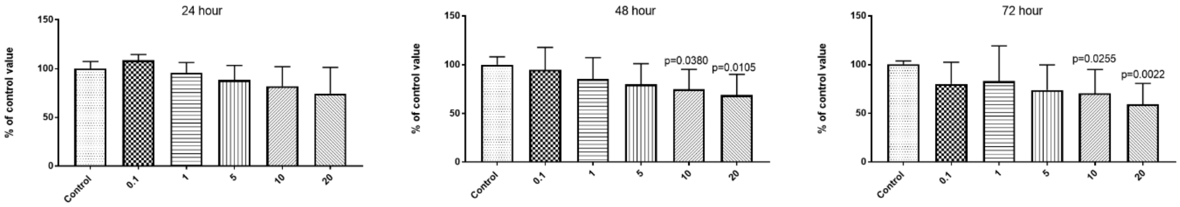

Viability

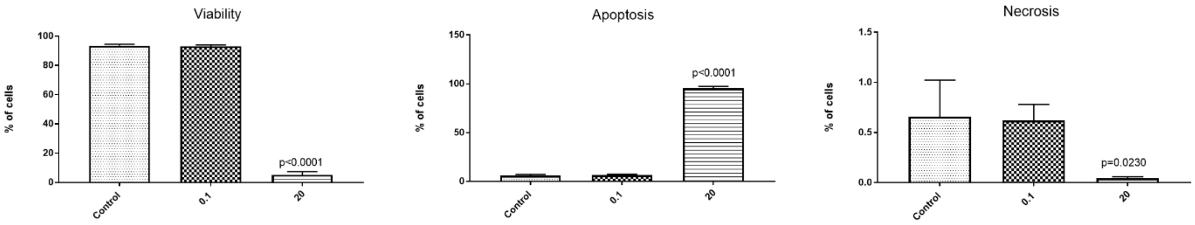

Cell Cycle

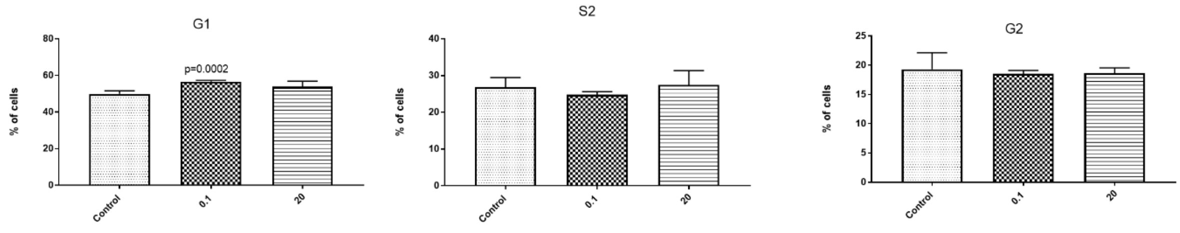

Figure S27. Impact of YEM-E (ethanolic extract of microwave-dried sample from plants elicited with 0.1% yeast extract) on cytotoxicity (MTT and NR method), type of cell death (Annex V and PI test) and individual phases of the cancer gastric epithelial NCI-N87 (ATCC® CRL5822 ™) cell line.

MTT

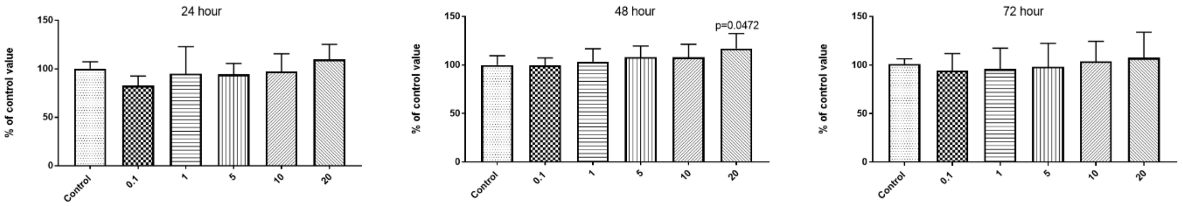

NR

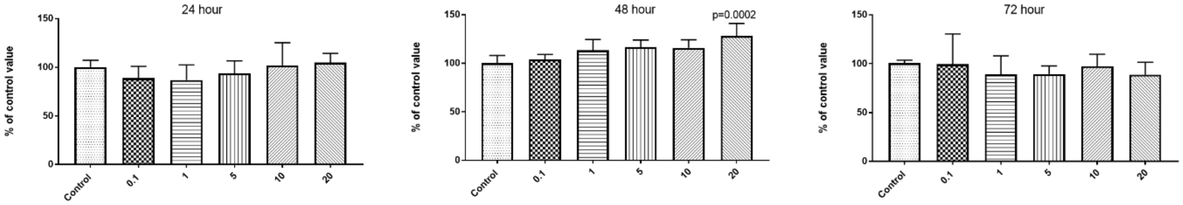

Viability

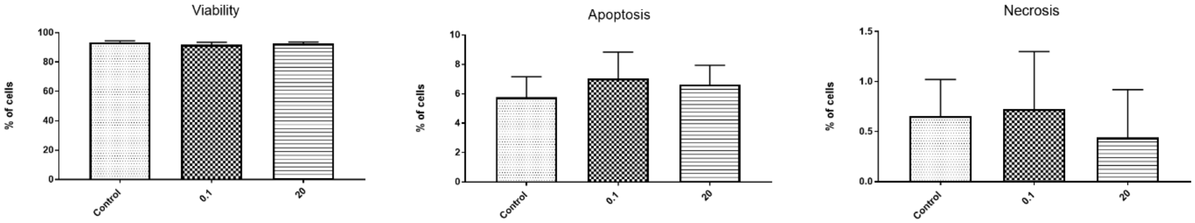

Cell Cycle

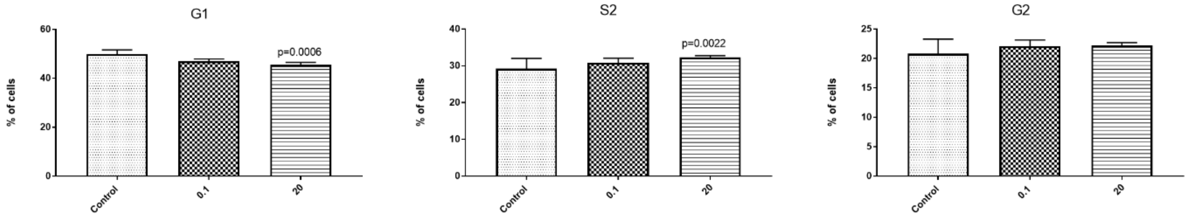

Figure S28. Impact of CM -GD extract (sample after the *in vitro* digestion of control microwave-dried sample) on cytotoxicity (MTT and NR method), type of cell death (Annex V and PI test) and individual phases of the cancer gastric epithelial NCI-N87 (ATCC® CRL5822 ™) cell line.

MTT

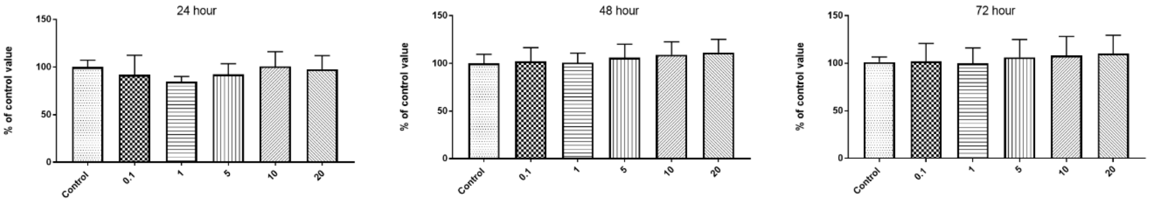

NR

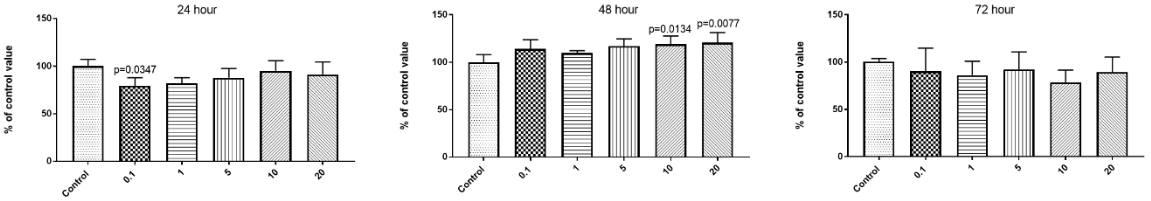

Viability

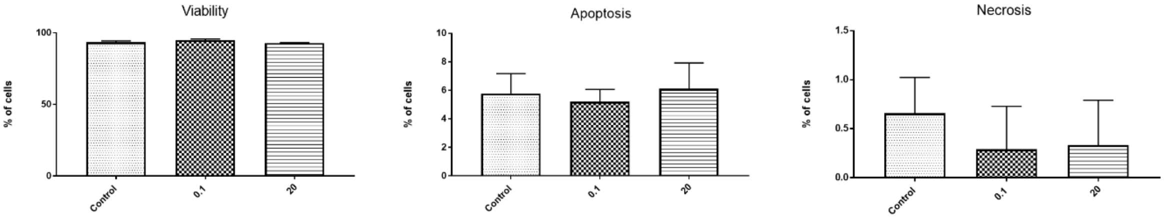

Cell Cycle

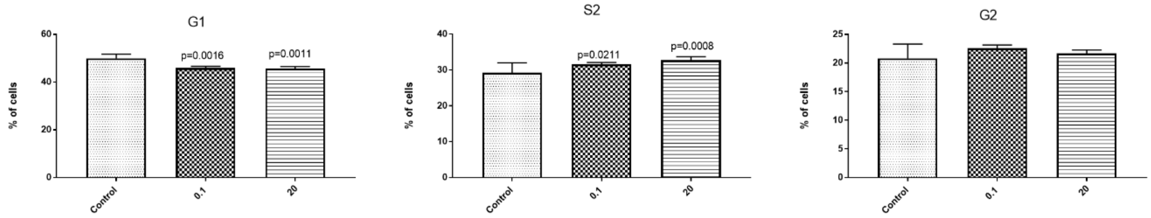

Figure S29. Impact of JAM -GD extract (sample after the *in vitro* digestion of microwave-dried sample from plants elicited with 10 µM of jasmonic acid) on cytotoxicity (MTT and NR method), type of cell death (Annex V and PI test) and individual phases of the cancer gastric epithelial NCI-N87 (ATCC® CRL5822 ™) cell line.

MTT

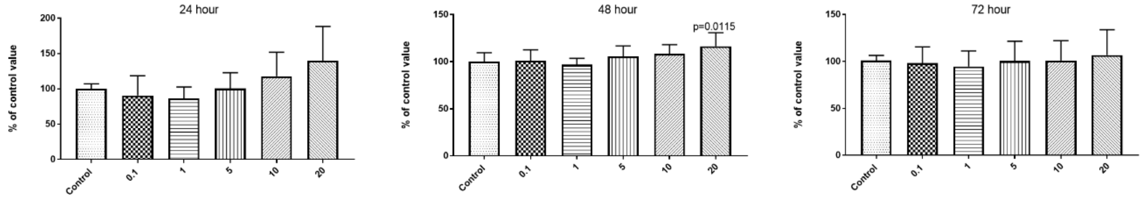

NR

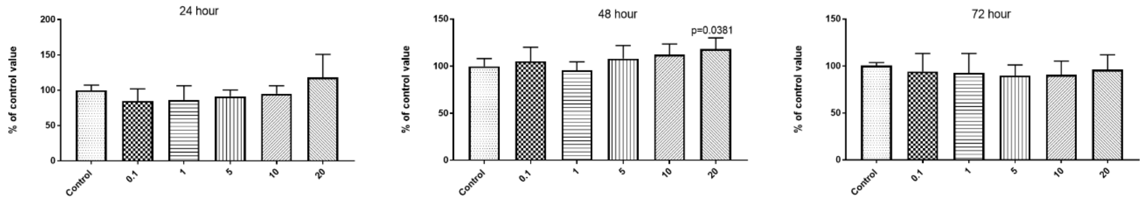

Viability

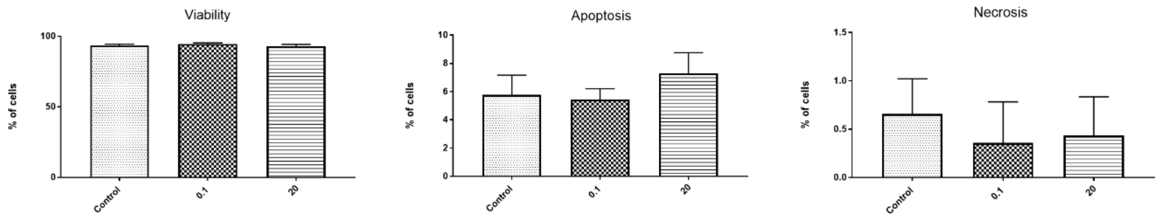

Cell Cycle

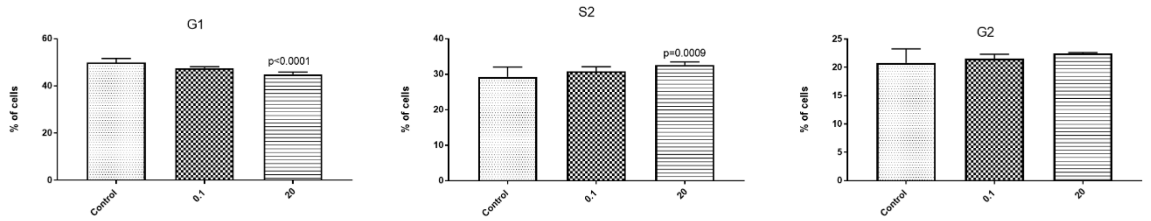

Figure S30. Impact of YEM -GD extract (sample after the *in vitro* digestion of microwave-dried sample from plants elicited with 0.1% yeast extract) on cytotoxicity (MTT and NR method), type of cell death (Annex V and PI test) and individual phases of the cancer gastric epithelial NCI-N87 (ATCC® CRL5822 ™) cell line.

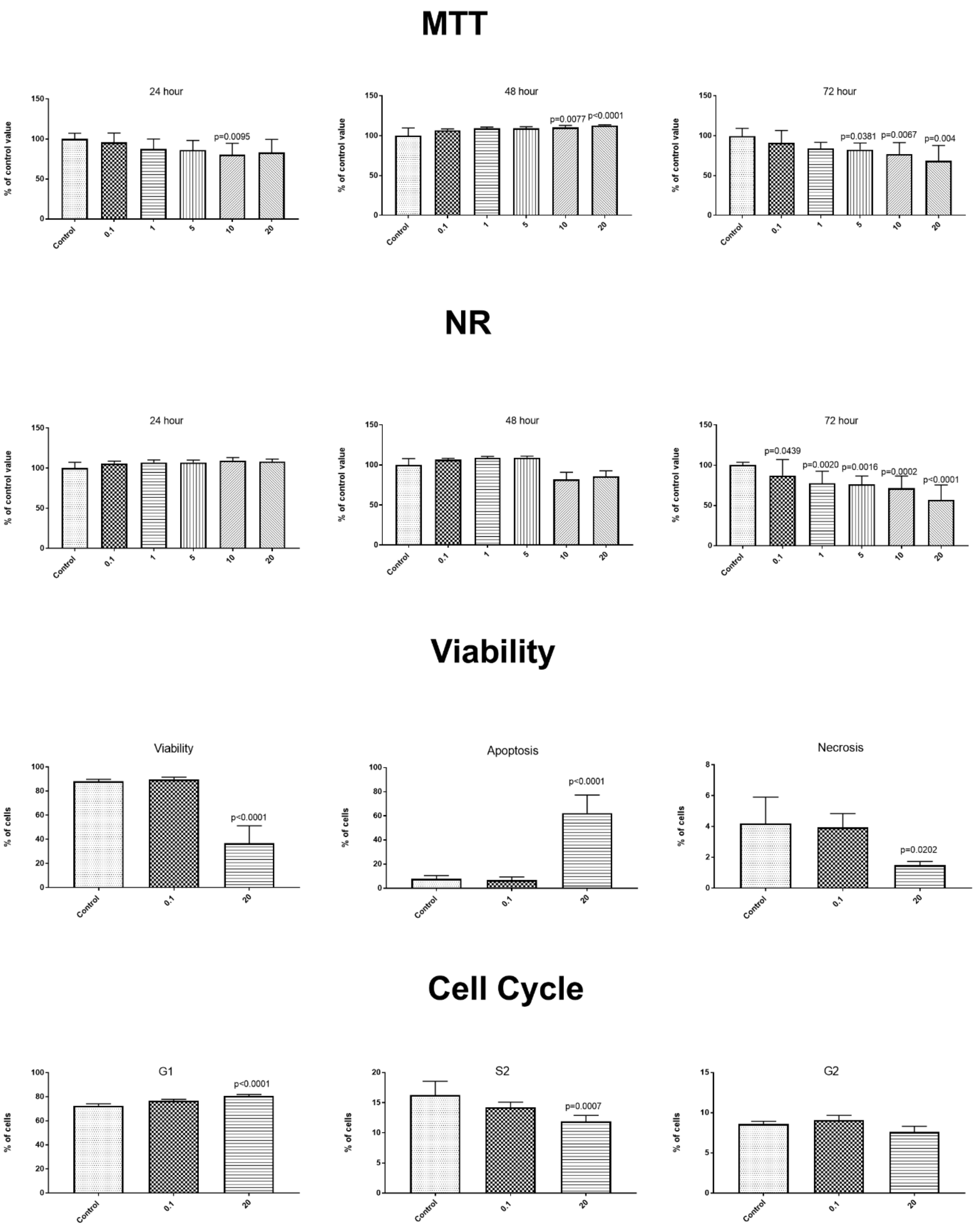

Figure S31. Impact of CM-E (ethanolic extract of control microwave-dried sample) on cytotoxicity (MTT and NR method), type of cell death (Annex V and PI test) and individual phases of the prostate cancer line VCaP – (ATCC® CRL-2876 ™).

MTT

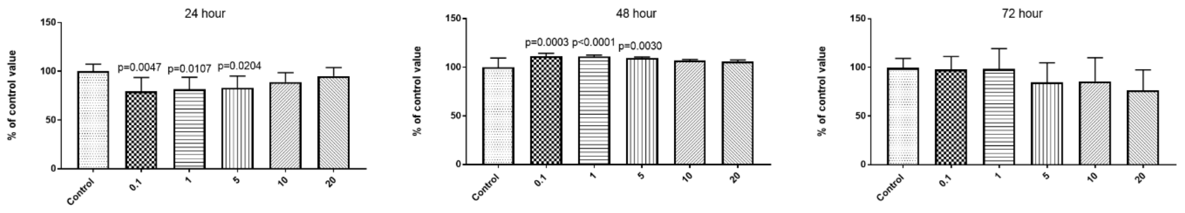

NR

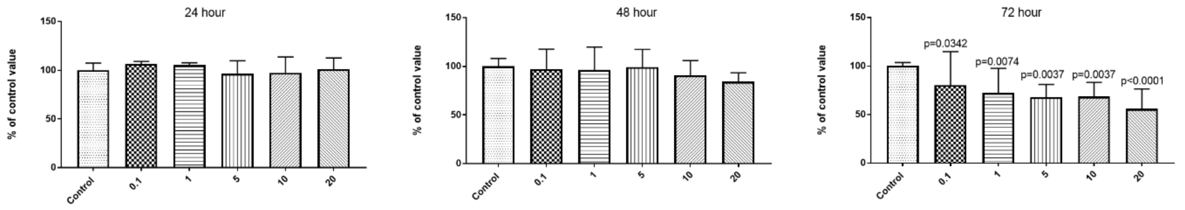

Viability

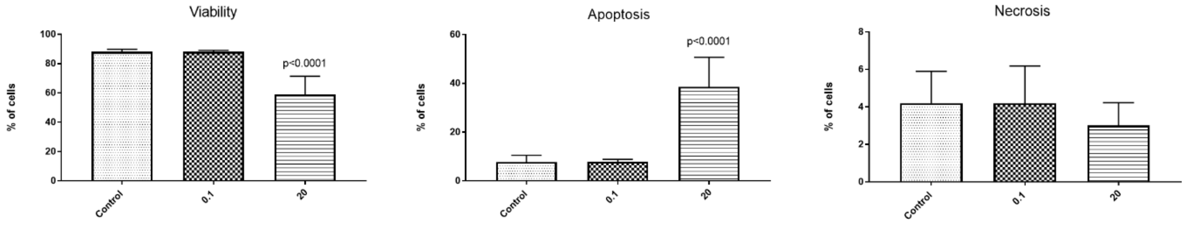

Cell Cycle

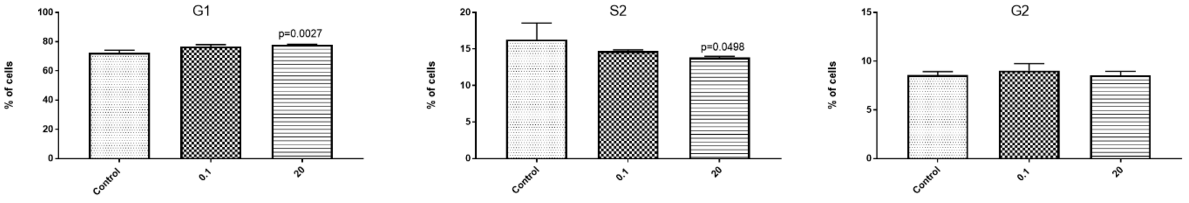

Figure S32. Impact of JAM-E (ethanolic extract of microwave-dried sample from plants elicited with 10 μM of jasmonic acid) on cytotoxicity (MTT and NR method), type of cell death (Annex V and PI test) and individual phases of the prostate cancer line VCaP – (ATCC® CRL-2876 ™).

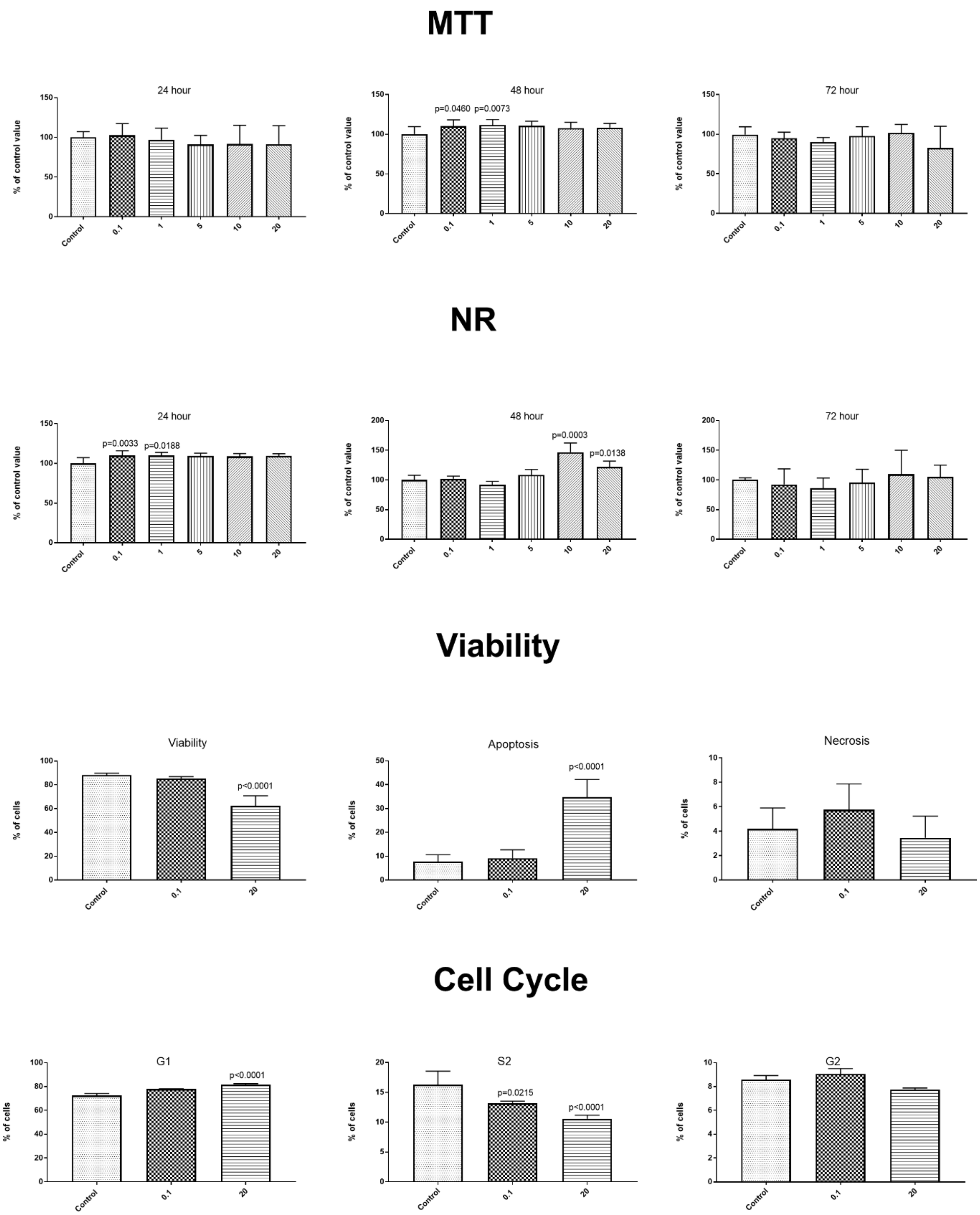

Figure S33. Impact of YEM-E (ethanolic extract of microwave-dried sample from plants elicited with 0.1% yeast extract) on cytotoxicity (MTT and NR method), type of cell death (Annex V and PI test) and individual phases of the prostate cancer line VCaP – (ATCC® CRL-2876 ™).

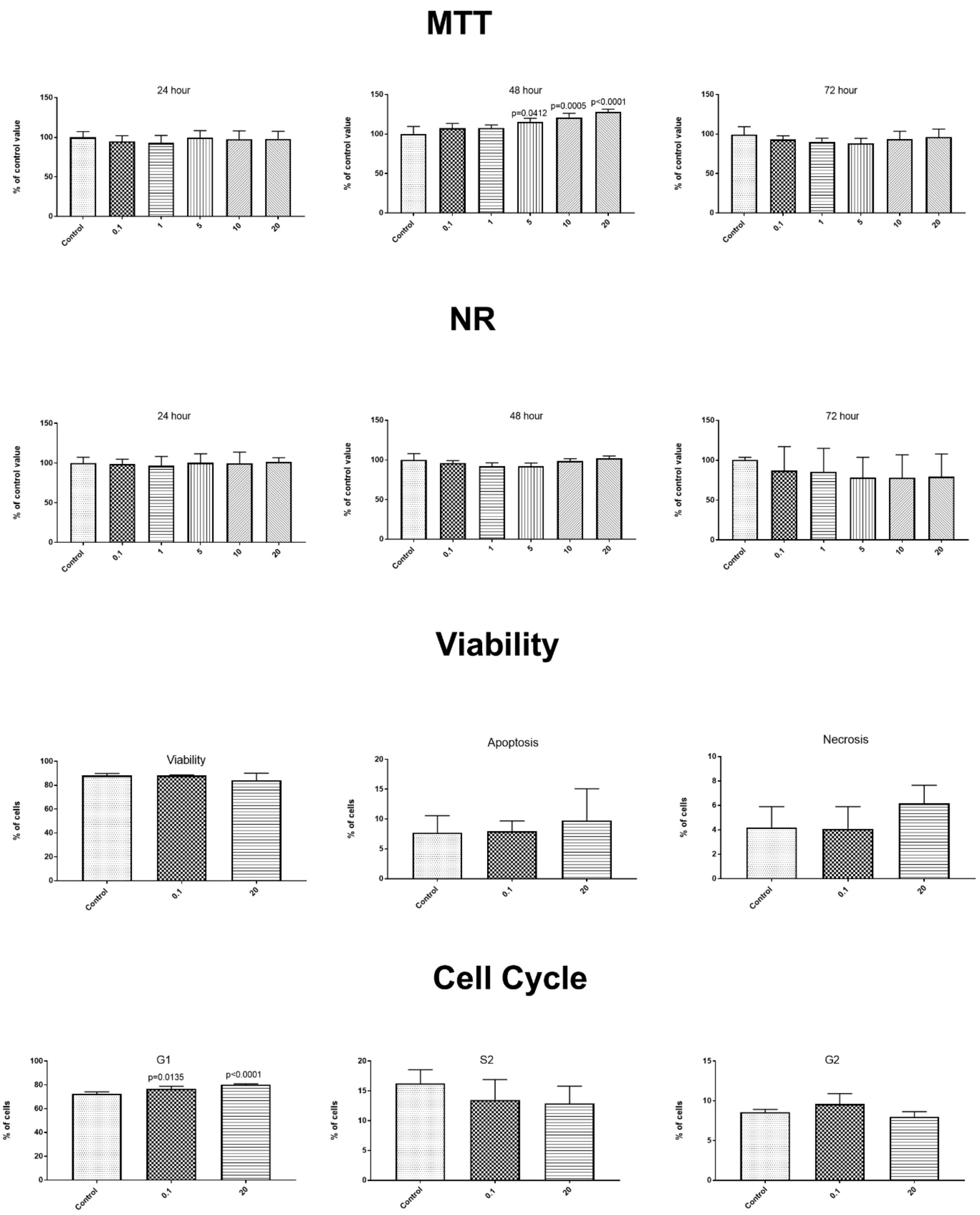

Figure S34. Impact of CM-GD extract (sample after the *in vitro* digestion of control microwave-dried sample) on cytotoxicity (MTT and NR method), type of cell death (Annex V and PI test) and individual phases of the prostate cancer line VCaP – (ATCC® CRL-2876 ™).

MTT

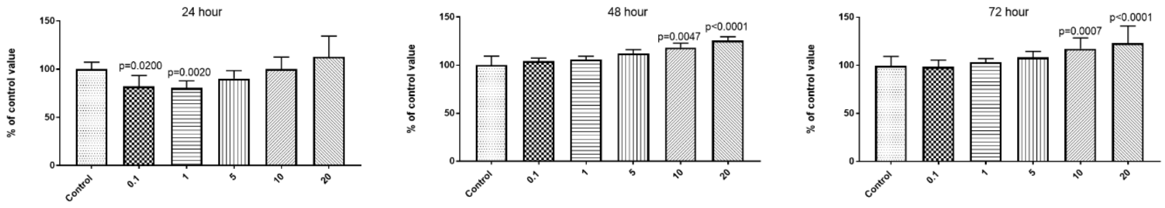

NR

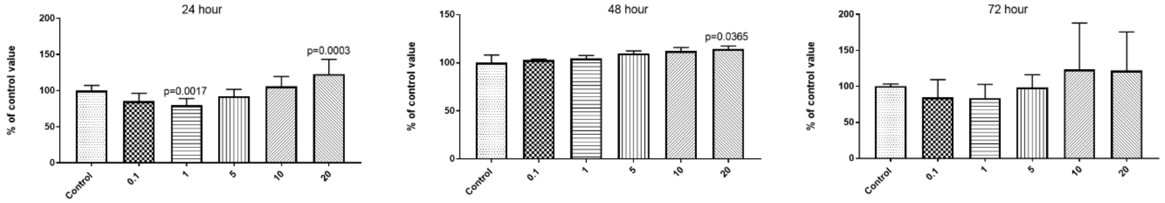

Viability

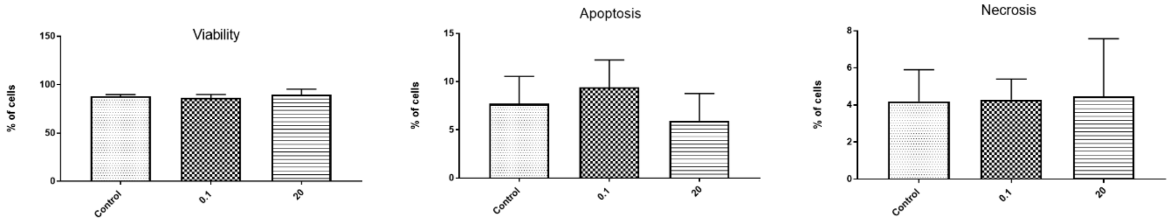

Cell Cycle

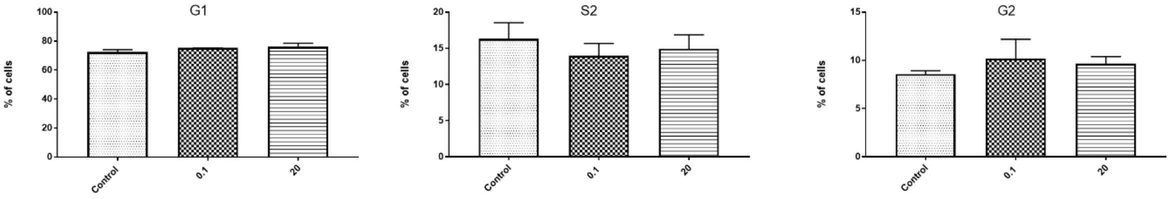

Figure S35. Impact of JAM -GD extract (sample after the *in vitro* digestion of microwave-dried sample from plants elicited with 10 µM of jasmonic acid) on cytotoxicity (MTT and NR method), type of cell death (Annex V and PI test) and individual phases of the prostate cancer line VCaP – (ATCC® CRL-2876 ™).

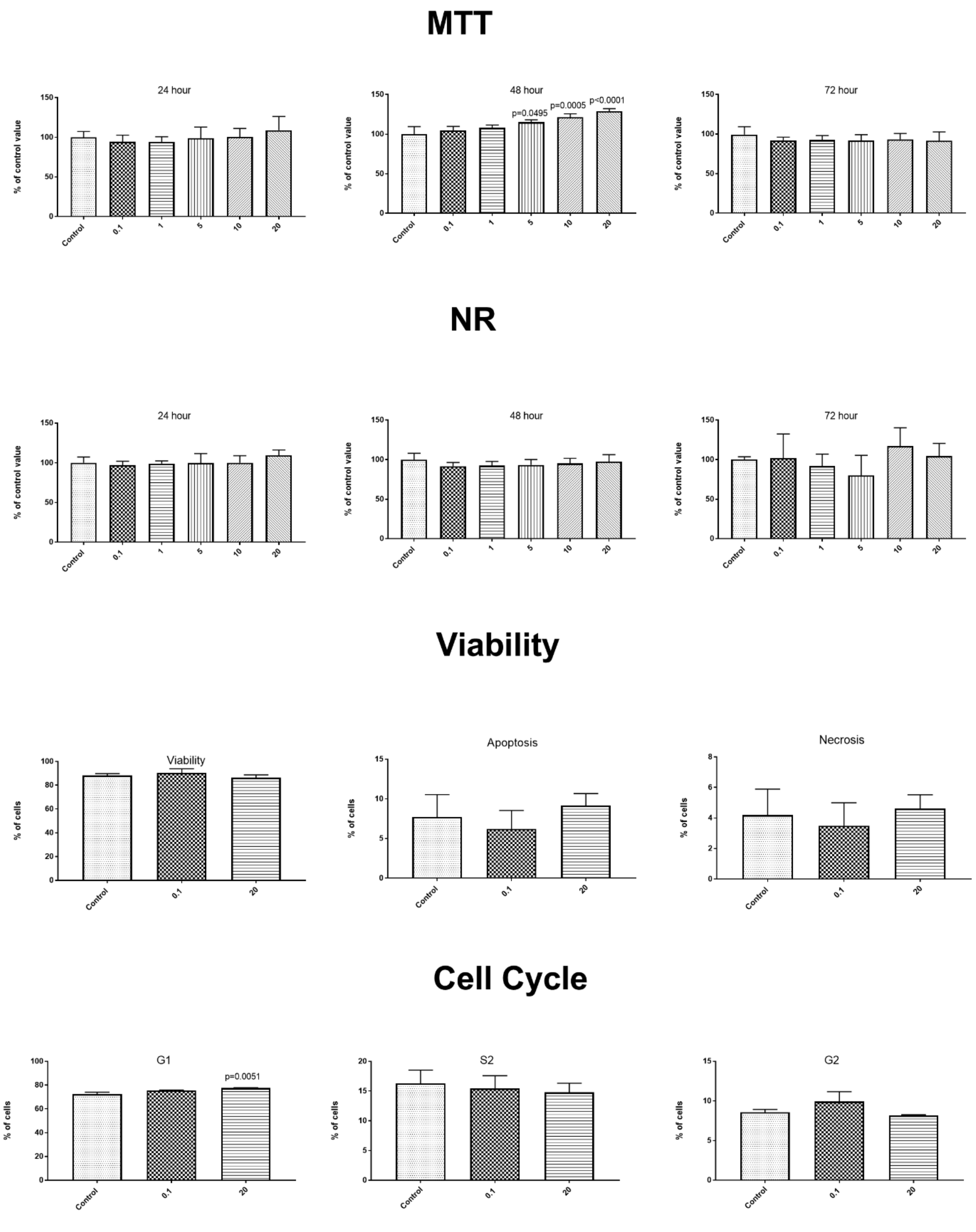

Figure S36. Impact of YEM -GD extract (sample after the *in vitro* digestion of microwave-dried sample from plants elicited with 0.1% yeast extract) on cytotoxicity (MTT and NR method), type of cell death (Annex V and PI test) and individual phases of the prostate cancer line VCaP – (ATCC® CRL-2876 ™).

MTT

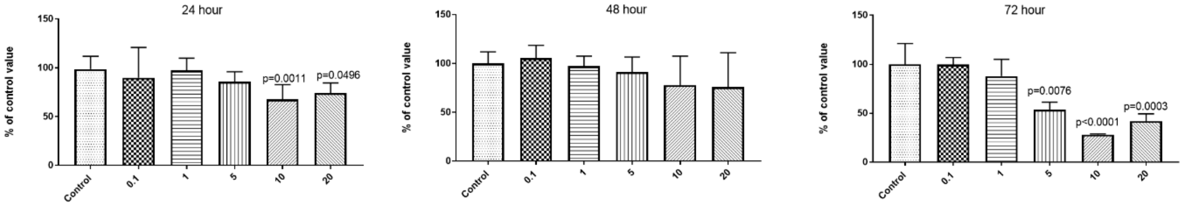

NR

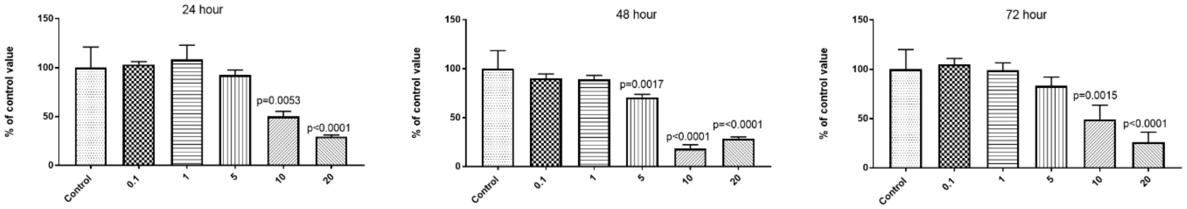

Viability

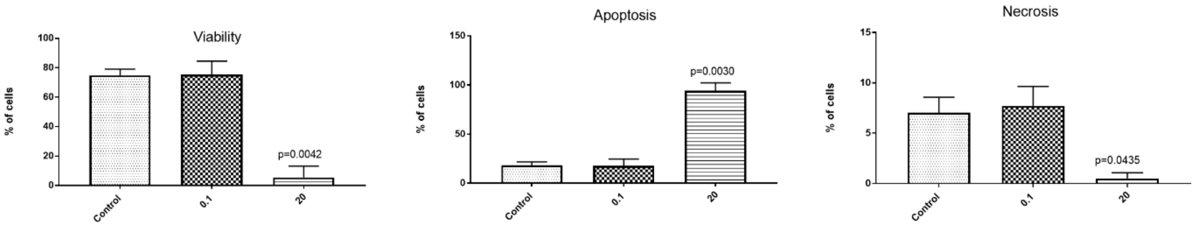

Cell Cycle

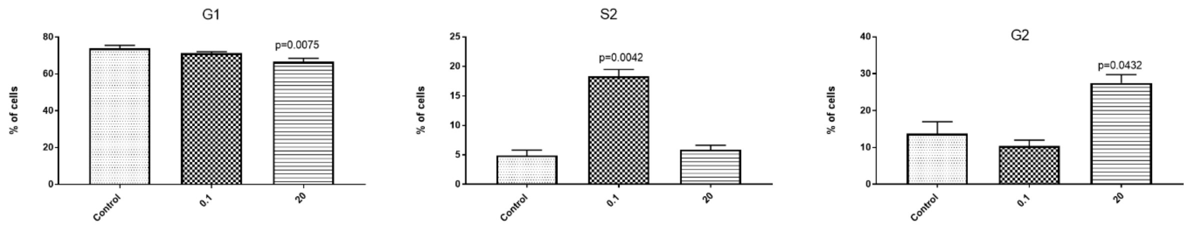

Figure S37. Impact of CT-E (ethanolic extract of control traditionally-dried sample) on cytotoxicity (MTT and NR method), type of cell death (Annex V and PI test) and individual phases of the cell cycle of the healthy prostate epithelial cells (HPrEC ATCC® PCS-440-010 ™ cell line).

MTT

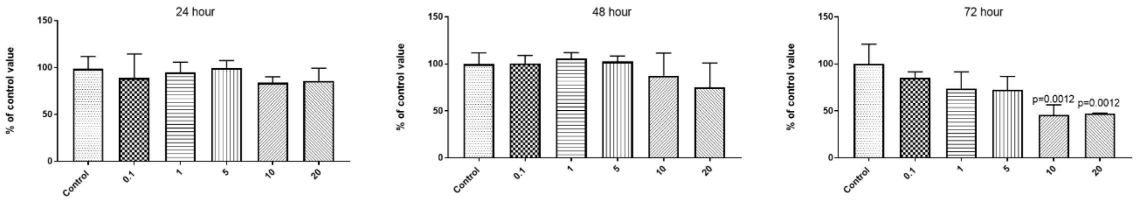

NR

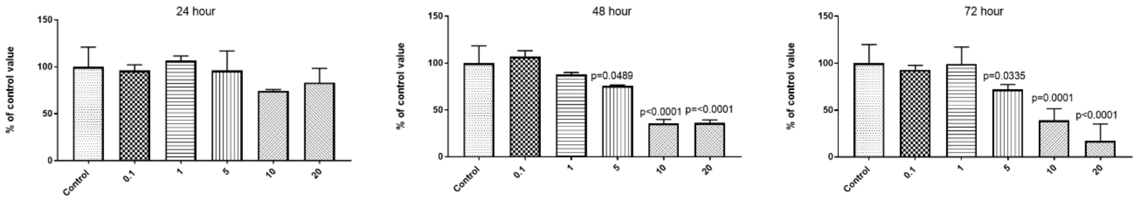

Viability

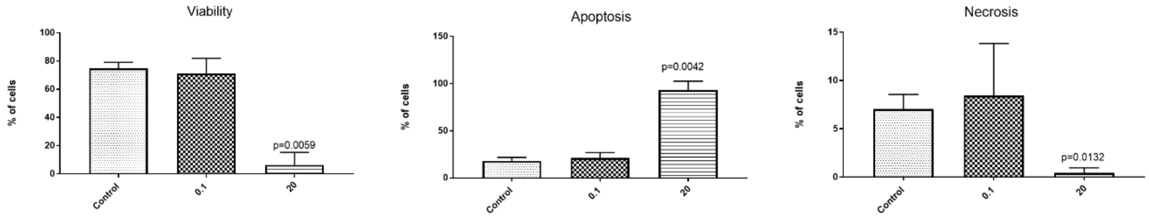

Cell Cycle

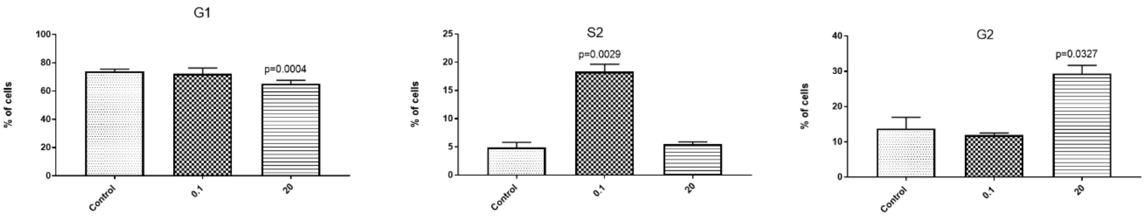

Figure S38. Impact of JAT-E (ethanolic extract of traditionally-dried sample from plants elicited with 10 µM of jasmonic acid) on cytotoxicity (MTT and NR method), type of cell death (Annex V and PI test) and individual phases of the cell cycle of the healthy prostate epithelial cells (HPrEC ATCC® PCS-440-010 ™ cell line).

MTT

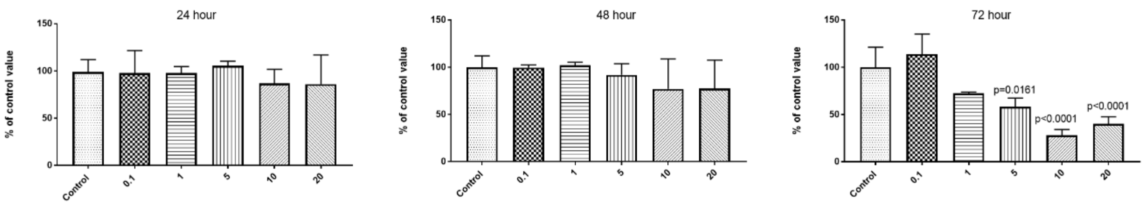

NR

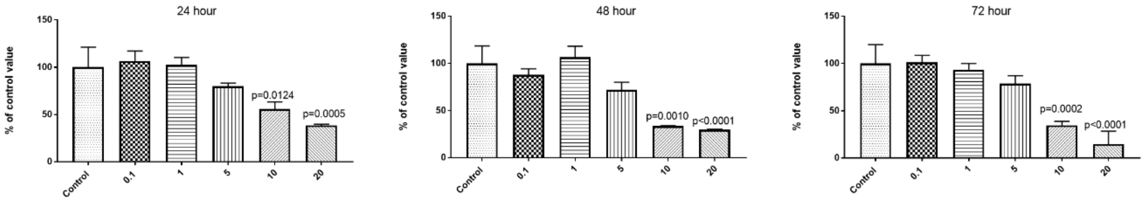

Viability

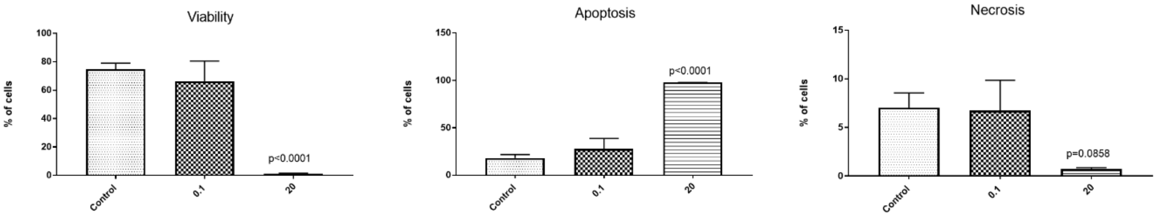

Cell Cycle

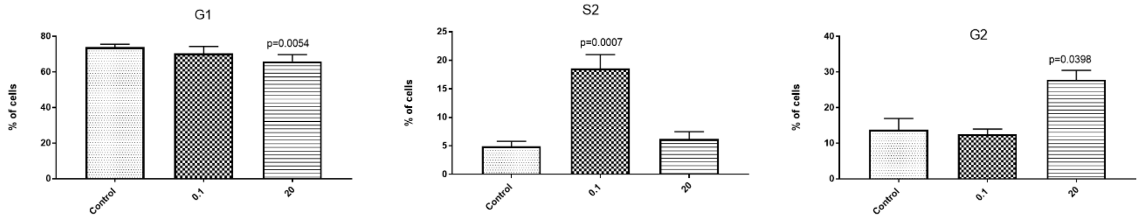

Figure S39. Impact of YET-E (ethanolic extract of traditionally-dried sample from plants elicited with 0.1% yeast extract) on cytotoxicity (MTT and NR method), type of cell death (Annex V and PI test) and individual phases of the cell cycle of the healthy prostate epithelial cells (HPrEC ATCC® PCS-440-010 ™ cell line).

MTT

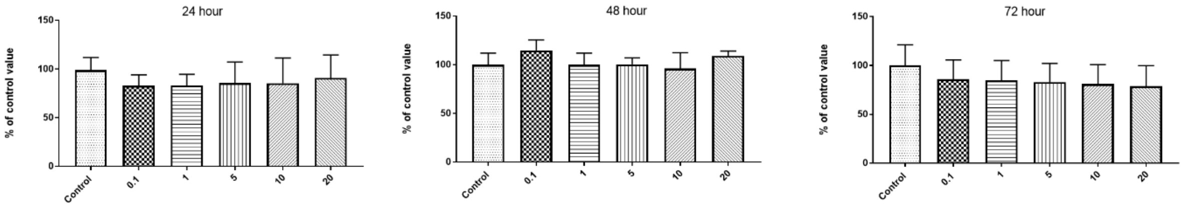

NR

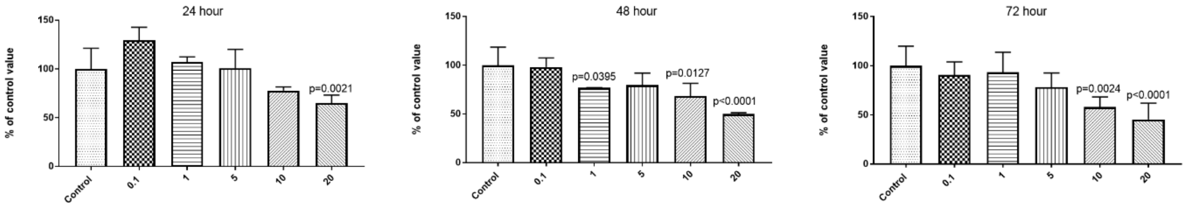

Viability

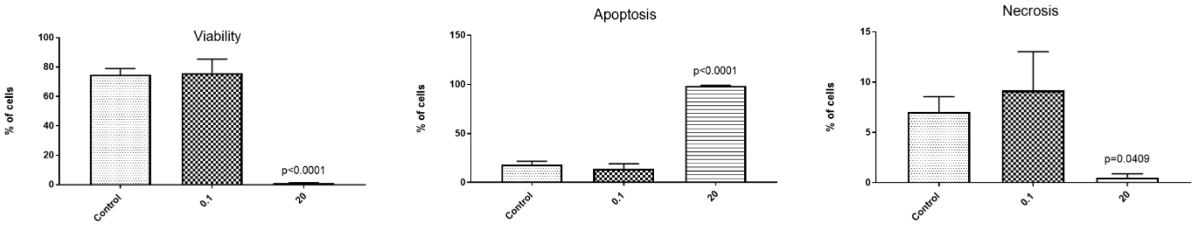

Cell Cycle

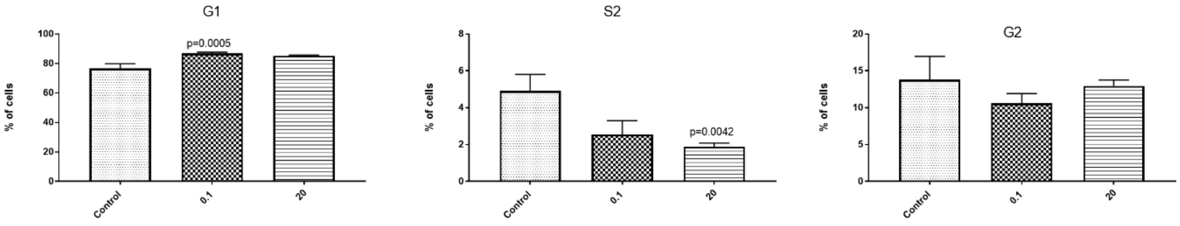

Figure S40. Impact of CT-GD extract (sample after the *in vitro* digestion of control traditionally-dried sample) on cytotoxicity (MTT and NR method), type of cell death (Annex V and PI test) and individual phases of the cell cycle of the healthy prostate epithelial cells (HPrEC ATCC® PCS-440-010 ™ cell line).

MTT

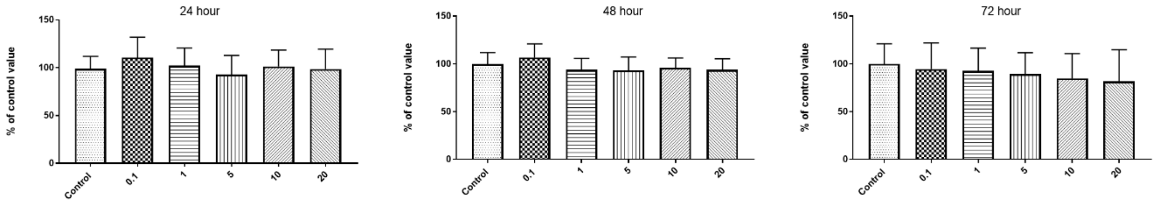

NR

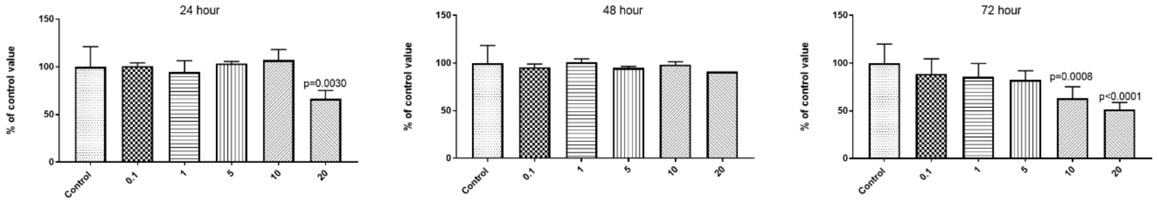

Viability

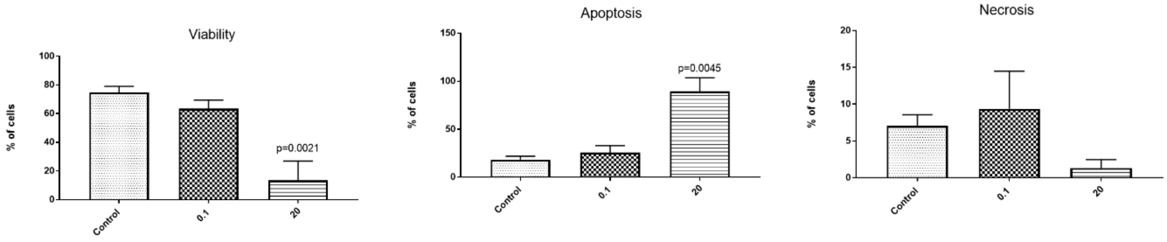

Cell Cycle

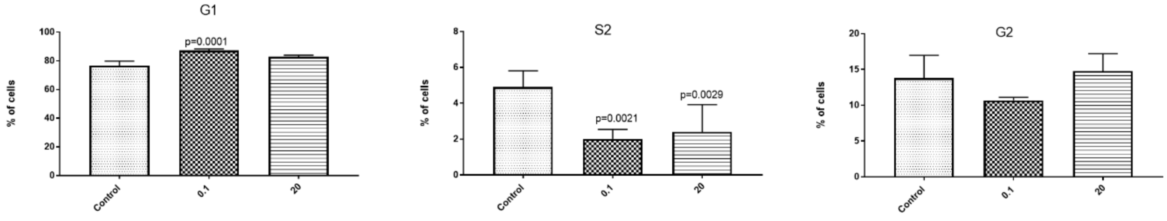

Figure S41. Impact of JAT-GD (sample after the *in vitro* digestion of traditionally-dried sample from plants elicited with 10 µM of jasmonic acid) on cytotoxicity (MTT and NR method), type of cell death (Annex V and PI test) and individual phases of the cell cycle of the healthy prostate epithelial cells (HPrEC ATCC® PCS-440-010 ™ cell line).

MTT

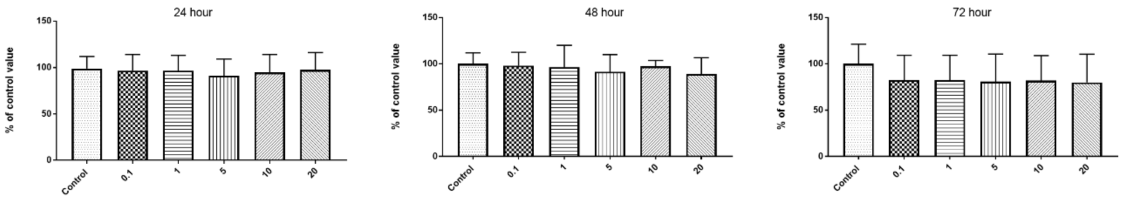

NR

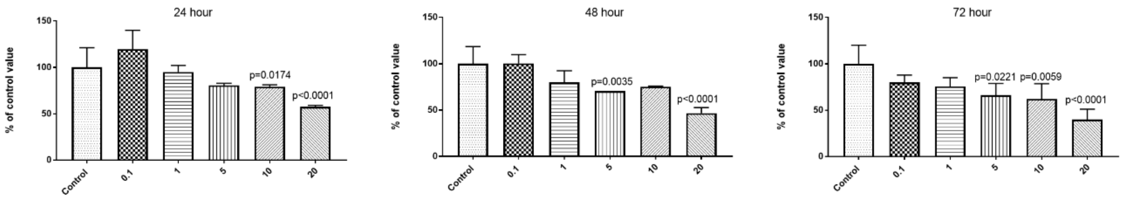

Viability

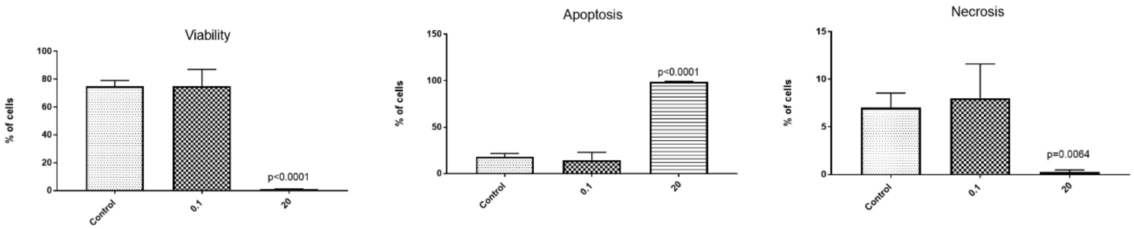

Cell Cycle

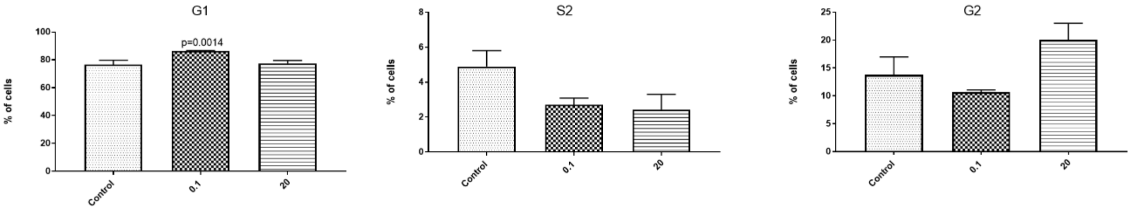

Figure S42. Impact of YET -GD extract (sample after the *in vitro* digestion of traditionally-dried sample from plants elicited with 0.1% yeast extract) on cytotoxicity (MTT and NR method), type of cell death (Annex V and PI test) and individual phases of the cell cycle of the healthy prostate epithelial cells (HPrEC ATCC® PCS-440-010 ™ cell line).

MTT

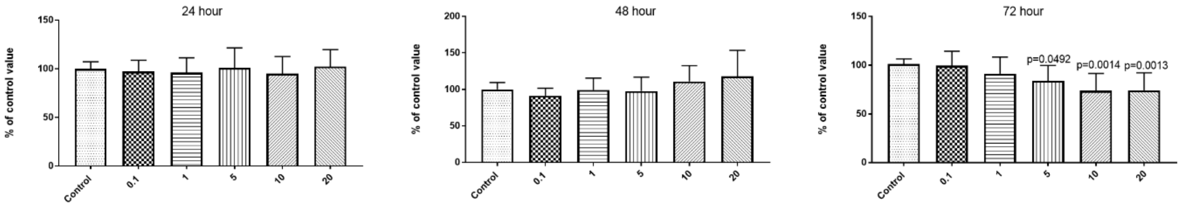

NR

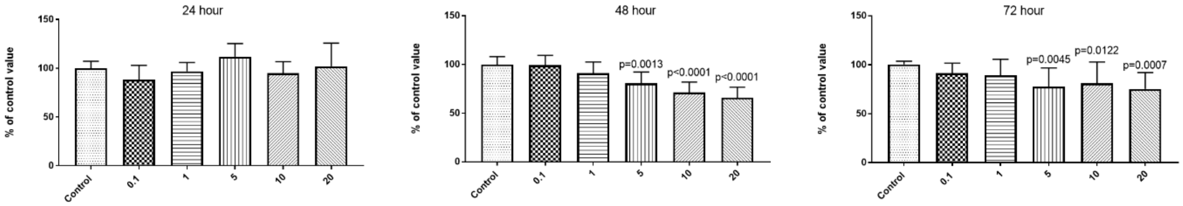

Viability

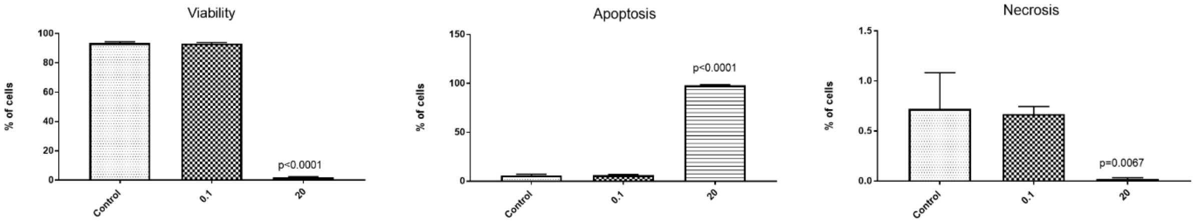

Cell Cycle

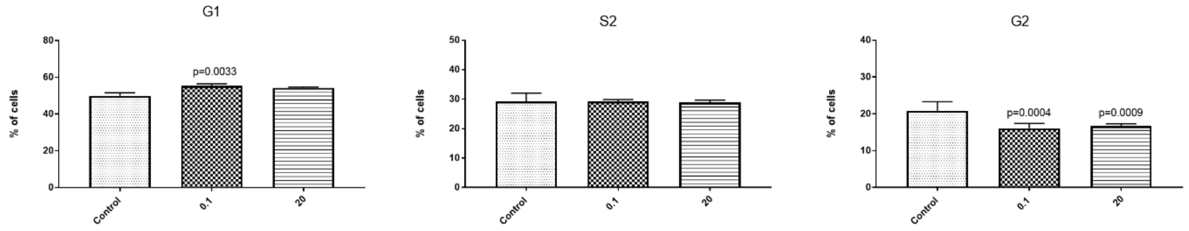

Figure S43. Impact of CT-E (ethanolic extract of control traditionally-dried sample) on cytotoxicity (MTT and NR method), type of cell death (Annex V and PI test) and individual phases of the cancer gastric epithelial NCI-N87 (ATCC® CRL5822 ™) cell line.

MTT

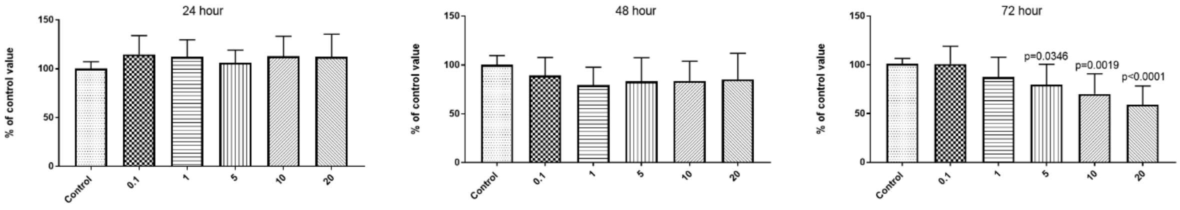

NR

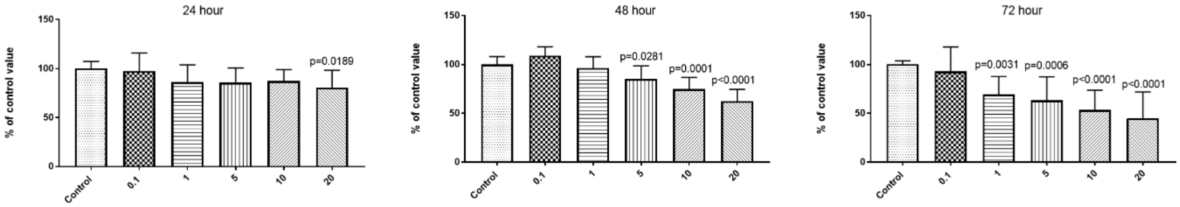

Viability

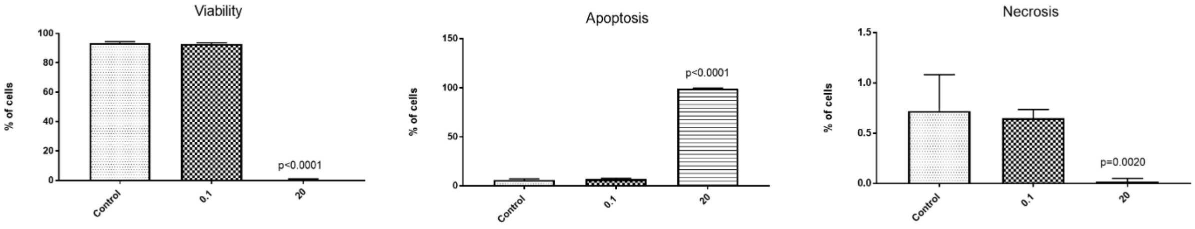

Cell Cycle

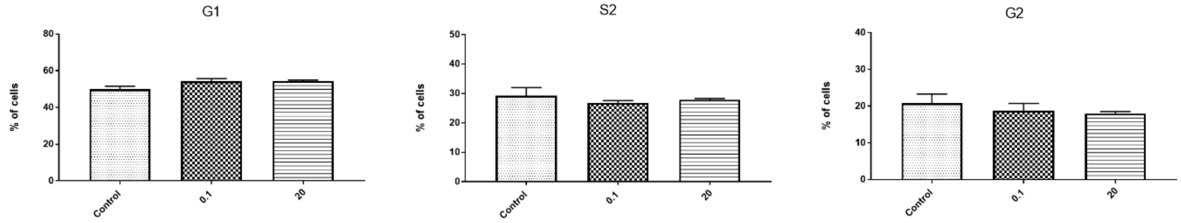

Figure S44. Impact of JAT-E (ethanolic extract of traditionally-dried sample from plants elicited with 10 μM of jasmonic acid) on cytotoxicity (MTT and NR method), type of cell death (Annex V and PI test) and individual phases of the cancer gastric epithelial NCI-N87 (ATCC® CRL5822 ™) cell line.

MTT

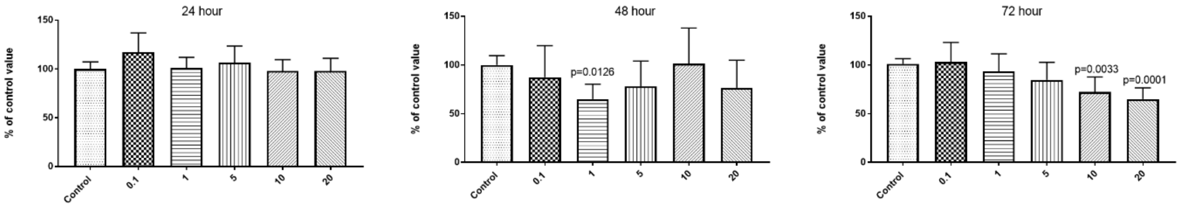

NR

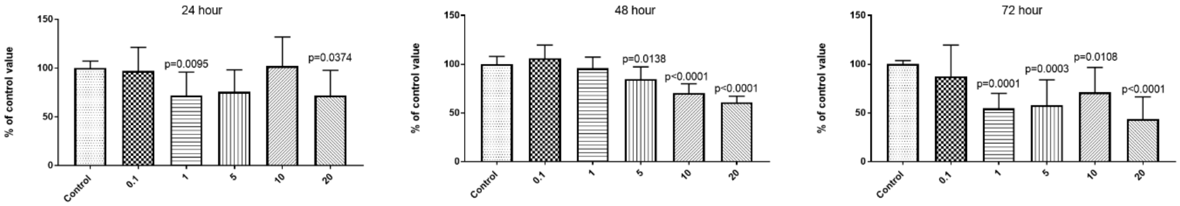

Viability

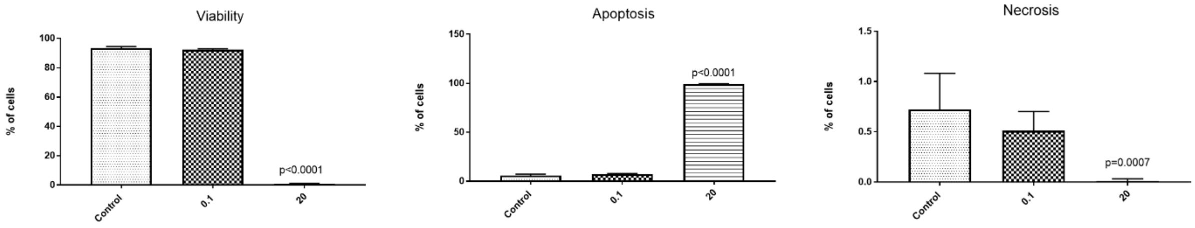

Cell Cycle

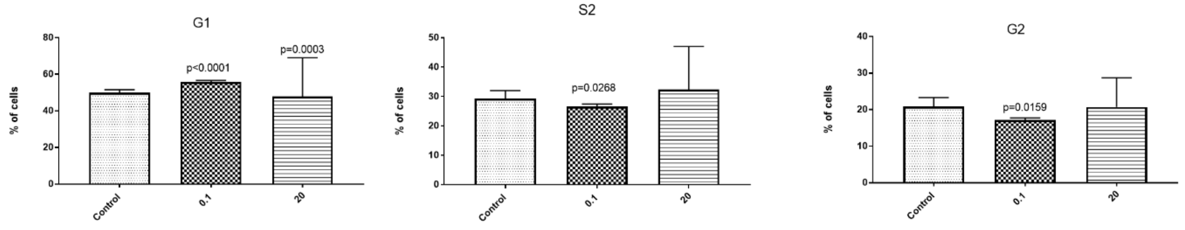

Figure S45. Impact of YET-E (ethanolic extract of traditionally-dried sample from plants elicited with 0.1% yeast extract) on cytotoxicity (MTT and NR method), type of cell death (Annex V and PI test) and individual phases of the cancer gastric epithelial NCI-N87 (ATCC® CRL5822 ™) cell line.

MTT

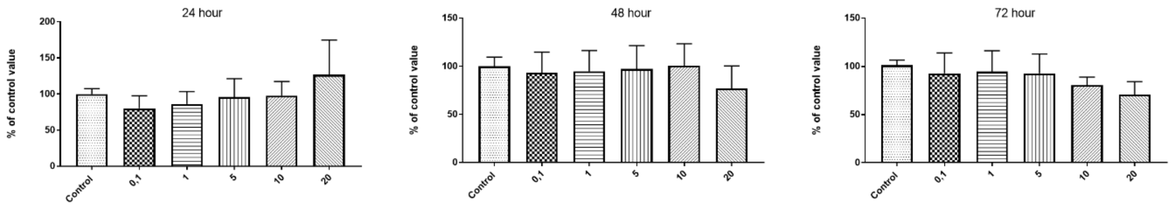

NR

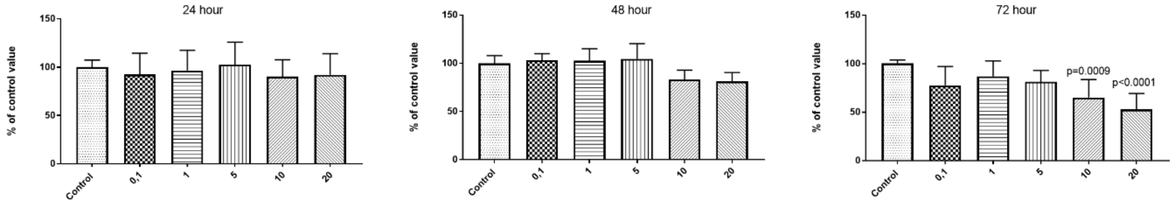

Viability

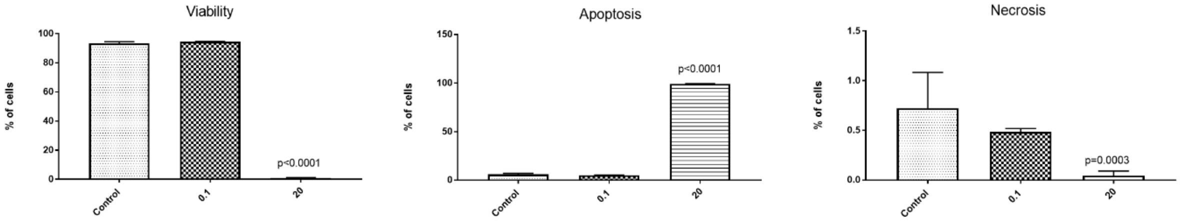

Cell Cycle

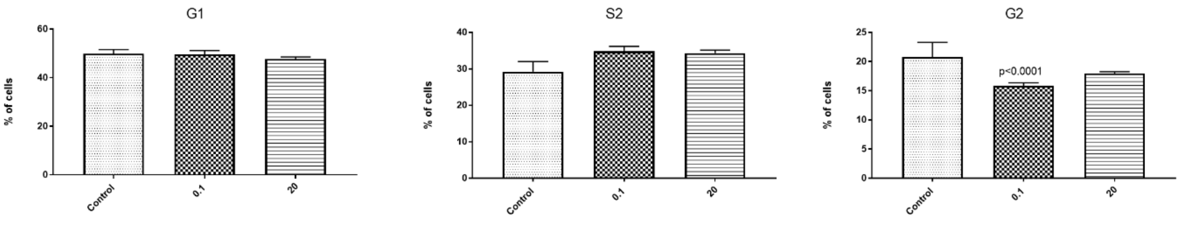

Figure S46. Impact of CT -GD extract (sample after the *in vitro* digestion of control traditionally-dried sample) on cytotoxicity (MTT and NR method), type of cell death (Annex V and PI test) and individual phases of the cancer gastric epithelial NCI-N87 (ATCC® CRL5822 ™) cell line.

MTT

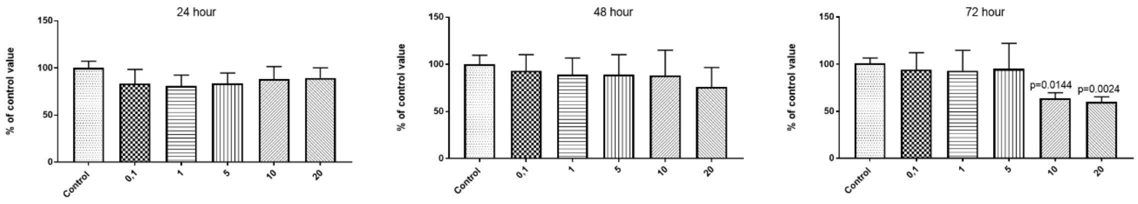

NR

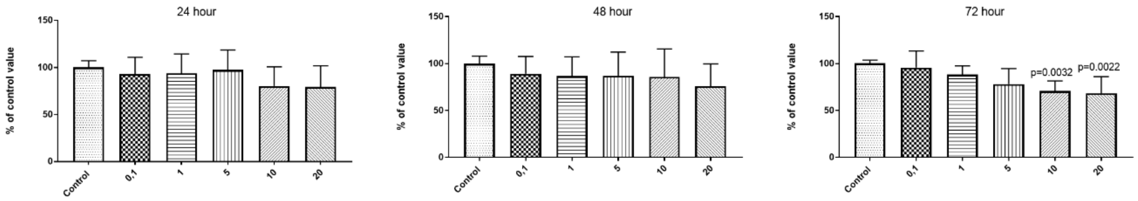

Viability

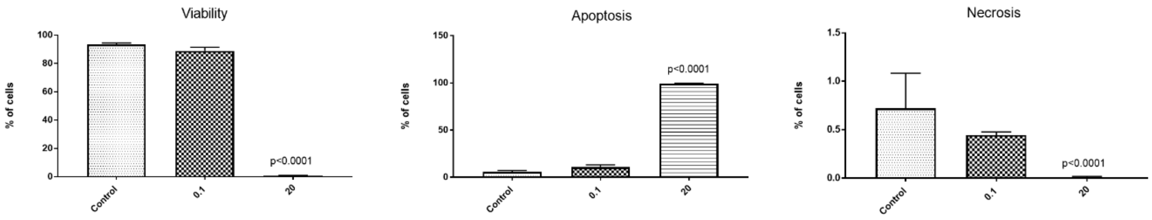

Cell Cycle

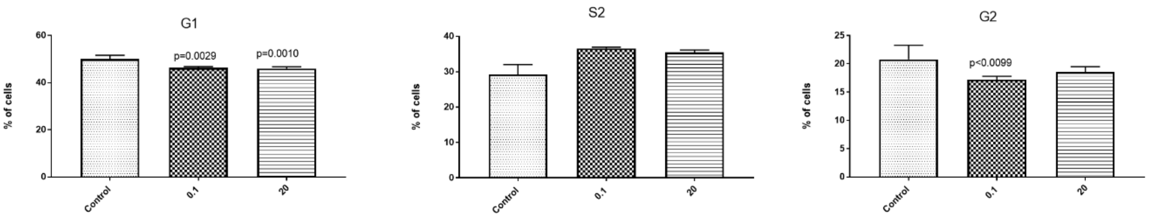

Figure S47. Impact of JAT -GD extract (sample after the *in vitro* digestion of traditionally-dried sample from plants elicited with 10 µM of jasmonic acid) on cytotoxicity (MTT and NR method), type of cell death (Annex V and PI test) and individual phases of the cancer gastric epithelial NCI-N87 (ATCC® CRL5822 ™) cell line.

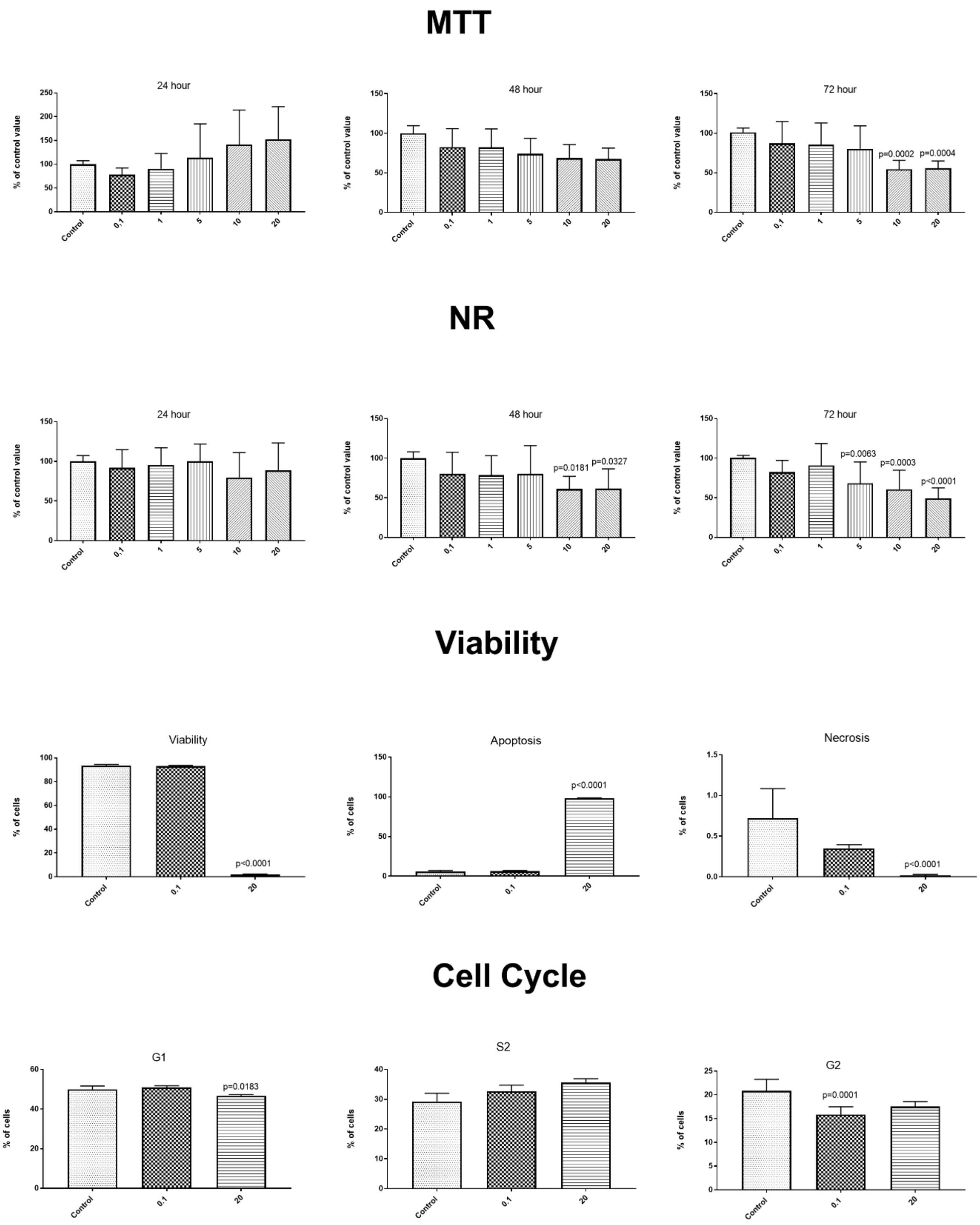

Figure S48. Impact of YET -GD extract (sample after the *in vitro* digestion of traditionally-dried sample from plants elicited with 0.1% yeast extract) on cytotoxicity (MTT and NR method), type of cell death (Annex V and PI test) and individual phases of the cancer gastric epithelial NCI-N87 (ATCC® CRL5822 ™) cell line.

MTT

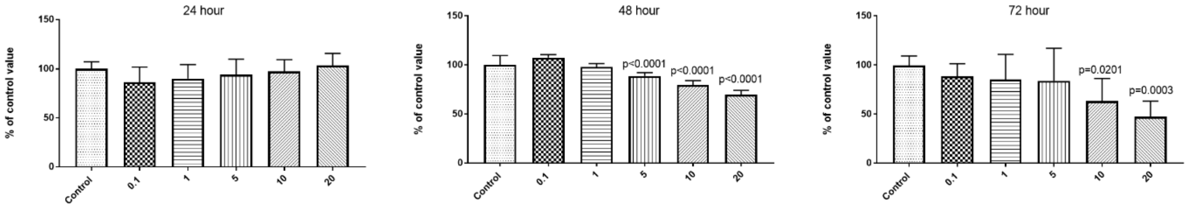

NR

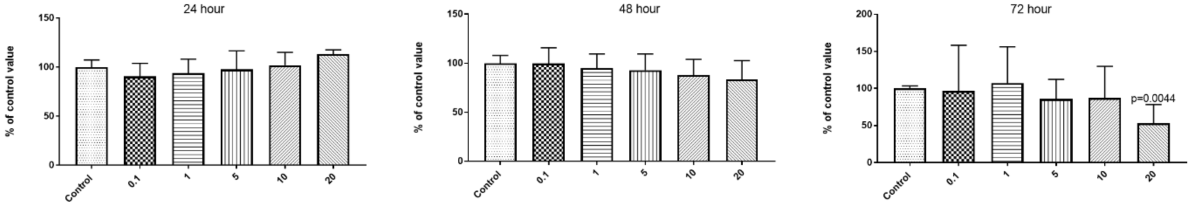

Viability

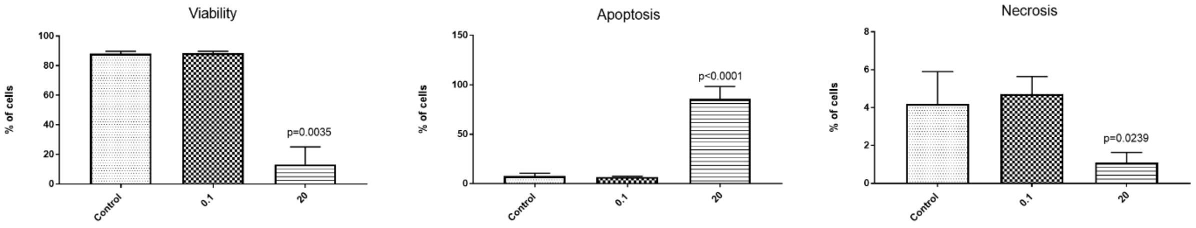

Cell Cycle

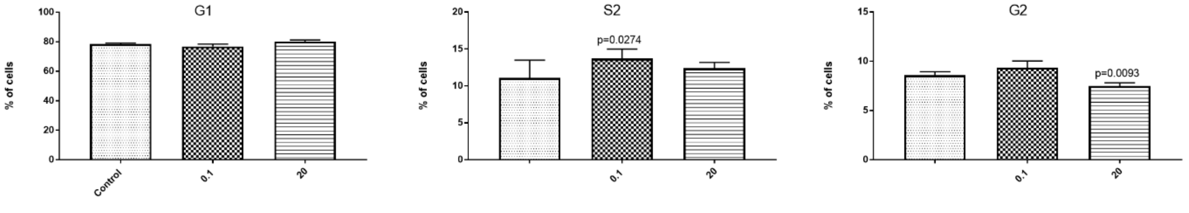

Figure S49. Impact of CT-E (ethanolic extract of control traditionally-dried sample) on cytotoxicity (MTT and NR method), type of cell death (Annex V and PI test) and individual phases of the prostate cancer line VCaP – (ATCC® CRL-2876 ™).

MTT

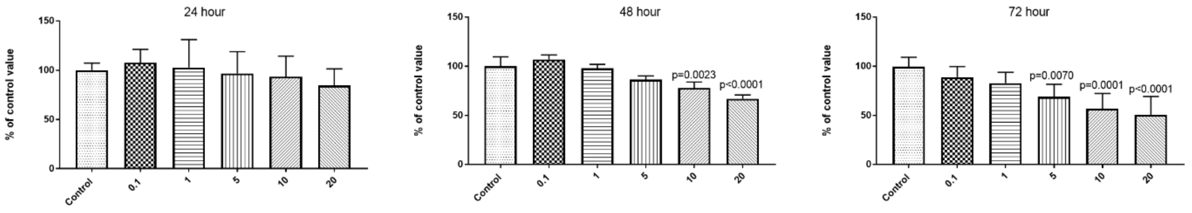

NR

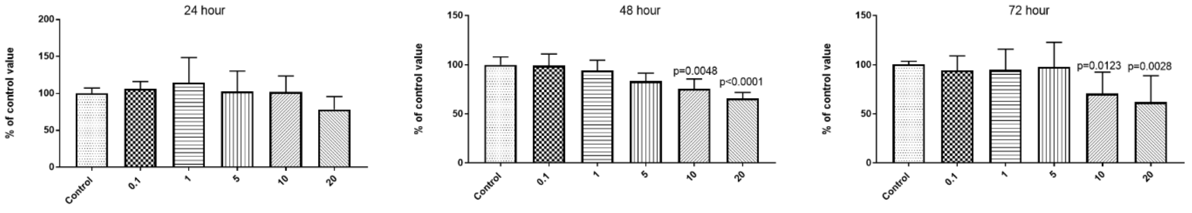

Viability

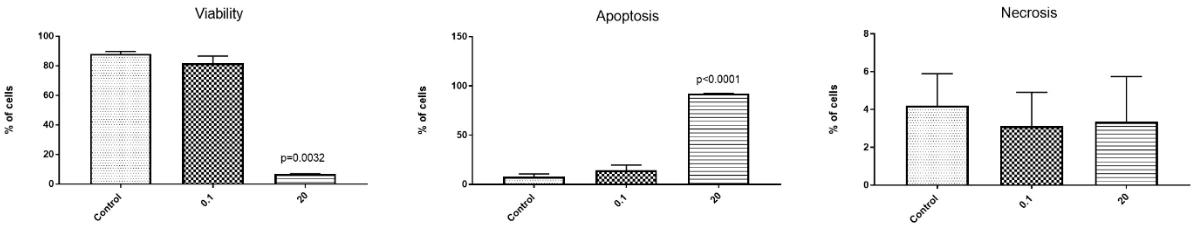

Cell Cycle

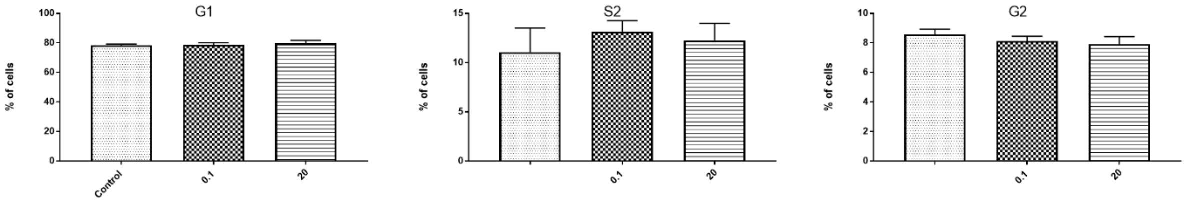

Figure S50. Impact of JAT-E (ethanolic extract of traditionally-dried sample from plants elicited with 10 µM of jasmonic acid) on cytotoxicity (MTT and NR method), type of cell death (Annex V and PI test) and individual phases of the prostate cancer line VCaP – (ATCC® CRL-2876 ™).

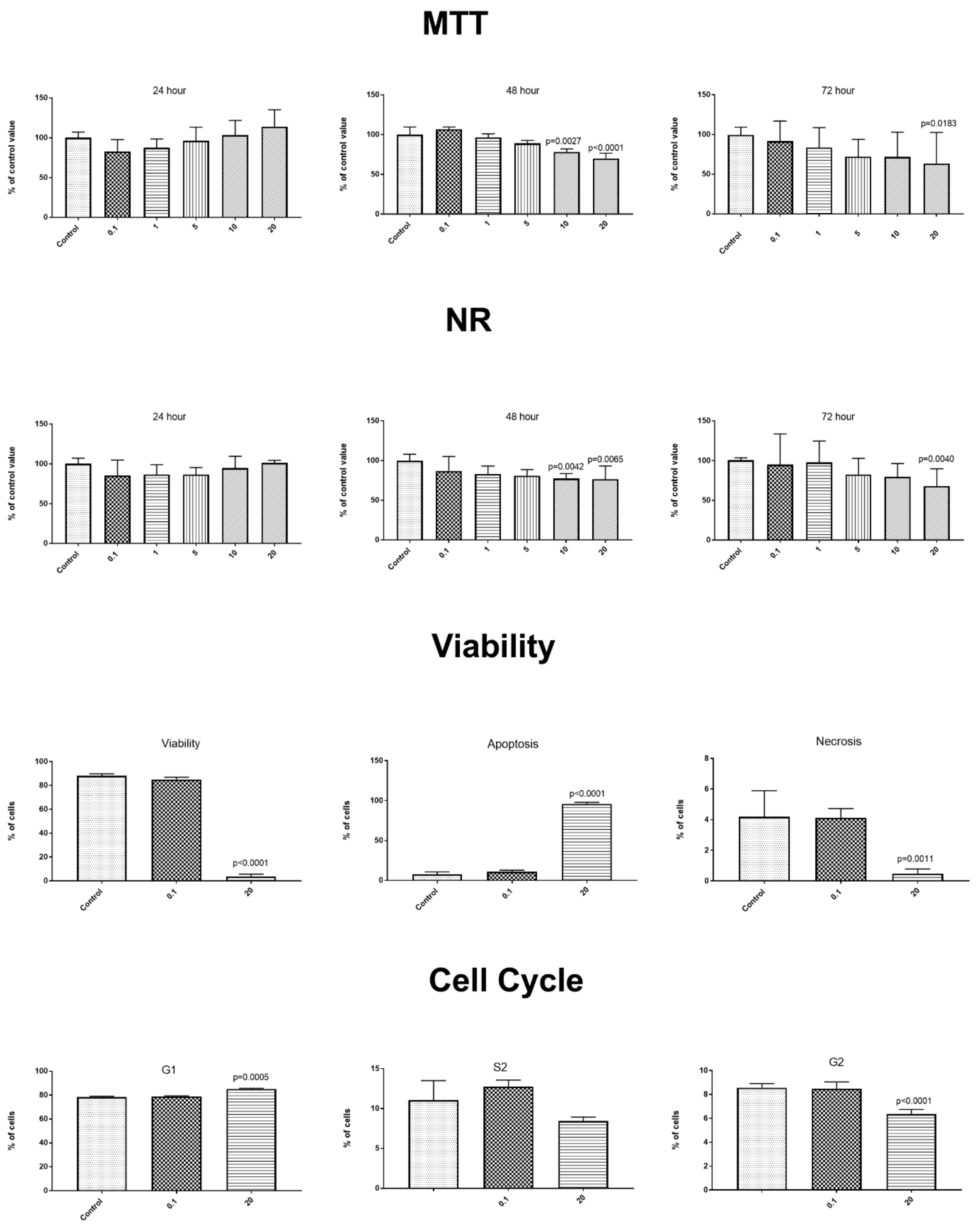

Figure S51. Impact of YET-E (ethanolic extract of traditionally-dried sample from plants elicited with 0.1% yeast extract) on cytotoxicity (MTT and NR method), type of cell death (Annex V and PI test) and individual phases of the prostate cancer line VCaP – (ATCC® CRL-2876 ™).

MTT

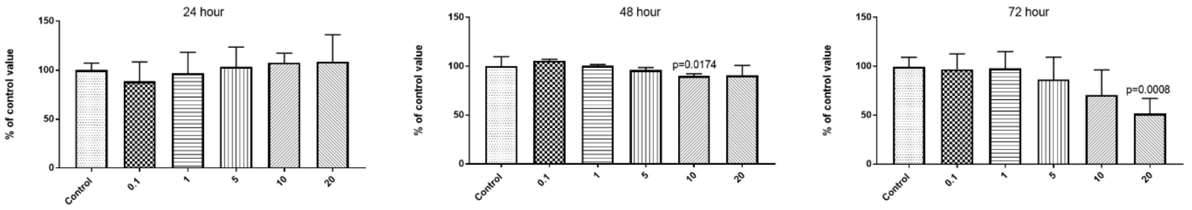

NR

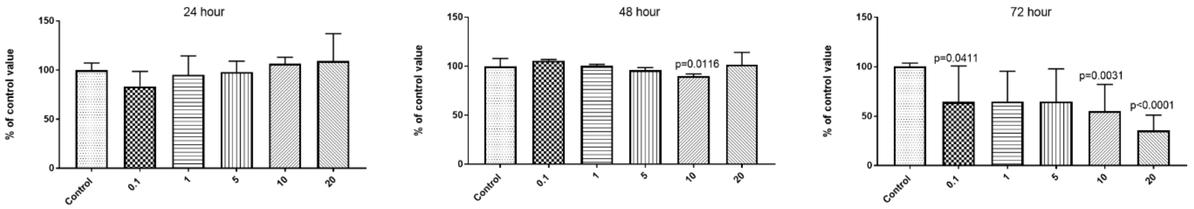

Viability

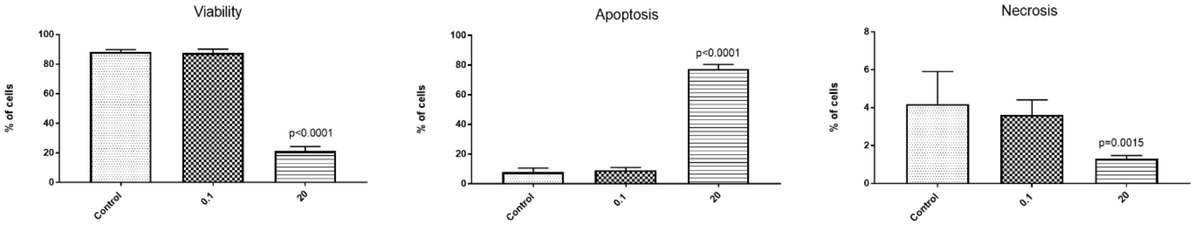

Cell Cycle

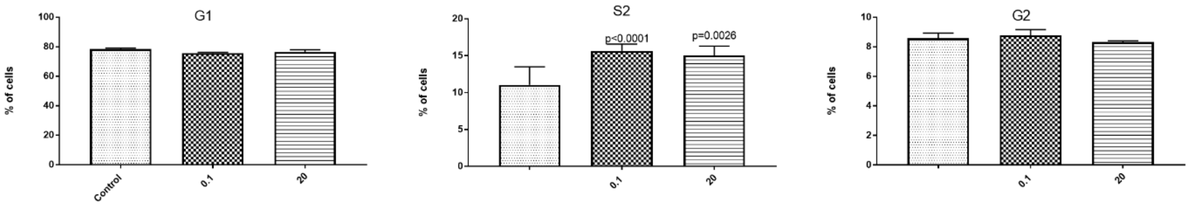

Figure S52. Impact of CT-GD extract (sample after the *in vitro* digestion of control traditionally-dried sample) on cytotoxicity (MTT and NR method), type of cell death (Annex V and PI test) and individual phases of the prostate cancer line VCaP – (ATCC® CRL-2876 ™).

MTT

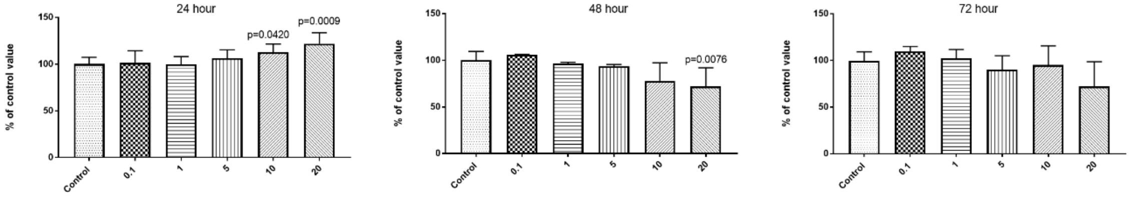

NR

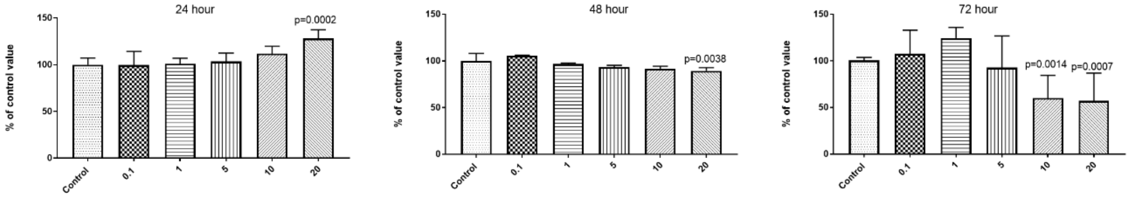

Viability

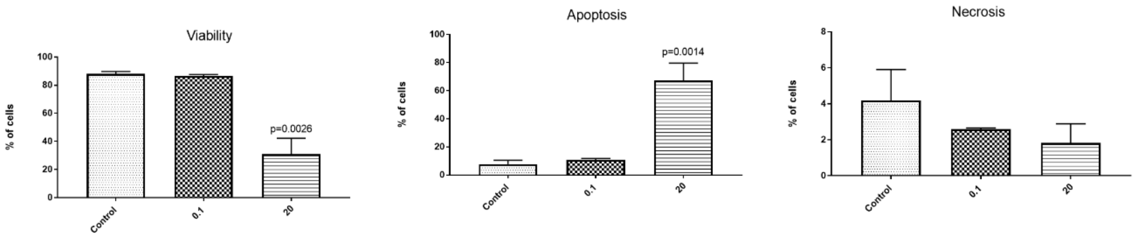

Cell Cycle

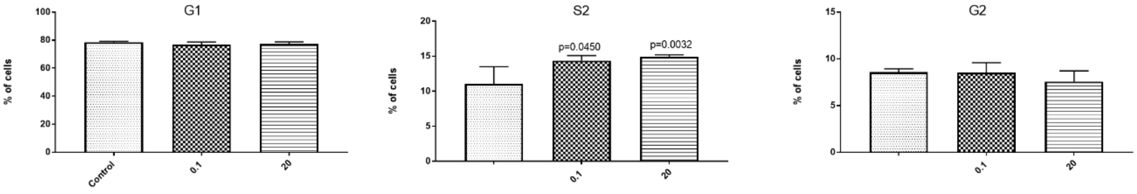

Figure S53. Impact of JAT -GD extract (sample after the *in vitro* digestion of traditionally-dried sample from plants elicited with 10 µM of jasmonic acid) on cytotoxicity (MTT and NR method), type of cell death (Annex V and PI test) and individual phases of the prostate cancer line VCaP – (ATCC® CRL-2876 ™).

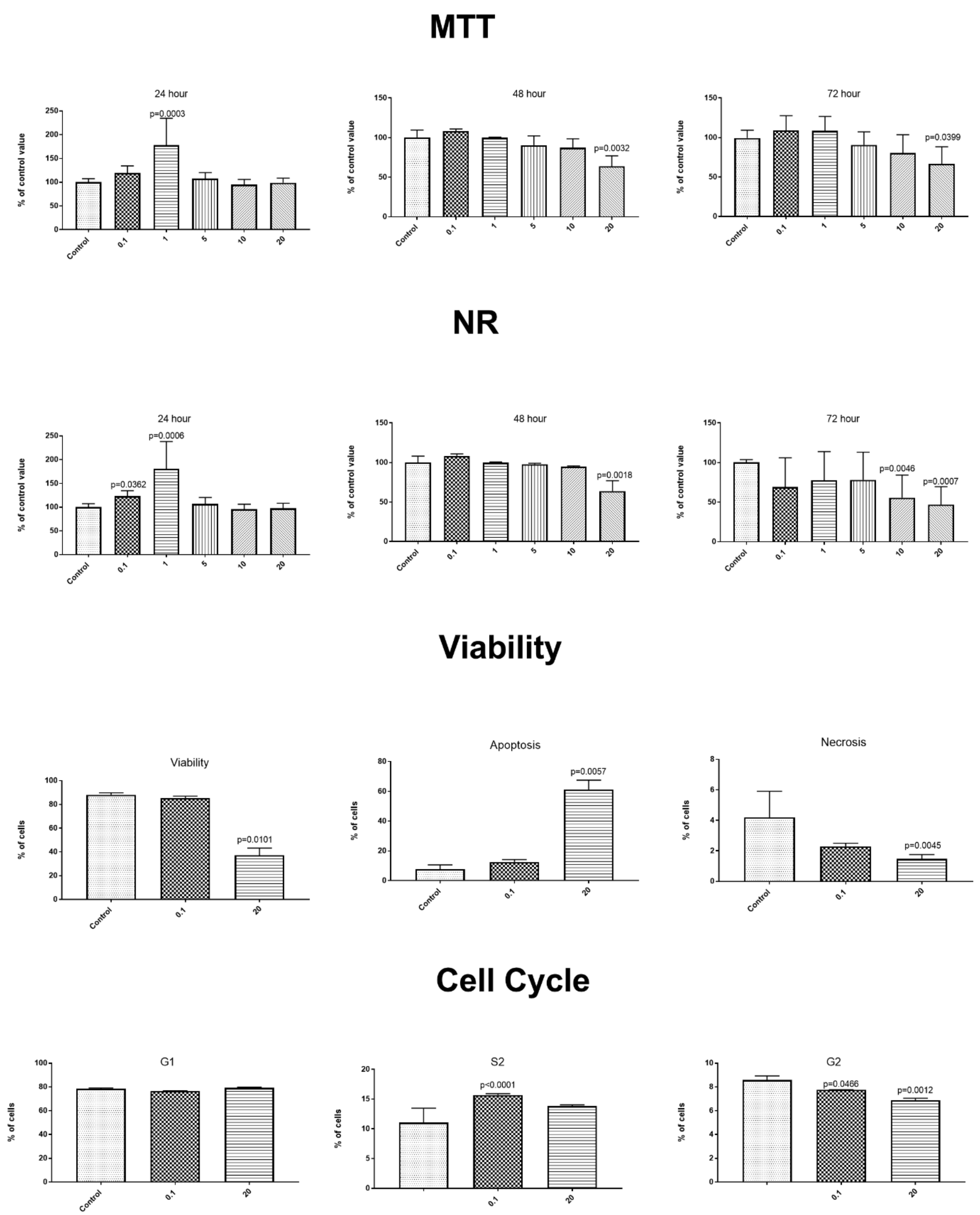

Figure S54. Impact of YET -GD extract (sample after the *in vitro* digestion of traditionally-dried sample from plants elicited with 0.1% yeast extract) on cytotoxicity (MTT and NR method), type of cell death (Annex V and PI test) and individual phases of the prostate cancer line VCaP – (ATCC® CRL-2876 ™).

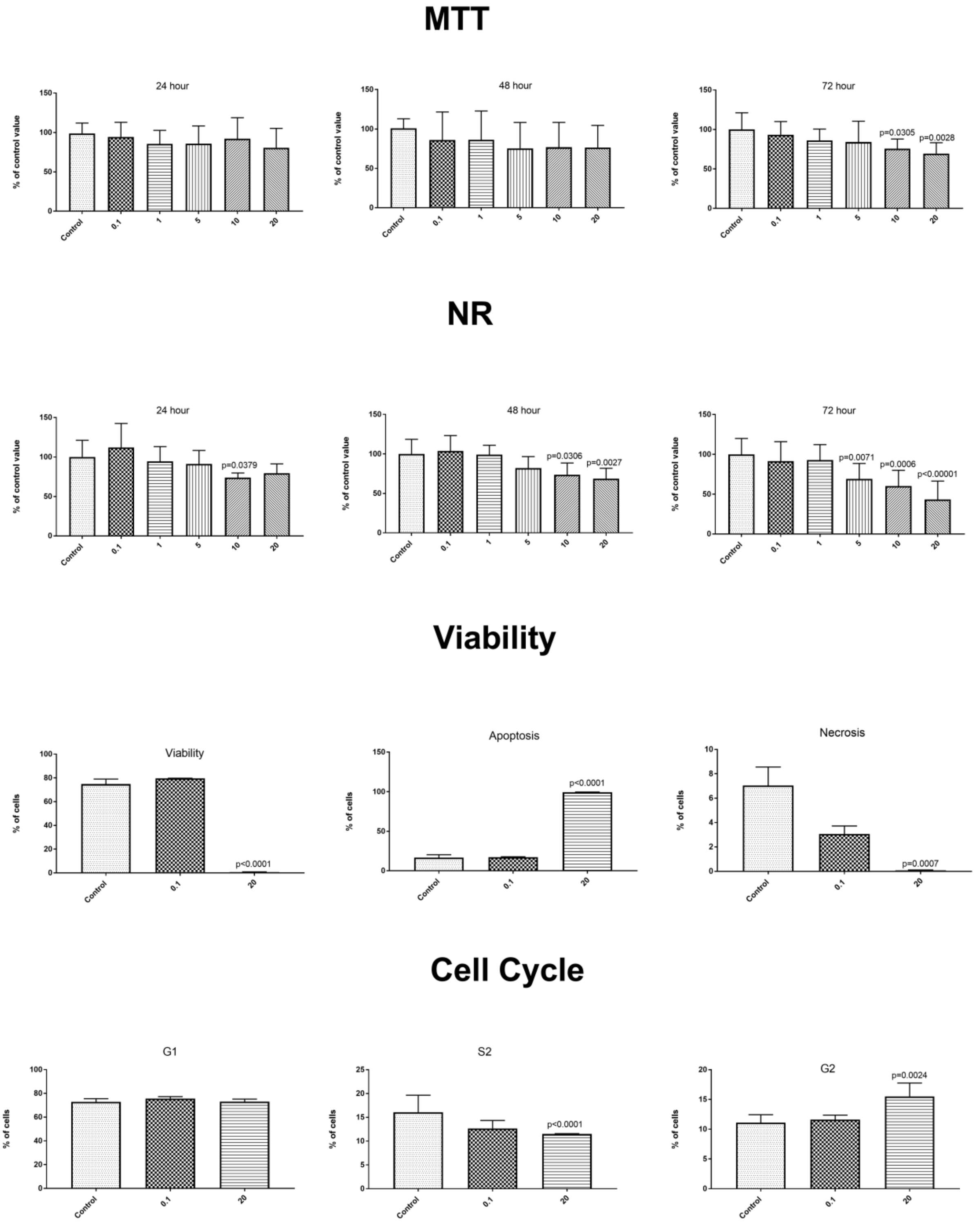

Figure S55. Impact of CC-E (ethanolic extract of control convectionally-dried sample) on cytotoxicity (MTT and NR method), type of cell death (Annex V and PI test) and individual phases of the cell cycle of the healthy prostate epithelial cells (HPrEC ATCC® PCS-440-010 ™ cell line).

MTT

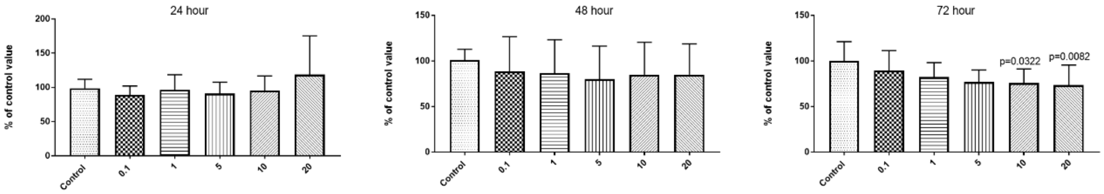

NR

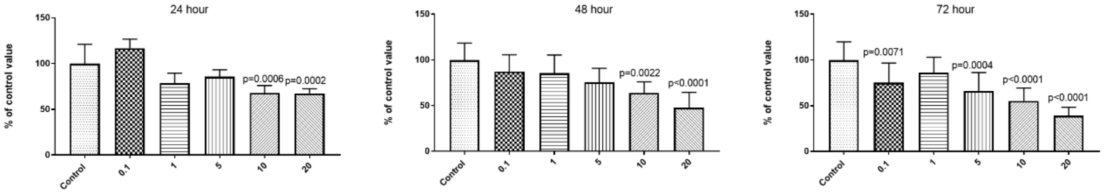

Viability

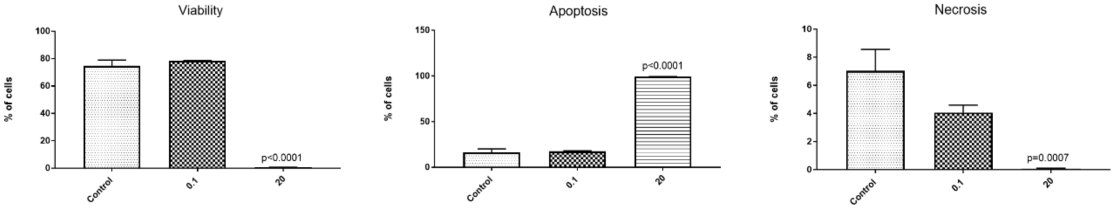

Cell Cycle

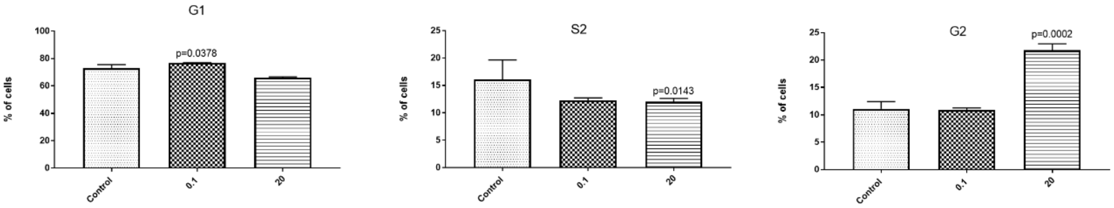

Figure S56. Impact of JAC-E (ethanolic extract of convectionally-dried sample from plants elicited with 10 µM of jasmonic acid) on cytotoxicity (MTT and NR method), type of cell death (Annex V and PI test) and individual phases of the cell cycle of the healthy prostate epithelial cells (HPrEC ATCC® PCS-440-010 ™ cell line).

MTT

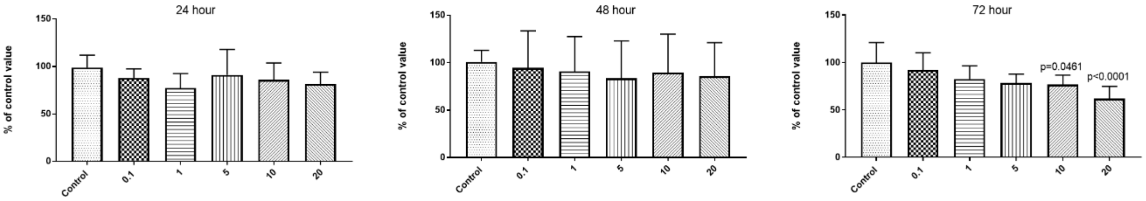

NR

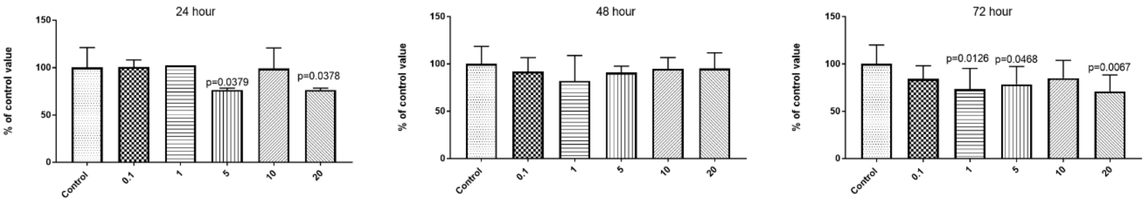

Viability

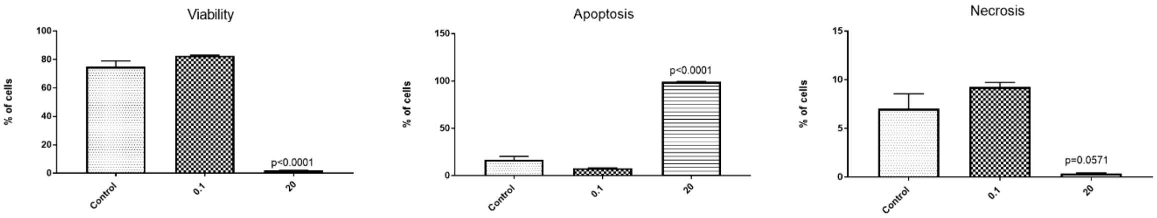

Cell Cycle

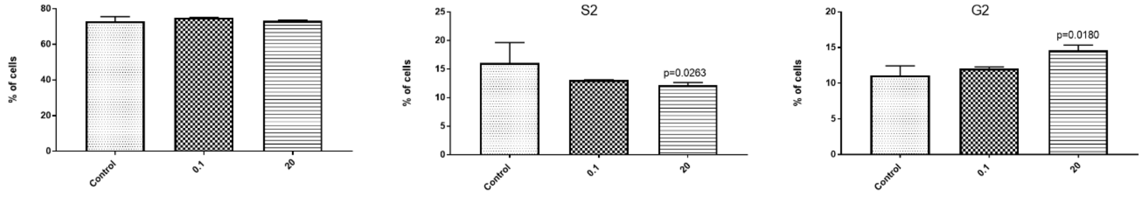

Figure S57. Impact of YEC-E (ethanolic extract of convectionally-dried sample from plants elicited with 0.1% yeast extract) on cytotoxicity (MTT and NR method), type of cell death (Annex V and PI test) and individual phases of the cell cycle of the healthy prostate epithelial cells (HPrEC ATCC® PCS-440-010 ™ cell line).

MTT

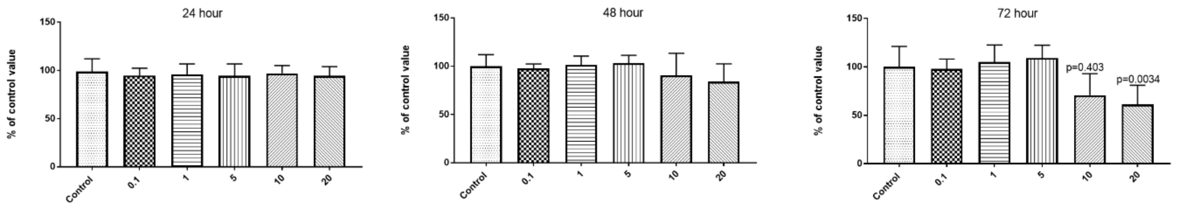

NR

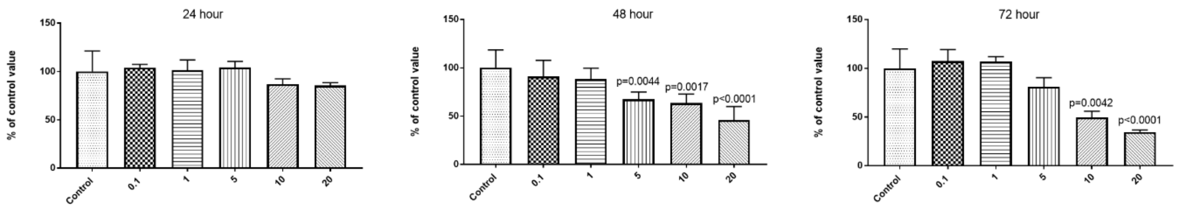

Viability

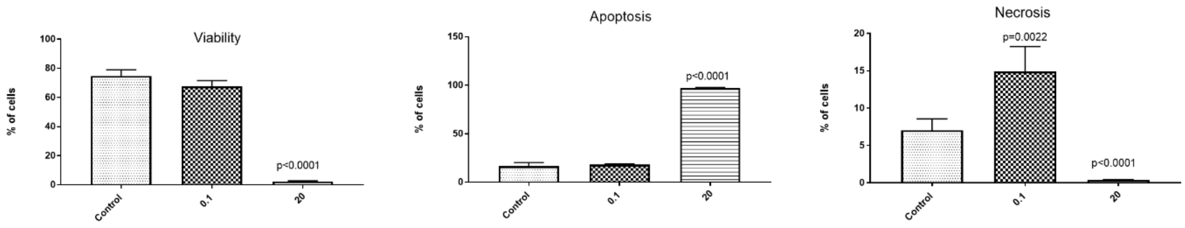

Cell Cycle

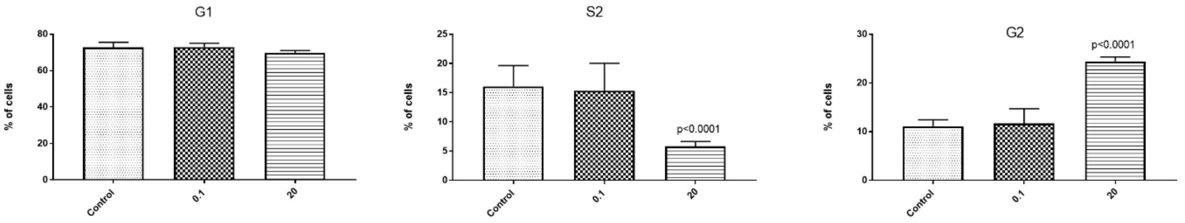

Figure S58. Impact of CC-GD extract (sample after the *in vitro* digestion of control convectionally-dried sample) on cytotoxicity (MTT and NR method), type of cell death (Annex V and PI test) and individual phases of the cell cycle of the healthy prostate epithelial cells (HPrEC ATCC® PCS-440-010 ™ cell line).

MTT

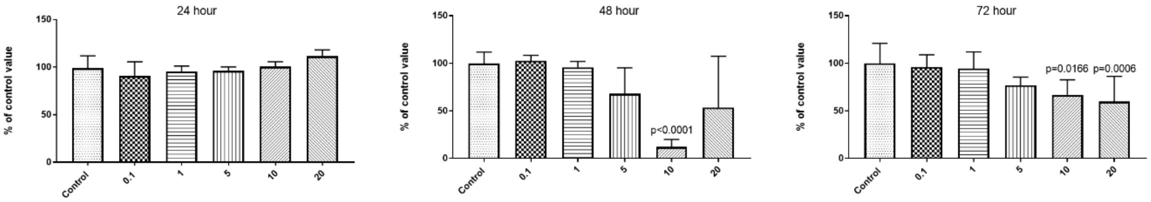

NR

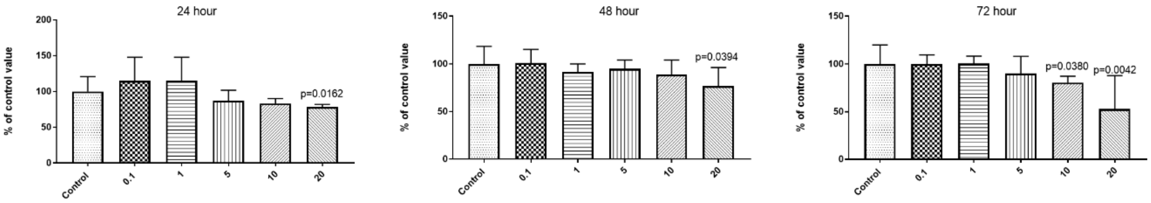

Viability

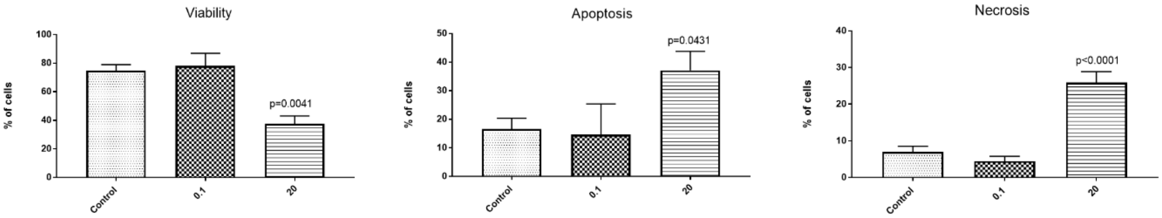

Cell Cycle

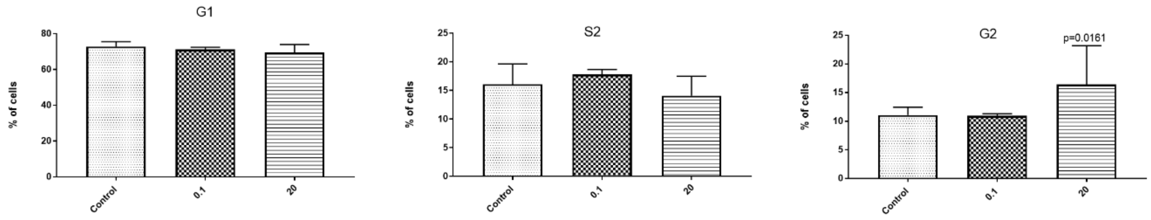

Figure S59. Impact of JAC-GD (sample after the *in vitro* digestion of convectionally-dried sample from plants elicited with 10 µM of jasmonic acid) on cytotoxicity (MTT and NR method), type of cell death (Annex V and PI test) and individual phases of the cell cycle of the healthy prostate epithelial cells (HPrEC ATCC® PCS-440-010 ™ cell line).

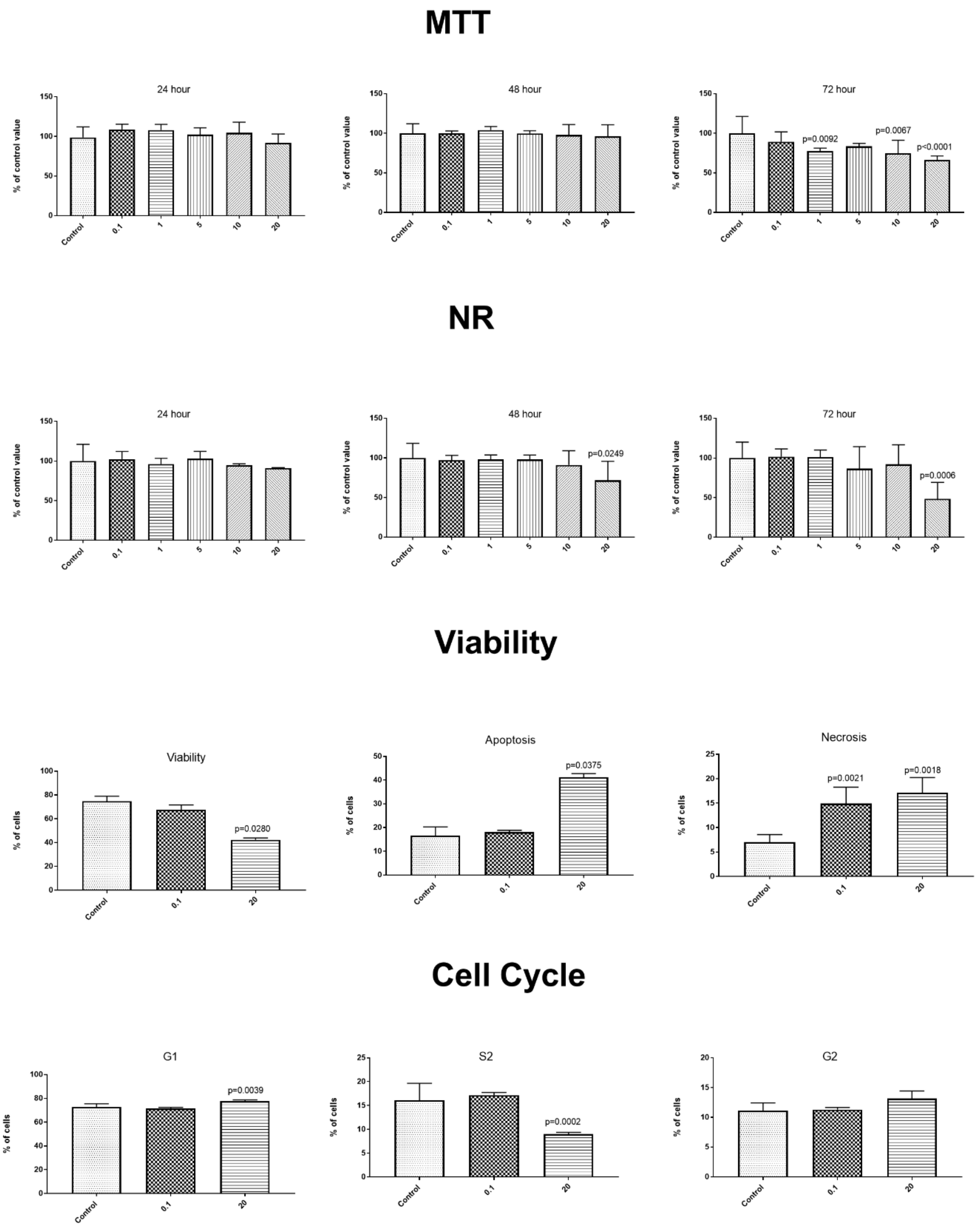

Figure S60. Impact of YEC -GD extract (sample after the *in vitro* digestion of convectionally-dried sample from plants elicited with 0.1% yeast extract) on cytotoxicity (MTT and NR method), type of cell death (Annex V and PI test) and individual phases of the cell cycle of the healthy prostate epithelial cells (HPrEC ATCC® PCS-440-010 ™ cell line).

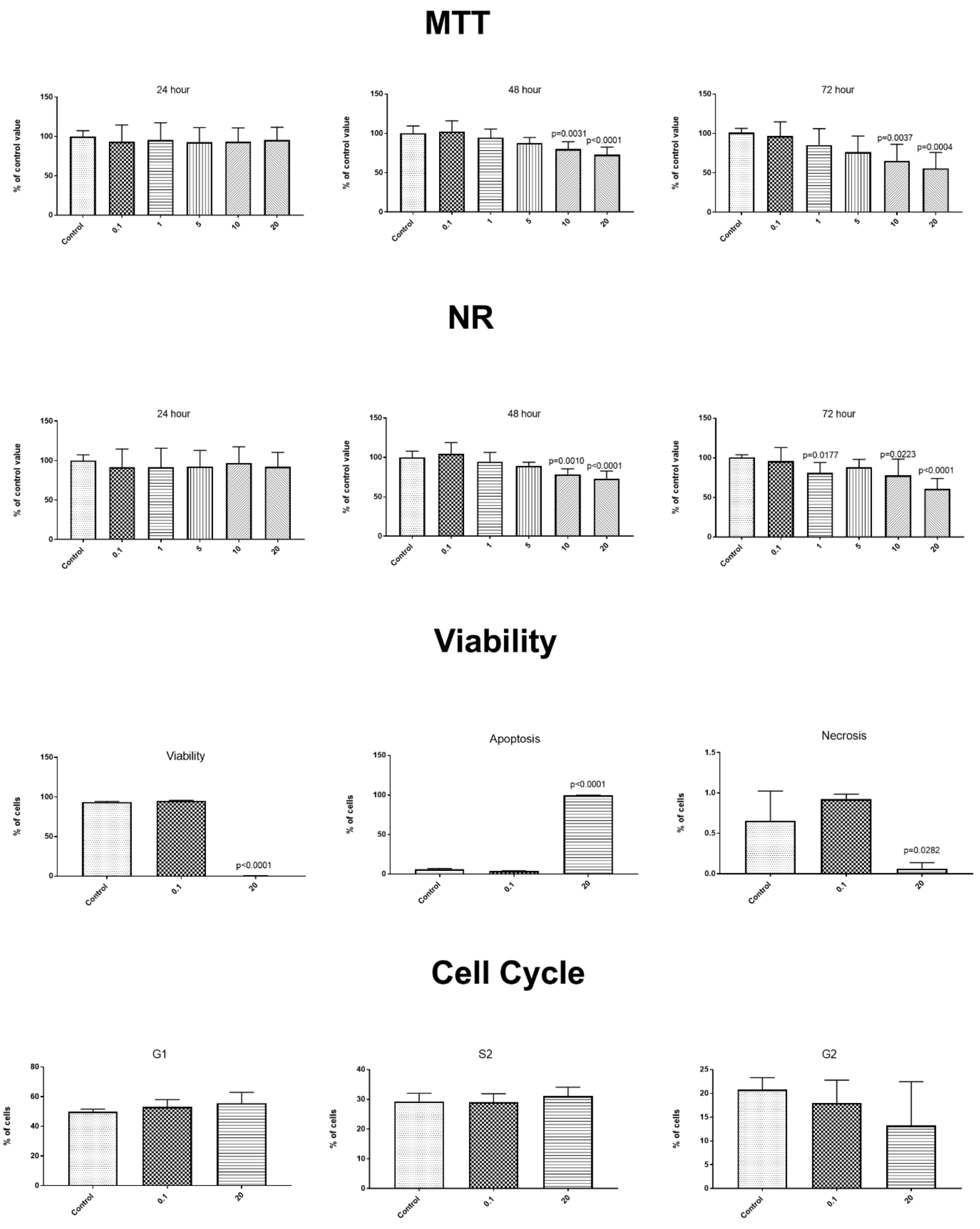

Figure S61. Impact of CC-E (ethanolic extract of control convectionally-dried sample) on cytotoxicity (MTT and NR method), type of cell death (Annex V and PI test) and individual phases of the cancer gastric epithelial NCI-N87 (ATCC® CRL5822 ™) cell line.

MTT

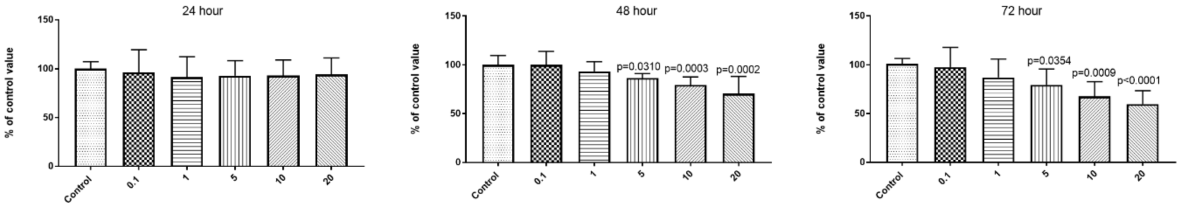

NR

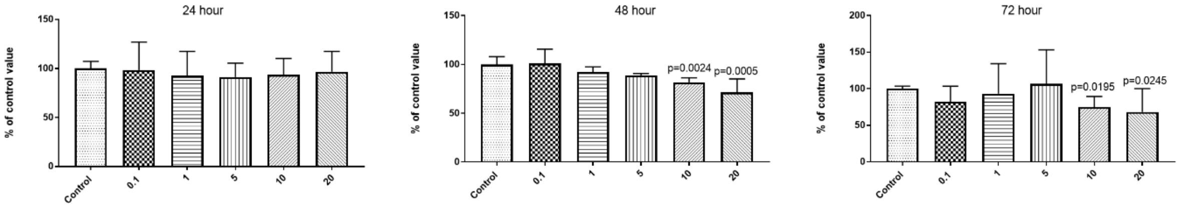

Viability

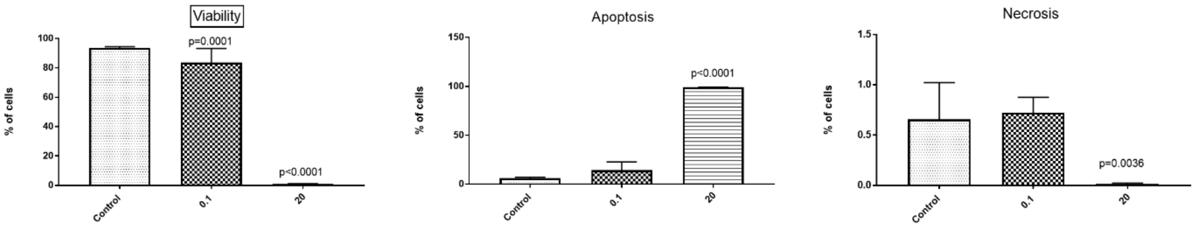

Cell Cycle

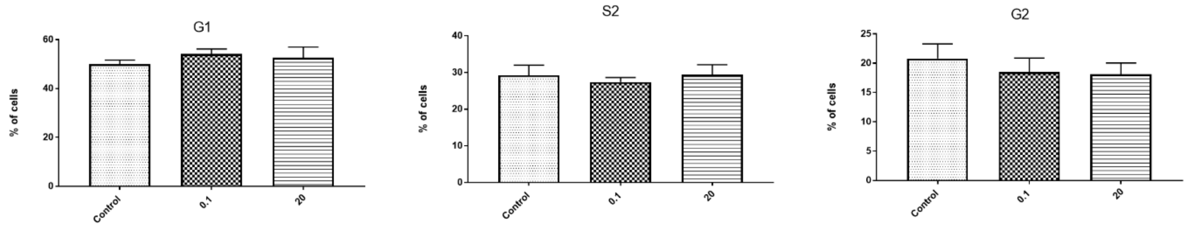

Figure S62. Impact of JAC-E (ethanolic extract of convectionally-dried sample from plants elicited with 10 µM of jasmonic acid) on cytotoxicity (MTT and NR method), type of cell death (Annex V and PI test) and individual phases of the cancer gastric epithelial NCI-N87 (ATCC® CRL5822 ™) cell line.

MTT

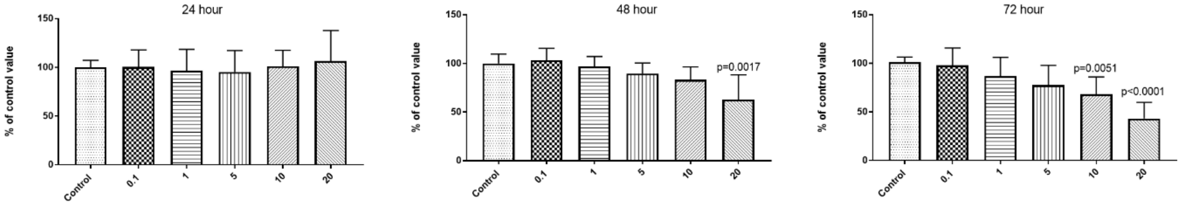

NR

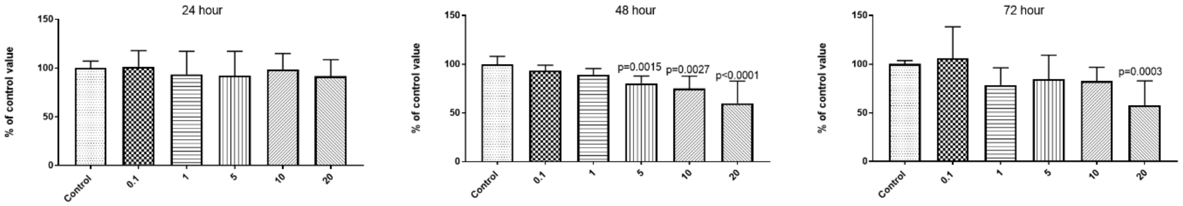

Viability

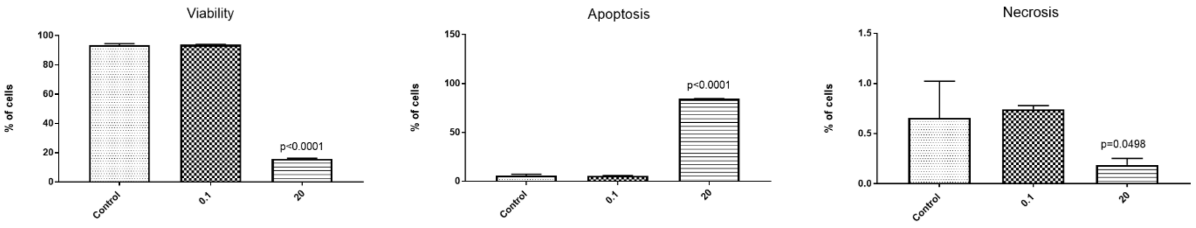

Cell Cycle

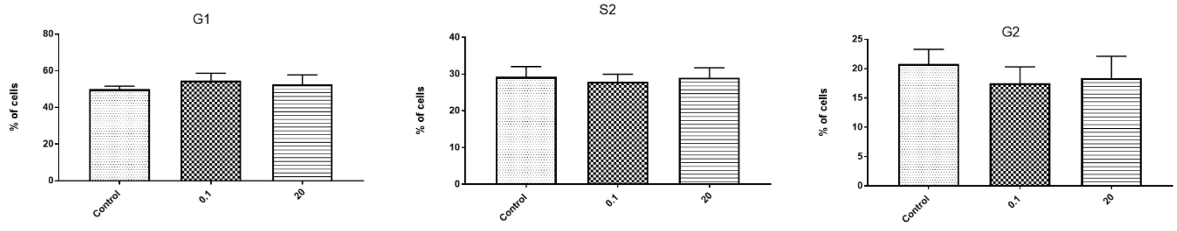

Figure S63. Impact of YEC-E (ethanolic extract of convectionally-dried sample from plants elicited with 0.1% yeast extract) on cytotoxicity (MTT and NR method), type of cell death (Annex V and PI test) and individual phases of the cancer gastric epithelial NCI-N87 (ATCC® CRL5822 ™) cell line.

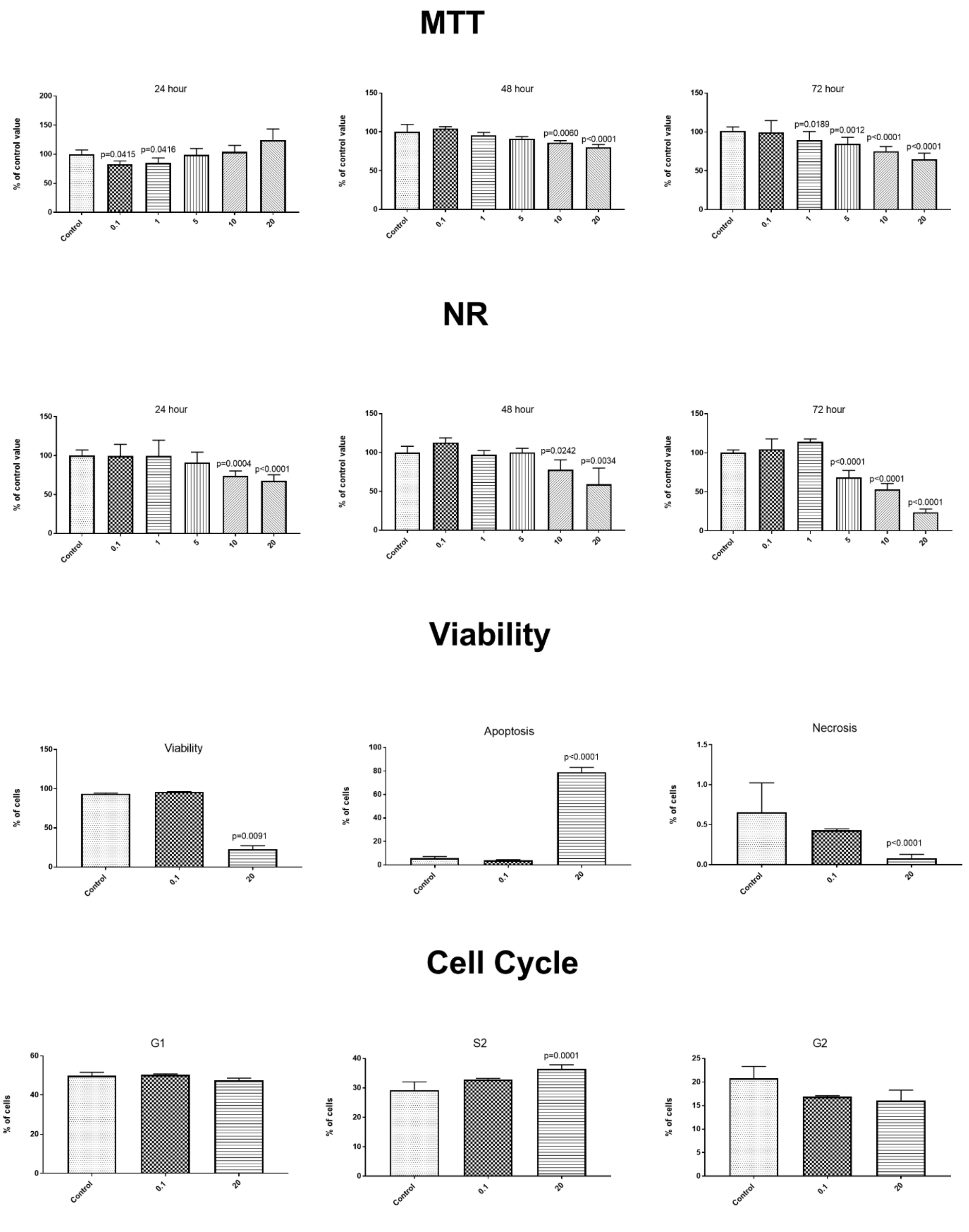

Figure S64. Impact of CC -GD extract (sample after the *in vitro* digestion of control convectionally-dried sample) on cytotoxicity (MTT and NR method), type of cell death (Annex V and PI test) and individual phases of the cancer gastric epithelial NCI-N87 (ATCC® CRL5822 ™) cell line.

MTT

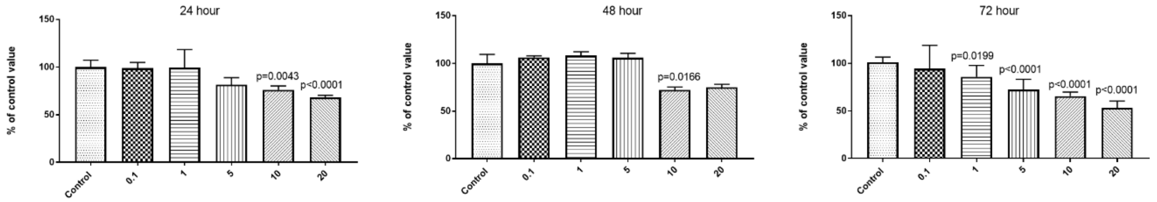

NR

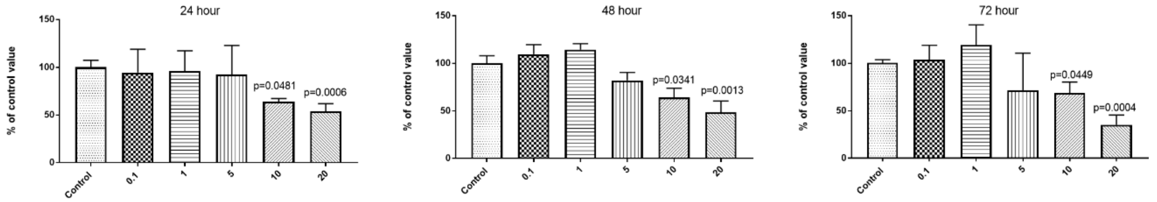

Viability

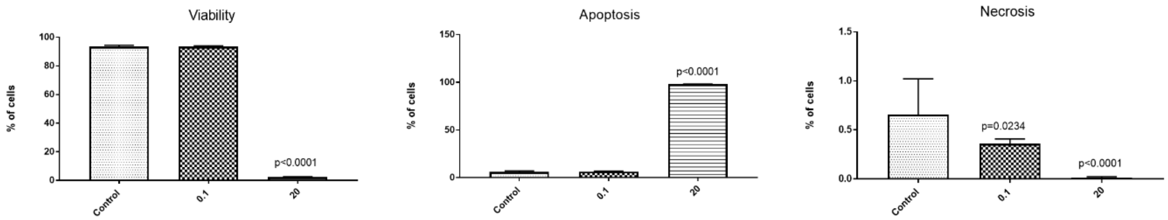

Cell Cycle

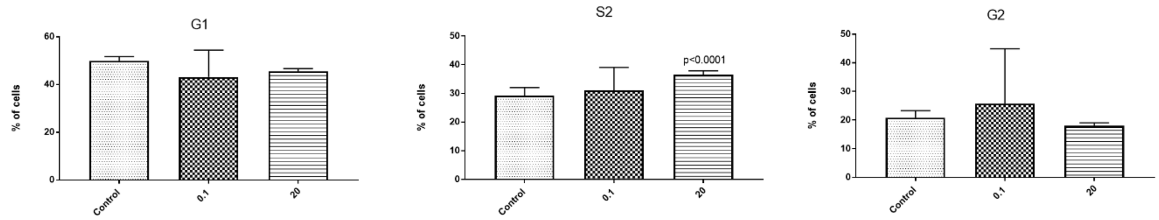

Figure S65. Impact of JAC -GD extract (sample after the *in vitro* digestion of convectionally-dried sample from plants elicited with 10 µM of jasmonic acid) on cytotoxicity (MTT and NR method), type of cell death (Annex V and PI test) and individual phases of the cancer gastric epithelial NCI-N87 (ATCC® CRL5822 ™) cell line.

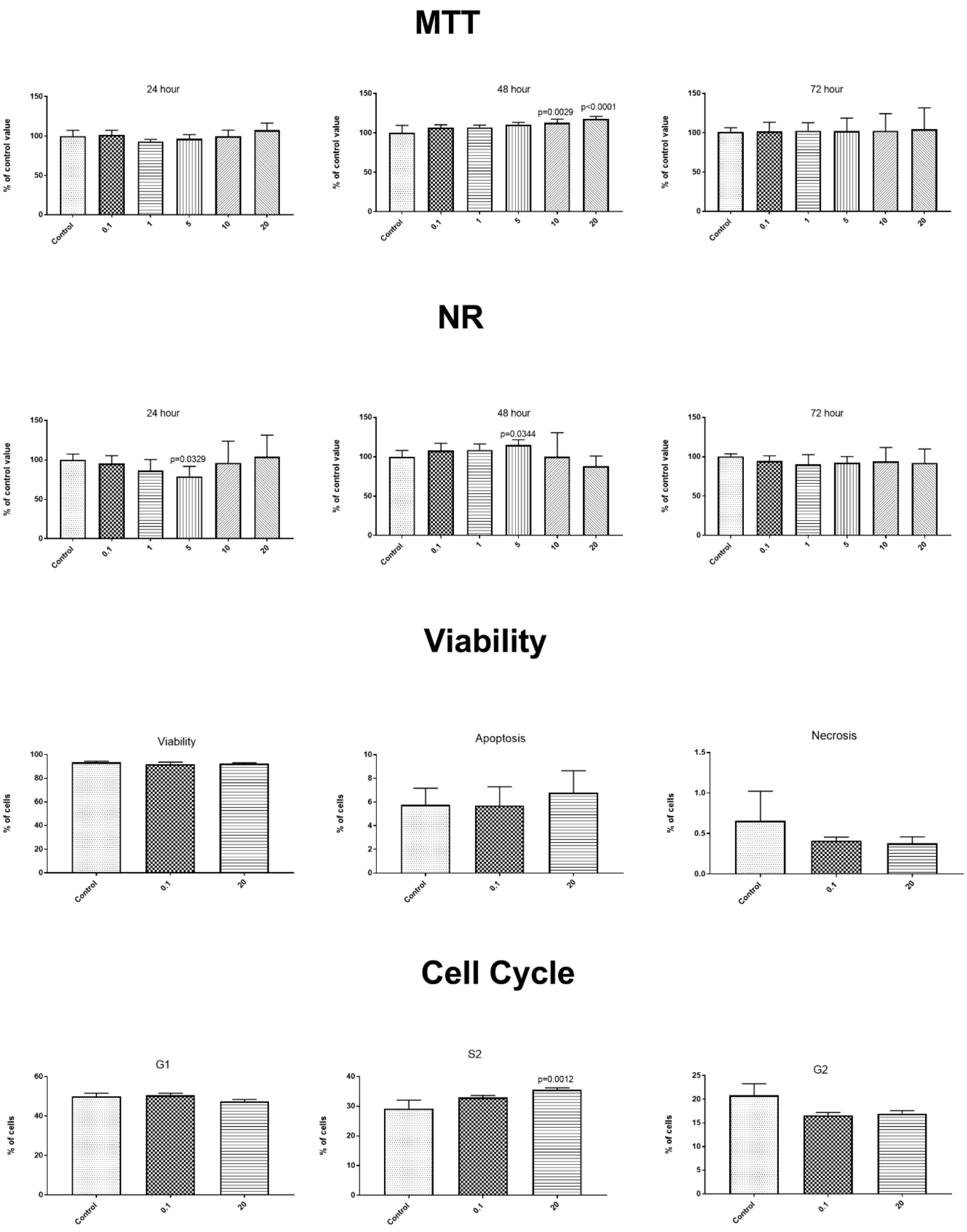

Figure S66. Impact of YEC -GD extract (sample after the *in vitro* digestion of convectionally-dried sample from plants elicited with 0.1% yeast extract) on cytotoxicity (MTT and NR method), type of cell death (Annex V and PI test) and individual phases of the cancer gastric epithelial NCI-N87 (ATCC® CRL5822 ™) cell line.

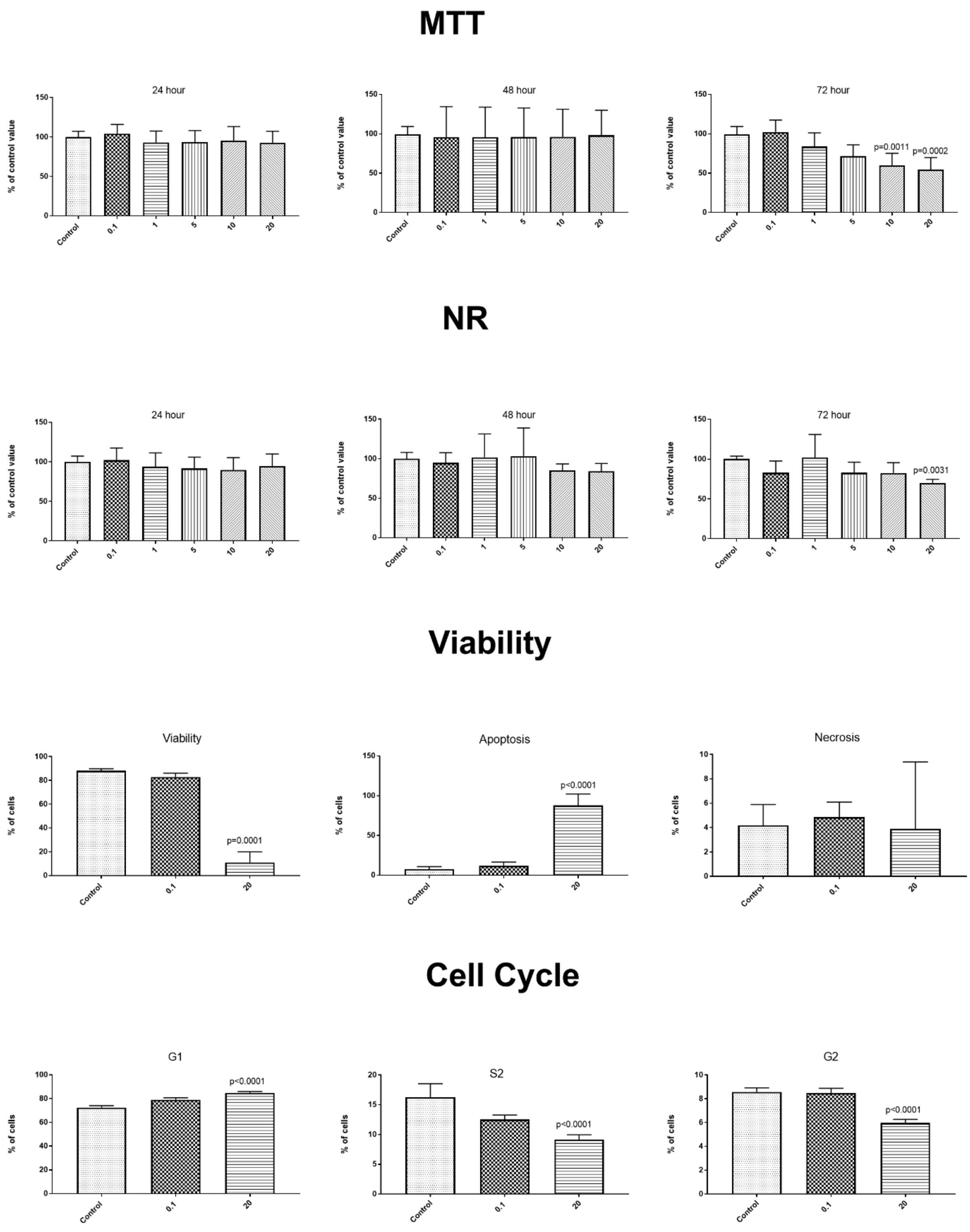

Figure S67. Impact of CC-E (ethanolic extract of control convectionally-dried sample) on cytotoxicity (MTT and NR method), type of cell death (Annex V and PI test) and individual phases of the prostate cancer line VCaP – (ATCC® CRL-2876 ™).

MTT

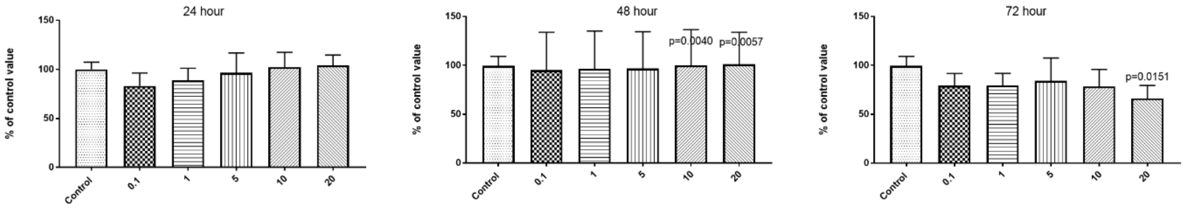

NR

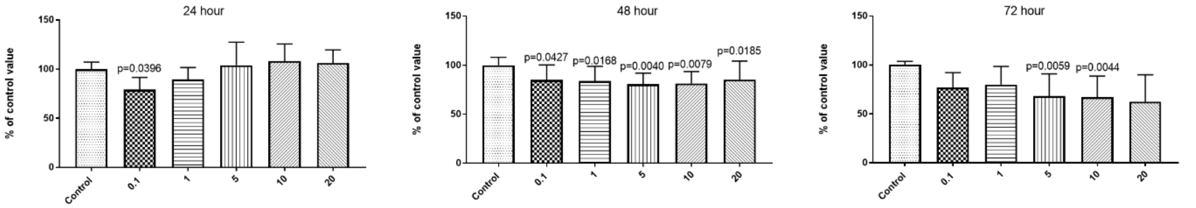

Viability

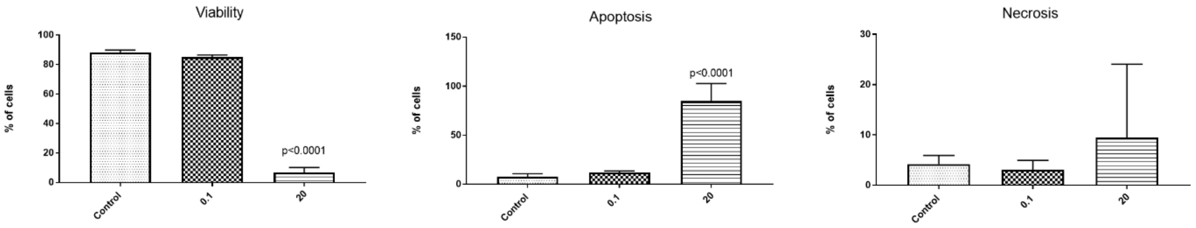

Cell Cycle

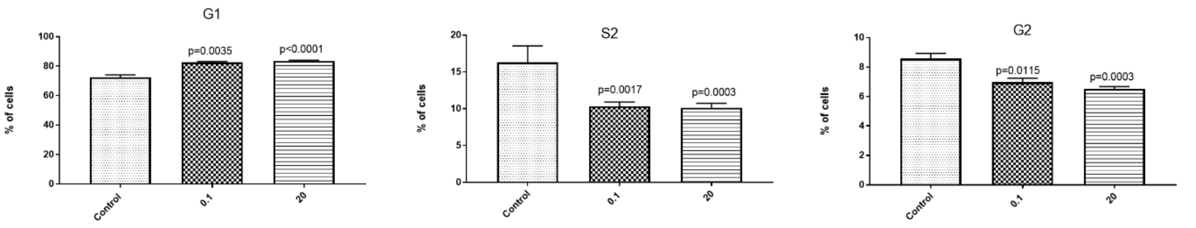

Figure S68. Impact of JAC-E (ethanolic extract of convectionally-dried sample from plants elicited with 10 µM of jasmonic acid) on cytotoxicity (MTT and NR method), type of cell death (Annex V and PI test) and individual phases of the prostate cancer line VCaP – (ATCC® CRL-2876 ™).

MTT

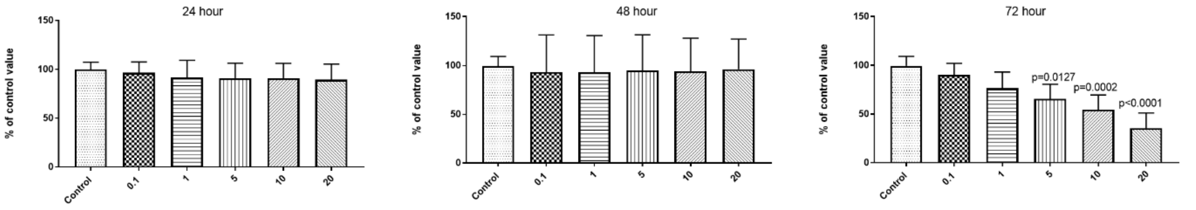

NR

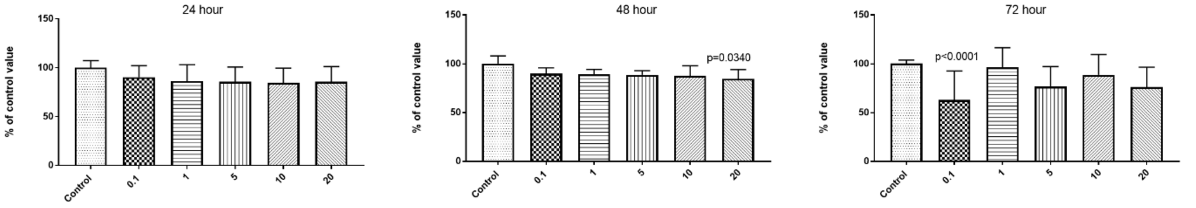

Viability

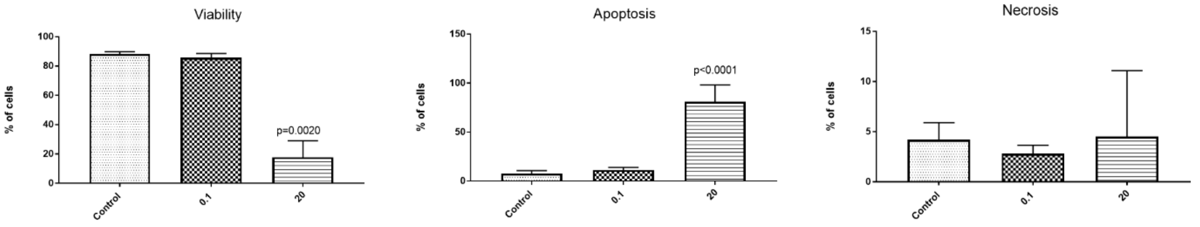

Cell Cycle

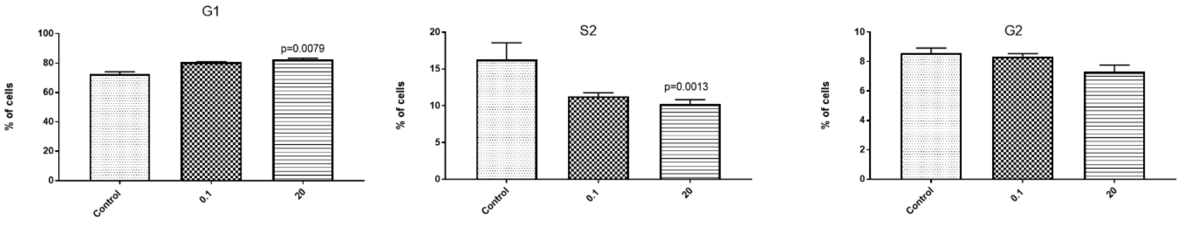

Figure S69. Impact of YEC-E (ethanolic extract of convectionally-dried sample from plants elicited with 0.1% yeast extract) on cytotoxicity (MTT and NR method), type of cell death (Annex V and PI test) and individual phases of the prostate cancer line VCaP – (ATCC® CRL-2876 ™).

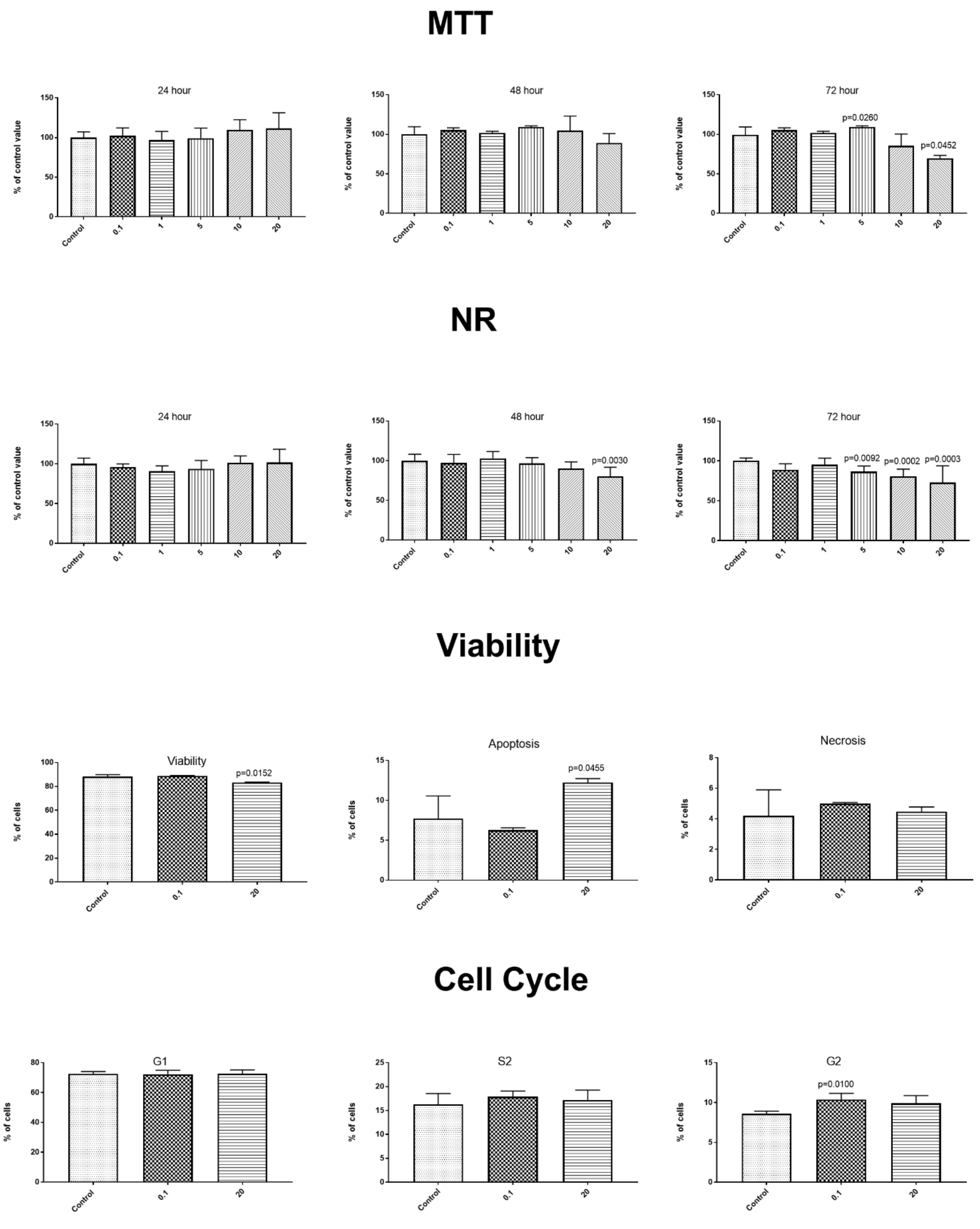

Figure S70. Impact of CC-GD extract (sample after the *in vitro* digestion of control convectionally-dried sample) on cytotoxicity (MTT and NR method), type of cell death (Annex V and PI test) and individual phases of the prostate cancer line VCaP – (ATCC® CRL-2876 ™).

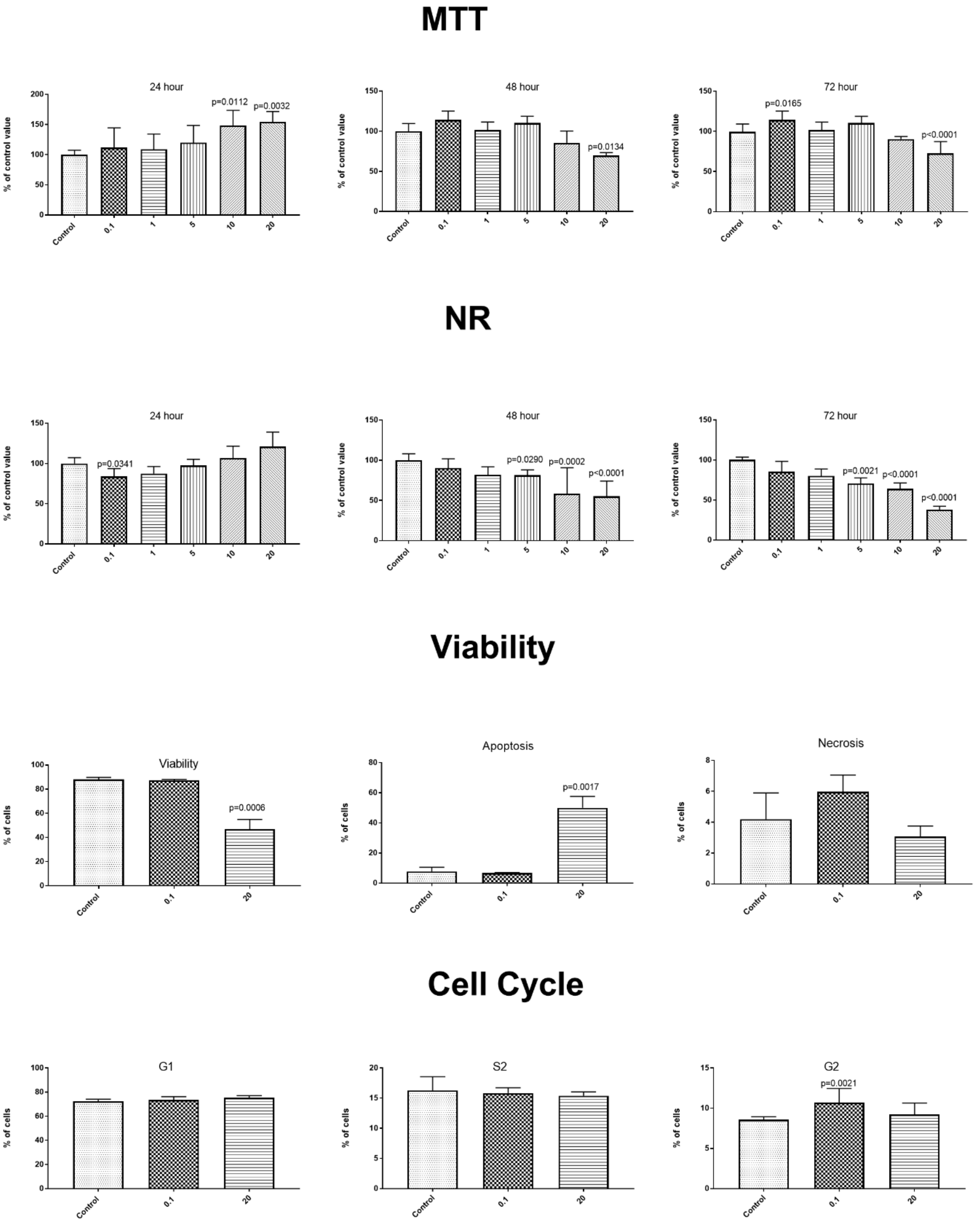

Figure S71. Impact of JAC -GD extract (sample after the *in vitro* digestion of convectionally-dried sample from plants elicited with 10 µM of jasmonic acid) on cytotoxicity (MTT and NR method), type of cell death (Annex V and PI test) and individual phases of the prostate cancer line VCaP – (ATCC® CRL-2876 ™).

MTT

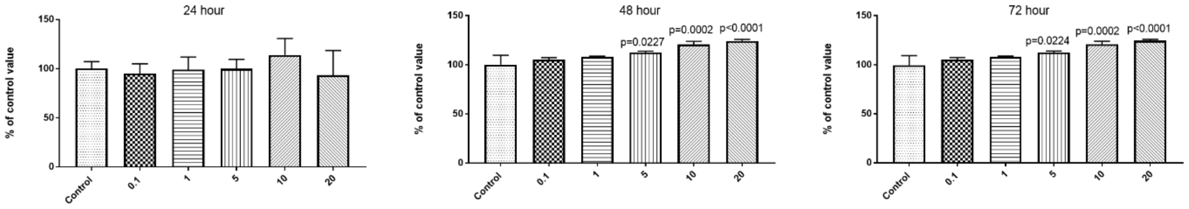

NR

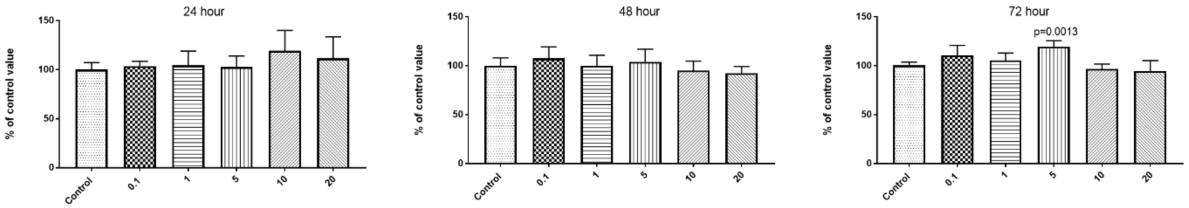

Viability

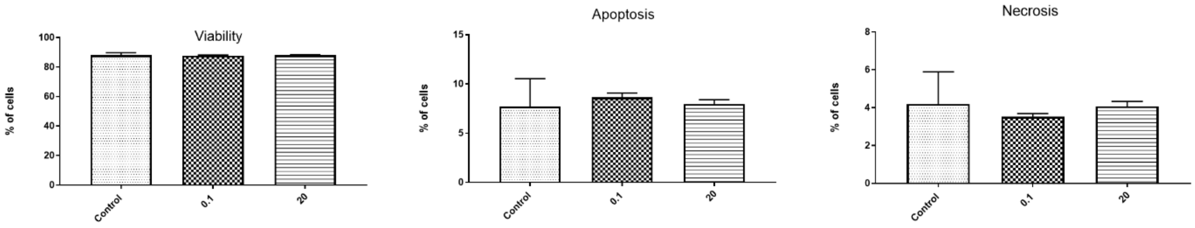

Cell Cycle

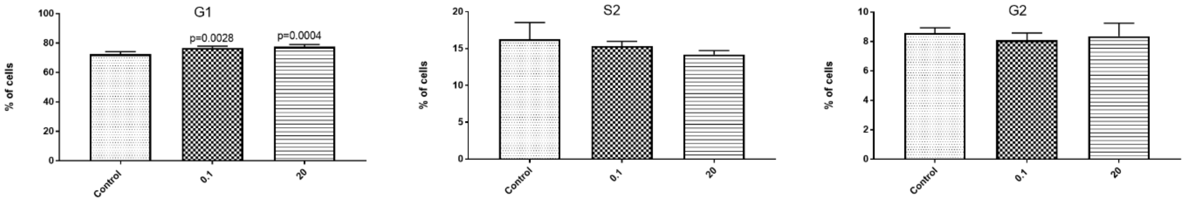

Figure S72. Impact of YEC -GD extract (sample after the *in vitro* digestion of convectionally-dried sample from plants elicited with 0.1% yeast extract) on cytotoxicity (MTT and NR method), type of cell death (Annex V and PI test) and individual phases of the prostate cancer line VCaP – (ATCC® CRL-2876 ™).
